# Pan‐Cancer landscape of protein activities identifies drivers of signalling dysregulation and patient survival

**DOI:** 10.15252/msb.202110631

**Published:** 2023-01-23

**Authors:** Abel Sousa, Aurelien Dugourd, Danish Memon, Borgthor Petursson, Evangelia Petsalaki, Julio Saez‐Rodriguez, Pedro Beltrao

**Affiliations:** ^1^ European Molecular Biology Laboratory European Bioinformatics Institute Cambridge UK; ^2^ Instituto de Investigação e Inovação em Saúde da Universidade do Porto (i3s) Porto Portugal; ^3^ Institute of Molecular Pathology and Immunology of the University of Porto (IPATIMUP) Porto Portugal; ^4^ Graduate Program in Areas of Basic and Applied Biology (GABBA) Abel Salazar Biomedical Sciences Institute, University of Porto Porto Portugal; ^5^ Faculty of Medicine, and Heidelberg University Hospital Institute for Computational Biomedicine, Heidelberg University Heidelberg Germany; ^6^ Faculty of Medicine Institute of Experimental Medicine and Systems Biology, RWTH Aachen University Aachen Germany; ^7^ Institute of Molecular Systems Biology ETH Zürich Zürich Switzerland

**Keywords:** adaptation, cancer genomics, cell signalling, phosphoproteomics, protein activities, Cancer, Computational Biology, Signal Transduction

## Abstract

Genetic alterations in cancer cells trigger oncogenic transformation, a process largely mediated by the dysregulation of kinase and transcription factor (TF) activities. While the mutational profiles of thousands of tumours have been extensively characterised, the measurements of protein activities have been technically limited until recently. We compiled public data of matched genomics and (phospho)proteomics measurements for 1,110 tumours and 77 cell lines that we used to estimate activity changes in 218 kinases and 292 TFs. Co‐regulation of kinase and TF activities reflects previously known regulatory relationships and allows us to dissect genetic drivers of signalling changes in cancer. We find that loss‐of‐function mutations are not often associated with the dysregulation of downstream targets, suggesting frequent compensatory mechanisms. Finally, we identified the activities most differentially regulated in cancer subtypes and showed how these can be linked to differences in patient survival. Our results provide broad insights into the dysregulation of protein activities in cancer and their contribution to disease severity.

## Graphical abstract

Estimation of activity changes for over 500 kinases and transcription factors in more than 1,000 cancer samples and cell lines identifies signalling regulators often dysregulated in cancer associated with patient survival.

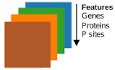

## Introduction

Cancer is a highly heterogeneous disease that is generally caused by the acquisition of somatic genomic alterations, including single nucleotide variants (SNVs), gene copy number variations (CNVs) and large chromosomal rearrangements (Beroukhim *et al*, 2010; Pleasance *et al*, 2010; ICGC/TCGA Pan‐Cancer Analysis of Whole Genomes Consortium, 2020). The Cancer Genome Atlas (TCGA) has led to an in‐depth characterisation of the genomic alterations of more than 10,000 tumours from 33 cancer types (Ding *et al*, 2018; Hoadley *et al*, 2018). However, mutations in key driver genes are just the first steps of a cascade of events that culminate in tumour formation and cancer. These mutations generate the genetic diversity that promotes the acquisition of multiple cancer hallmarks, including chronic proliferation, resistance to cell death and tissue invasion and metastasis (Hanahan & Weinberg, 2011). An understanding of the molecular mechanisms that underpin the development of cancer is critical in order to study cancer biology and to develop therapies.

While somatic alterations and gene expression changes across tumours have been extensively studied, key driver genomic changes in cancer are thought to result in changes in cell signalling including the misregulation of protein kinases and transcription factors (Blume‐Jensen & Hunter, 2001; Yaffe, 2019). As an example, about 40% of melanomas contain the V600E activating mutation in the BRAF kinase, associated with constitutive signalling through the Raf to mitogen‐activated protein kinase (MAPK) pathway and increased cellular proliferation (Davies & Samuels, 2010). Likewise, aberrant transcription factors (TFs) activities are a key feature of cancer cells (Garcia‐Alonso *et al*, 2018). TFs are commonly dysregulated due to genomic alterations in their sequences or in upstream signalling regulatory proteins (Oliner *et al*, 1992; Ohh *et al*, 2000). Because of their role as signalling effectors, aberrant kinase signalling may dysregulate the activities of TFs and alter the expression of their target genes. Consequently, kinases and TFs often accumulate cancer driver mutations, such as TP53 (Rivlin *et al*, 2011) and KRAS (Wang *et al*, 2015), and are the targets of anti‐cancer drugs (Bhagwat & Vakoc, 2015; Bhullar *et al*, 2018).

Due to technical limitations, the study of protein signalling activities has been for many years limited primarily to the study of a few key signalling proteins at a time using antibodies, which was recently expanded to a few hundreds via the use of reverse‐phase protein arrays (RPPA; Li *et al*, 2013). The Clinical Proteomic Tumour Analysis Consortium (CPTAC) has revolutionised the study of cancer proteomes, including proteins and respective post‐translational modifications (PTMs), through the application of mass spectrometry (MS)‐based proteomics (Zhang *et al*, 2019). MS‐based proteomic profiling of human cancers has the potential to uncover molecular insights that might be otherwise missed by genomics‐ and transcriptomics‐driven cancer research. CPTAC enabled to (i) identify additional cancer molecular subtypes (Gao *et al*, 2019; Mun *et al*, 2019), (ii) find that changes at the genomic and transcriptomic level are often buffered at the proteomic level (Zhang *et al*, 2014; Mertins *et al*, 2016; Gonçalves *et al*, 2017; Sousa *et al*, 2019) and (iii) uncover dysregulated signalling pathways by phosphoproteomics data integration (Clark *et al*, 2019).

In efforts to find novel therapeutic opportunities from kinase and TF oncogenic signalling, it is crucial to understand how the activities of these key signalling proteins are changing across tumours. Previous studies found that TF mutations were correlated with transcriptional dysregulation in cancer cell lines and primary tumours and that TF activities can act as predictors of sensitivity to anti‐cancer drugs (Garcia‐Alonso *et al*, 2018). Similar results were found regarding the impact of oncogenic mutations on kinase signalling. However, these studies were focussed on few kinases and cancer types (Guha *et al*, 2008; Guo *et al*, 2008; Creixell *et al*, 2015; Lundby *et al*, 2019). Despite all of these efforts, a systematic Pan‐Cancer analysis of the regulation of kinase and TF activities across tumours is still lacking.

In this study, we compiled multiomics datasets for 1,110 tumours and 77 cell lines to study the regulation of kinases and TFs across tumour types. We estimated the activities of TFs and kinases from the gene expression levels and phosphorylation changes in their targets, deriving activity profiles of 292 TFs and 218 kinases. We used these kinase and TF activities to study the principles of regulation of these signalling proteins by mutations, changes in abundance or phosphorylation. We show how their patterns of activity co‐regulation reflect underlying signalling relationships, and we identify the signalling molecules that show high degree of regulation in each tumour type. Finally, we show how these TF/kinase activities can be predictive of differential survival across patients. The profile of protein activities across over 1,000 patient samples serves as a resource to study the misregulation of signalling across different tumour types.

## Results

### Standardised multiomics pan‐cancer dataset

**Figure 1:**
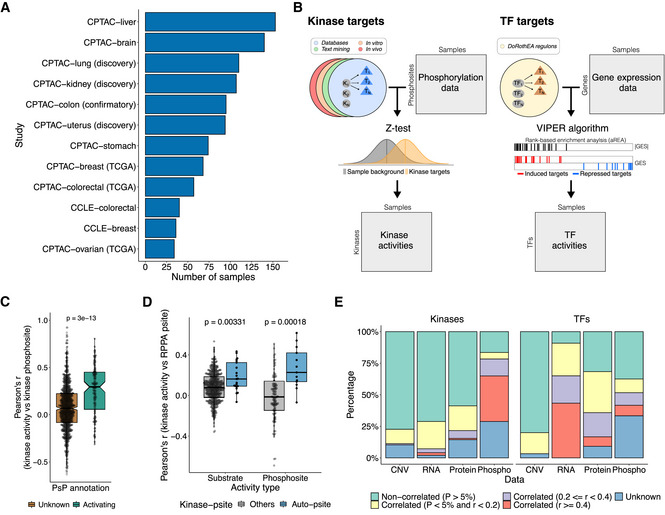
Multiomics atlas and inference of protein activities Number of samples with coverage for all multiomics data by cancer dataset.Schematic representation of kinase and TF activity inference. GES, gene expression signature.Comparison of the Pearson's correlation distributions between the kinase activities and the quantifications of phosphosites (log2 fold‐changes) that mapped to the same kinase, with (*n* = 126) and without (*n* = 793) annotation (activating) in PhosphoSitePlus. A *P*‐value from a Wilcoxon rank‐sum test is shown.Comparison of the Pearson's correlation between the RPPA phosphosites and the kinase activities, for kinase‐phosphosite pairs mapping to the same kinase (auto‐phosphosite) and other pairs. The activities were calculated using the kinase substrates (*n* = 336 and *n* = 21) and the kinase regulatory phosphosites (*n* = 94 and *n* = 13; Materials and Methods). The *P*‐values from Wilcoxon rank‐sum tests are shown.Percentage of kinases and TFs significantly and not significantly correlated with the corresponding CNV, RNA, protein and phosphorylation levels. The proteins without correlations due to lack of data or reduced number of samples (*n* < 10) were labelled as unknown (blue). Data information: The box plots represent the 1^st^, 2^nd^ (median) and 3^rd^ quartiles and the whiskers indicate 1.5 times the interquartile range (IQR). The central notches show the 95% confidence interval around the median and are calculated as median ± 1.58 * (IQR / sqrt(*n*)).

To study the regulation of protein activities of cancer cells, we compiled and standardised multiomics datasets made available by the CPTAC consortium (Fig 1A, Appendix Fig S1A; Materials and Methods). These datasets were comprised of cancer patient samples with matched somatic mutations, gene copy number variation (CNV), mRNA expression, protein abundance, phosphorylation and clinical data from nine tissues: breast (Cancer Genome Atlas Network, 2012b; Mertins *et al*, 2016), brain (Petralia *et al*, 2020), colorectal (Cancer Genome Atlas Network, 2012a; Zhang *et al*, 2014; Vasaikar *et al*, 2019), ovarian (Cancer Genome Atlas Research Network, 2011; Zhang *et al*, 2016), liver (Gao *et al*, 2019), kidney (Clark *et al*, 2019), uterus (Dou *et al*, 2020), lung (Gillette *et al*, 2020) and stomach (Mun *et al*, 2019). In addition, we collected data for breast (Lawrence *et al*, 2015; Lapek *et al*, 2017) and colorectal (Roumeliotis *et al*, 2017) cancer cell lines, for which multiomics data were available (Fig 1A, Appendix Fig S1A; Materials and Methods). In summary, the assembled data includes 1,110 tumours samples and 77 cell lines, of which 1,008 samples (932 tumours and 76 cell lines) have available data for all data types used in this study. For each analysis, we used all samples having data for the types of omics measurement required, which results in small variations of the total number of samples used.

We first calculated correlations between each protein and phosphosites that mapped to the same protein, in up to 1,008 samples. Across protein‐phosphosite pairs, there is a correlation of 0.49 (*P*‐value < 2.2 × 10e‐16; Appendix Fig S1B), which is similar when calculated per tumour type (Appendix Fig S1C), and is in agreement with previous studies (Arshad *et al*, 2019). This result shows that the changes in phosphorylation are, to some extent, confounded by the changes in the corresponding protein abundance (Wu *et al*, 2011). To be able to focus on phosphorylation changes that are not driven primarily by protein abundance differences, we regressed out matched protein abundance from the phosphorylation data in our compiled dataset (Appendix Fig S1B and C; Materials and Methods).

### Landscape of protein activities in cancer

The genomics characterisation of tumour samples has so far been primarily focussed on stratifying samples by their mutational profiles or changes in abundance of specific bio‐molecules such as transcripts, protein or phosphorylation states. We and others have shown that changes in phosphorylation and gene expression levels can be used to infer the activation states of protein kinases and TFs (Casado *et al*, 2013; Ochoa *et al*, 2016; Hernandez‐Armenta *et al*, 2017; Garcia‐Alonso *et al*, 2018). Based on these methods, we set out to define the landscape of kinase/TF activity patterns across these tumour samples.

The kinase activities were estimated from the protein abundance‐corrected phosphorylation data using a *z*‐test (Hernandez‐Armenta *et al*, 2017; Fig 1B; Materials and Methods). Briefly, the activity of a given kinase in a sample is estimated by comparing the changes in phosphorylation of its substrates with changes in all other phosphosites. Similarly, the activation state of TFs was inferred from the changes in gene expression of their known transcriptional targets using the DoRothEA regulons (Garcia‐Alonso *et al*, 2019) coupled with the VIPER algorithm (Alvarez *et al*, 2016; Fig 1B; Materials and Methods). In total, we estimated the activities of 292 TFs across 1,187 cancer samples (1,110 primary tumours and 77 cell lines; Dataset EV1). For the estimation of kinase activities, we evaluated different lists of kinase substrates from repositories (e.g. PhosphositePlus; Hornbeck *et al*, 2015), computational text mining (preprint: Bachman *et al*, 2019), kinase inhibitor experiments (Hijazi *et al*, 2020) or phosphorylation of cell extracts (Sugiyama *et al*, 2019; Appendix Fig S2A and B). We tested each list in a compilation of phosphoproteomic experiments where kinase regulation is known (Hernandez‐Armenta *et al*, 2017; Materials and Methods) keeping those from repositories and text‐mining as the most accurate (Appendix Fig S3A and B). After applying this approach, we inferred the activities of 218 kinases across 980 samples (930 tumours and 50 cell lines; Dataset EV1, Materials and Methods).

For some kinases, there are phosphosites within the kinase itself that are known to activate or inhibit it. As a validation, we correlated the estimated activity scores with the quantifications of activating phosphosites, finding the expected higher correlation when compared to phosphosites without annotation (Fig 1C). A similar trend was observed when excluding the kinase auto‐regulatory phosphosites before re‐estimating the activities (Appendix Fig S3C). Finally, we benchmarked the kinase activity scores using reverse phase protein array (RPPA) data from the TCGA programme. We first evaluated the agreement between the MS‐based and the RPPA‐based phosphosite quantifications and found that phosphosite pairs corresponding to the same phosphosite show higher correlations than random pairs (Appendix Fig S3D). Then, we found that the RPPA phosphosites correlate significantly better with the activity of the kinase bearing the phosphosites than with other kinase activities (Fig 1D).

The activity profiles of kinase and TFs across a large number of samples allow us to ask how these activities are themselves regulated. We first selected 99 kinases and 120 TFs that are strongly regulated (i.e. activities > 96.7^th^ percentiles) in at least 5% of all samples (Appendix Fig S3E). We then correlated across samples the predicted activities with the measured changes in gene copy number (CNV), mRNA, protein and phosphorylation levels of the respective protein (Dataset EV2). We observed that 55% of kinase activities correlated with their phosphorylation state, 27% with changes in protein abundance and very few correlated with changes in their mRNA (Fig 1E). Contrary to this, TF activities are most often correlated with changes in abundance of the TF, as measured by RNA (91%) or protein (59%), with fewer cases of significant correlations with phosphorylation levels (29%; Fig 1E). The CNV levels were overall poor indicators of changes in kinase and TF activities. TF phosphosites predicted to be important for function (Ochoa *et al*, 2020) are more likely to show significant correlations with the TF activity (Appendix Fig S3F).

Overall, these results showed that our kinase activity estimates are likely to capture kinase regulatory events across different tumour types and therefore the usefulness of our multiomics atlas to study kinase signalling in cancer.

### Impact of genetic variation on protein abundance and activities

The large number of cancer samples in this study constitutes a resource to measure the effects of genetic alterations, that is somatic mutations and CNVs, on protein abundances and activities. We first set out to assess the effects of CNVs on the mRNA and protein abundances. As described previously, the CNVs showed a stronger correlation with the mRNA than with the protein levels (Appendix Fig S4A and B), highlighting mechanisms of post‐transcriptional control and gene dosage buffering at the protein level (Gonçalves *et al*, 2017; Sousa *et al*, 2019). We then extended the analysis to globally assess the effects of mutations (Materials and Methods), and we found that proteins carrying loss‐of‐function (LoF) alterations, including frameshift, nonsense, splice site and stop codon loss, caused on average a significant decrease in protein abundance. This was not observed with in‐frame and missense mutations (Appendix Fig S4C). To validate the decrease in protein abundance for LoF mutations, we confirmed that this was also recapitulated in a proteomic dataset with 125 cancer cell lines (CCLs) from the NCI60 and CRC65 panels (Frejno *et al*, 2020; Appendix Fig S4D; Materials and Methods). These observations confirm that the genetic alterations are often recapitulated at the protein level as captured by the MS data.

We next looked at the impact of genetic alterations on TF and kinase activity estimates. Across all samples, we looked at all cases where a TF or kinase carries a mutation, segregated by mutation type as above (e.g. missense, frameshift and nonsense). We then asked whether TFs and kinases carrying such mutations would have, on average, differences in their predicted activities as measured by changes in their targets. On average, we did not observe reduced predicted activity for proteins carrying different types of mutations in the tumour samples (Appendix Fig S5A). Overall, we only found a modest average decrease in predicted activities for proteins carrying frameshift mutations in cell lines (Appendix Fig S5B). This observation did not depend on the predicted deleterious impact of the mutations (Appendix Fig S5C and D) nor on the purity of the tumour samples (Appendix Fig S5E).

To further characterise this result, we focussed on highly mutated cancer genes. As an example, we investigated the impact of mutations BRAF^V600E^, KRAS^G12D^ and KRAS^G12C^ on the predicted activities of proteins from the MAPK/ERK signalling transduction pathway (Materials and Methods). Across all samples, BRAF^V600E^ and KRAS^G12D^ mutations were not significantly associated with changes in activity of key pathway components (Appendix Fig S6A). For BRAF^V600E^, we found instead that CDK1 and CDK7 were more active in samples carrying the mutation (FDR < 5%; Appendix Fig S5G). For samples having the KRAS^G12C^, we found a consistent increase in predicted activity of kinases in the MAPK pathway (Appendix Fig S6A). These results suggest that samples carrying KRAS^G12D^ or BRAF^V600E^ mutation will often have kinase activity levels that have adapted to the mutational state without a higher level of activation of the pathway.

In order to generate hypotheses for the lack of pathway activation in the samples with BRAF^V600E^ activating mutation, we selected the 19 samples with this mutation and compared those where BRAF activity was increased (six samples) with those where it was predicted to be decreased (eight samples). We reasoned that phosphatase levels could be a potential mechanism explaining a downregulation of the pathway activity in the presence of an activating mutation. We therefore measured the fold change in phosphatase mRNA levels in these two groups of samples. Of 237 phosphatases tested, 74% tend to have higher expression levels in the samples with low BRAF activity, 17 of these have a significant difference across the two groups of samples with a false discovery rate set at 15% (Appendix Fig S6B). This suggests that overexpression of phosphatases in the mutated samples could be a plausible mechanism that explains the lack of activation.

**Figure 2:**
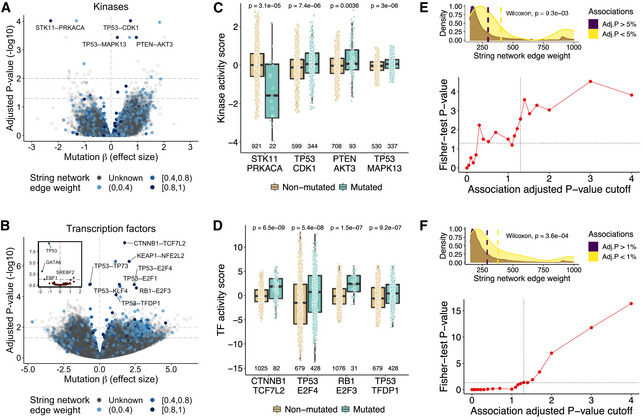
Genetic associations with protein activities A
Volcano plot displaying the associations between the mutational status of genes and the activity of kinases. The x‐axis contains the mutation coefficients (effect sizes) and the y‐axis the adjusted *P*‐values. The vertical line in the x‐axis is at x = 0 and separates the positive (right) from the negative (left) associations. The horizontal lines in the y‐axis represent the points of statistical significance in the plot (adjusted *P*‐values < 0.05 and < 0.01). The associations are represented in the form of a mutated gene—kinase. The colour gradient represents the string network edge weight interval of the pair (grey if the pair is not in the string network).B
Same as (A) for the TFs. The inner plot shows the effects of TF mutations on their own activities.C, D
Examples of the genetic associations highlighted in the volcano plots. The x‐axis represents the associations and the y‐axis the protein activities. The colours stratify the samples by their mutational status in the respective genes. The outliers (defined as the data points beyond Q1‐1.5*IQR and Q3 + 1.5*IQR, where Q1 and Q3 are the first and third quartiles and IQR is the interquartile range) were removed from the distributions for representation purposes. The number of protein activity quantifications (including outliers) is shown beneath each boxplot. The *P*‐values from Wilcoxon rank‐sum tests comparing both distributions are shown. All data points (including outliers) were used to calculate the *P*‐values.E
Top panel. Density plots comparing the edge weight distributions in the string network of the significant and non‐significant association pairs obtained with the kinases. The vertical lines in the x‐axis show the mean edge weight of the significant and non‐significant association pairs. Bottom panel. Enrichment of the associations in the string network (edge weight > 850) along multiple cut‐offs of statistical significance. The x‐axis shows the adjusted *P*‐value cut‐offs (−log10) and the y‐axis the Fisher‐test *P*‐values (−log10). The dashed lines represent the points of statistical significance in the x‐axis and y‐axis (adjusted *P*‐value < 0.05).F
Same as (E) for the TFs.

We then extended the analysis by systematically associating the activity of kinases and TFs with the recurrent mutational status of any given gene mutated in at least five tumour samples (Materials and Methods). As seen for the BRAF example, we did not observe any case where recurrent mutation of the kinase itself was associated with a significant change in its activity as measured by the phosphorylation of its substrates. This indicates that there is significant adaptation of the signalling state of the cell after mutations. On the contrary, we found 193 significant associations (FDR < 5%) between mutations in other genes and changes in kinase activity levels (Fig 2A; Dataset EV3). For example, samples with mutations on STK11 (serine/threonine kinase 11) do not show a pronounced change in activity of STK11 substrates but have decreased activity for PRKACA kinase, a known activator of STK11 (FDR = 9.6e‐5; combined string network weight = 0.94; Fig 2C). Other examples include increased activity for CDK1 and MAPK13 in samples with mutations in TP53 and for AKT3 when PTEN is mutated (FDR < 5%; Fig 2C). Unlike for kinases, we found several cases where the mutation of a TF was associated with a change in its own activity as is the case for mutations in TP53, GATA6, SREBF2 and EBF1 (FDR < 5%; Fig 2B—inner plot, Appendix Fig S7; Dataset EV3). In addition, we found 11,128 significant associations between a mutated gene and a changed TF activity (FDR < 5%; 1,087 for FDR < 1%; Fig 2B—outer plot; Dataset EV3), including increased activity for E2F4 and TFDP1 coupled with TP53 mutation (Fig 2D).

We replicated the associations between mutations and kinase activity using the RPPA antibody measurements of phosphosites in kinases that can report on kinase activities. We were able to measure the association strength for 29 gene‐kinase pairs that overlap with those measured here. Across these associations, we find a correlation of 0.57 (*P* = 0.0012) for the estimated association strength (beta value). While there is much variation, it is not unexpected given the differences in methodology and samples. There are only 2% of samples (95 out of 4,133) that overlap between the two analyses, and as such, this analysis shows that the associations can replicate in independent datasets.

Pan‐Cancer analyses can result in spurious associations driven by batch effects or molecular differences between tissues. We took into account these effects by including the tissue type as a covariate in our models (Materials and Methods). In this way, associations that would have been driven by the tumour type would be suppressed. As a further validation, we have repeated the associations between genetic variants and kinase/TF activities at the tumour type level. We found that the effect size and significance of these associations tend to be highly correlated with the Pan‐Cancer associations (Appendix Fig S8A and B).

The association between mutated genes and altered protein activity contain several examples of previously known functional relationships. To evaluate this more broadly, we confirmed that our predicted associations were enriched in protein–protein functional associations annotated in the STRING database, for both the kinases and the TFs (*P*‐value < 5%; Fig 2E and F—top panel). We also performed an enrichment analysis using the STRING network along multiple cut‐offs of adjusted *P*‐values (Materials and Methods). The −log10 transformed *P*‐values from the enrichment test increased as the association cut‐offs were incremented (Fig 2E and F—bottom panel), validating the generality of the significant associations. Overall, the genetic associations found are enriched in previously known functional associations, containing potential novel regulatory relationships for future experimental exploration.

### An atlas of kinase and TF regulation in cancer

**Figure 3:**
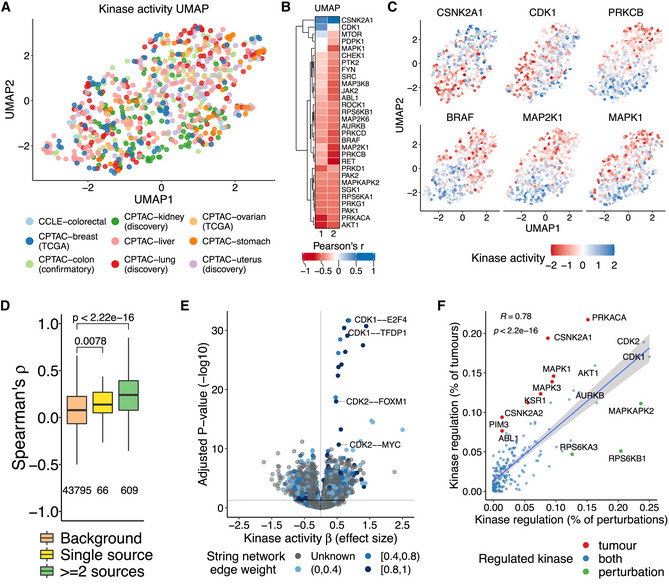
Regulation of protein activities in tumours and human perturbations UMAP projection of the kinase activity matrix (kinases as variables). The samples are coloured by experimental study.Pearson correlation coefficient between the UMAP projections and the activity of non‐redundant highly variable kinases.Kinase activity gradient along the samples for a selection of the kinases shown in (B).Spearman's rank correlation coefficient between the activities of kinases known to co‐regulate each other. The pairwise kinase co‐regulatory relationships were obtained from the OmniPath database and stratified by their presence in the OmniPath's sources (as single source or in at least two different sources). We only kept activating and consensual interactions along the sources. The background corresponds to kinase pairs without known co‐regulation events. The distributions were compared with the background using Wilcoxon rank‐sum tests. The box plots represent the 1^st^, 2^nd^ (median) and 3^rd^ quartiles and the whiskers indicate 1.5 times the interquartile range (IQR).Associations between the activity of kinases and TFs. The x‐axis contains the kinase coefficients (effect sizes) and the y‐axis the adjusted *P*‐values. Each association is represented in the form of kinase—TF. The colour gradient represents the edge weight of the pair in the string network (grey if not present). The vertical line in the x‐axis is at x = 0 and separates the positive (right) from the negative (left) associations. The horizontal line in the y‐axis represents the point of statistical significance in the plot (adjusted *P*‐values < 0.05).Linear regression between the percentage of samples where the kinase is regulated in the perturbed conditions and in the tumour samples. The trend line and the Pearson's *r*, with the respective *P*‐value, are shown. In red and green are the kinases preferably regulated in the tumours and in the conditions, respectively. In blue are the kinases regulated in both. The 95% confidence interval around the regression line is represented by the shaded area.

The estimation of kinase and TF activities across a large set of tumour samples from different tissues provides a first look at the space of tumour signalling states as measured by hundreds of regulators. We projected the activity profiles in a lower‐dimensional space using the uniform manifold approximation and projection for dimension reduction algorithm (UMAP; Materials and Methods). For both the kinases and TF activities, we observed that cancer samples were not clustered by experimental study (Fig 3A, Appendix Fig S9A). The same was also observed using a principal component analysis (PCA; Appendix Fig S9B and C). These results suggest that our normalisation procedures helped to mitigate the technical biases between studies, being likely superimposed by biological variation.

To select highly variable kinases, we first selected one kinase from sets of redundant kinases that have many shared substrates such as AKT1 and AKT2 (Materials and Methods). Of these, we have identified 30 kinases with the largest amount of variation in the predicted kinase activities across all samples (SD > median SD). As expected, these kinases are highly correlated with the lower dimensional UMAP projections (Fig 3B). This set of highly variable kinases contains known cancer drivers and kinases with inhibitors already used in the clinic as cancer treatment, such as BRAF, AKT, MAP2K1, SRC among others. Examining the tumour samples in this two‐dimensional representation indicates that highly co‐regulated kinases from the MAPK signalling pathway tend to be activated or inhibited across the same samples (Fig 3C). CDK1 is known to phosphorylate the casein kinase 2 (CSNK2A1). These kinases together showed opposite correlations with the UMAP projections and, consequently, a distinct regulatory state across the samples (Fig 3B and C).

We obtained pairwise kinase regulatory relationships deposited in the OmniPath database (Türei *et al*, 2016) and correlated their activities (Materials and Methods). We found that kinases that are known to regulate each other were more likely to have correlated patterns of activity across samples (Fig 3D). This was still observed when taking into account cases where the pair of kinases shared some substrates (Appendix Fig S9D; Materials and Methods). Similarly, we would expect that kinases and TFs within the same pathway will tend to have similar patterns of activation across the samples. To investigate this, we modelled the TF activities as a function of the kinase activities using linear regressions (Materials and Methods), identifying 5,712 significant associations at an FDR < 5% (3,130 for FDR < 1%; Fig 3E; Dataset EV4). These associations were enriched in known kinase–TF functional interactions (Appendix Fig S9E and F), including for example the relation between CDK1 activity and the activities of E2F4 and TFDP1 (Jiao *et al*, 2017; Spring *et al*, 2017; Appendix Fig S9F). Altogether, these results corroborate that the variation in activities across the samples is shaped to some extent by the underlying regulatory relationships.

Our analysis can indicate the kinases that are most often misregulated in cancer. For comparison, we estimated kinase activities from phosphoproteomics experiments collected in human cell lines perturbed in a wide range of diverse conditions (e.g. different kinase inhibitors, cell cycle stages and DNA damage; Ochoa *et al*, 2016). Then, we calculated the percentage of tumour samples and perturbations where each kinase showed strong regulation (Materials and Methods). This identifies the kinases that are often regulated in tumour samples and in other non‐cancer‐related conditions. We observed a significant correlation between the percentage of samples where a kinase is regulated in cancer and noncancer conditions (Pearson's *r* = 0.78, *P*‐value = 2.2e‐16), with AKT1 and the cell‐cycle kinases CDK1/2 and AURKB being highly regulated in both sets of conditions (Fig 3F). Kinases deviating from the regression line can be classified as preferentially regulated in the tumours or in the non‐cancer‐related conditions (Materials and Methods). There were a larger number of kinases specifically dysregulated in cancer (e.g. PRKACA, CSNK2A1 and MAPK1) compared with other noncancer conditions (Fig 3F). The kinases MAPKAPK2, RPS6KB1 and RPS6KA3 were more often regulated in the noncancer conditions when compared to their degree of regulation in tumours (Fig 3F). We performed the same analysis but divided by tissue type (Appendix Fig S10A). The number of dysregulated kinases was consistently higher in the tumours than in the noncancer conditions across all tissues (Appendix Fig S10A and B). The number of dysregulated kinases found specifically in tumours of a given tissue correlated with the number of samples potentially indicative of higher statistical power in these tumour types (Appendix Fig S10C). Some kinases (e.g. PRKACA, CSNK2A1 and MAPK1) were found specifically dysregulated across multiple tumour types, but more than half were dysregulated in just one tissue (68%) such as MYLK kinase in stomach cancer and MTOR in kidney cancer (Appendix Fig S10D).

Finally, we clustered the cancer samples into 8 cancer activity subtypes. To do so, we defined each sample by the vector of activities of kinases and TFs and used an approach based on hierarchical clustering to find groups of tumour samples with characteristic patterns of kinase/TF activities (Materials and Methods). We characterised each of the subtypes by performing over‐representation analysis of clinical features and the activities that are most often regulated in each of the clusters. We then used CARNIVAL (Liu *et al*, 2019; Dugourd *et al*, 2021) to investigate the most plausible mechanistic links that could connect the most regulated kinase and TF activities in each cluster (Materials and Methods). We provide an extensive description of these eight activity subtypes in the Appendix. Some of these activity subtypes are enriched in specific tissue or subtypes characterised by other approaches. For example, cluster 1 is enriched in high‐grade serous ovarian cystadenocarcinoma (SOC) with consistent activation of ARID1A. Cluster 5 is enriched in lung carcinoma and breast cancer samples with a general high activity of ZEB2, a promoter of epithelial to mesenchymal transition (EMT), metastasis and resistance in LUAD and breast cancer (Zhang *et al*, 2015; Malvi *et al*, 2019). As an interesting example, Cluster 7 was found to be enriched in CD8‐inflamed tissues with consistent activation of the proinflammatory JUN and NFKB1 TFs likely via the increased activity of PAK1.

### Differential protein activity is associated with changes in patient survival

**Figure 4:**
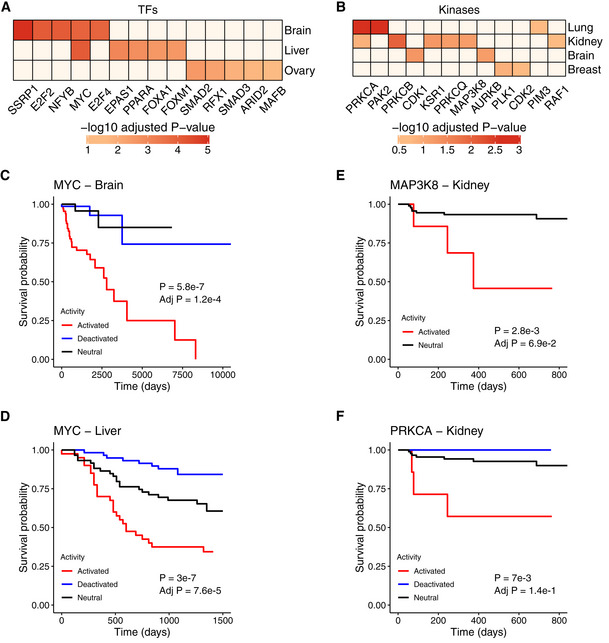
Survival analysis using kinase and TF activities A
Heatmap of log‐rank test adjusted *P*‐values (−log10) comparing Kaplan–Meier survival curves between cancer samples with TF activities classified as inactive, neutral and active (top 5 associations per tissue; FDR < 5%).B
Same as (A) for the kinases (associations with FDR < 20%).C–F
Kaplan–Meier survival plots comparing the survival probabilities (y‐axes) as a function of time in days (x‐axes) for proteins and cancer types shown in (A) and (B). (C) MYC/brain cancer (inactive = 71, neutral = 39, active = 67). (D) MYC/liver cancer (inactive = 58, neutral = 59, active = 40). (E) MAP3K8/renal cancer (neutral = 95, active = 7). (F) PRKCA/renal cancer (inactive = 3, neutral = 92, active = 7). The log‐rank *P*‐values are shown in the plots.

Survival analyses from multiomics datasets have been largely based on mutation, gene or protein expression differences between groups of patients. However, kinase and TF activities should capture the signalling state of the cancer samples and could be also linked to overall patient survival (OS). To explore this, we first performed a log‐rank test to compare the Kaplan–Meier (KM) survival curves between patients with TF and kinase activities classified as inactive, neutral and active (Materials and Methods). We found several TFs and kinases significantly associated with OS in different tumour types (Fig 4A and B; Dataset EV5). For instance, the degree of MYC activity was correlated with OS in brain and liver cancers (Fig 4C and D). In both cases, patients with high MYC activity showed less OS than patients with neutral and inactivated MYC (Fig 4C and D). According to the literature, MYC overexpression is a poor prognosis factor in liver and paediatric brain tumours (Lin *et al*, 2010; Zheng *et al*, 2017). To take into account the effects of possible confounding covariates, we performed a multivariate Cox regression analysis using the protein activity scores as a predictor, while controlling for conventional clinical covariates and the genotype of recurrently mutated genes (Materials and Methods). Reassuringly, our findings with the log‐rank tests were largely recapitulated with the Cox models (brain: hazard ratio (HR) = 1.50 (95% CI 1.27–1.76), adjusted *P*‐value = 2.6e‐5; liver HR = 1.17 (95% CI 1.06–1.31), adjusted *P*‐value = 1e‐2). Interestingly, we also found that high activity of FOXA1 was a good prognostic factor and FOXM1 was a poor prognostic factor in liver cancer (FOXA1: HR = 0.69 (95% CI 0.55–0.85), adjusted *P*‐value = 4.7e‐3; FOXM1: HR = 1.39 (95% CI 1.17–1.66), adjusted *P*‐value = 1.9e‐3; Appendix Fig S11A and B). These two proteins are known for their opposite role in hepatocarcinogenesis. On the one hand, elevated expression of FOXM1 promotes tumour cell proliferation and, on the other hand, FOXA1 inhibits tumour progression by suppression of PIK3R1 expression (Yu *et al*, 2016; He *et al*, 2017).

Regarding the kinases, we found that elevated activity of MAP3K8 and PRKCA was associated with less probability of survival in renal cancer (MAP3K8: HR = 10.5 (95% CI 5.81–19), adjusted *P*‐value = 1.1e‐13; PRKCA: HR = 6.15 (95% CI 4.27–8.84), adjusted *P*‐value = 2.3e‐21; Fig 4E and F). In agreement with these results, the overexpression of both protein kinase C and mitogen‐activated protein kinase 8 has been associated with a higher invasiveness of kidney tumours (Engers *et al*, 2000; Li & Gobe, 2006; Su *et al*, 2015; Liu *et al*, 2016). We were able to replicate the survival curve for PRKCA (*P*‐value = 7.5e‐3) on a separate cohort of cancer patients from TCGA using RPPA phosphorylation measures (Appendix Fig S11C). Lastly, overactive AURKB was correlated with a lower survival rate in breast cancer (HR = 27.2 (95% CI 11.2–66.2), adjusted *P*‐value = 5.1e‐12; Appendix Fig S11D), as previously found at the gene expression level (Huang *et al*, 2019). Altogether, these results indicate that the inference of kinase and TF activities can be a relevant prognostic tool in cancer studies.

## Discussion

Kinases and TFs are important mediators of cell signalling regulation and sensitivity to anticancer drugs. Here, we have compiled multiomics datasets made available by the TCGA and CPTAC consortia and cell line studies and we were able to estimate the activities of 218 kinases and 292 TFs in over 1,000 primary tumours and cancer cell lines. Based on these, we found that kinase activities appear to be primarily regulated by phosphorylation level with fewer cases of significant correlation with the predicted kinase protein abundance levels. Contrary to this, the predicted TF activity is primarily correlated with the mRNA/protein level of the TF itself with a smaller proportion of TFs with significant correlations with the phosphorylation state. This difference in regulation is not simply due to lack of detection of phosphosites as TFs have a median value of six phosphosites detected compared with nine for kinases. A larger fraction of the TF activities is correlated with their mRNA levels than the protein abundance. This result is nonintuitive since the protein abundance should be a better proxy for activity. It is possible that this is due to the fact that TF activities are derived directly from the same mRNA datasets while there will be some degree of technical variation due to sample preparation and analysis when compared to the protein dataset.

Intuitively, the activity of a given protein (i.e. kinases and TFs) might be positively or negatively affected by mutations in the same protein or in other proteins it interacts with throughout the signalling networks. Our genetic analysis identified associations between mutated genes and the activities of kinases and TFs, which were significantly enriched for known protein–protein interactions. Moreover, we found that the activities of the transcription factors TP53 and SREBF2 were correlated with their mutational status, as described previously (Garcia‐Alonso *et al*, 2018). Nevertheless, we did not observe a general correlation between deleterious mutations within a kinase/TF and its activity, including expected associations such as the activating BRAF V600E mutation and the activities of BRAF itself or other members of the MAPK pathway. These results were not due to issues linked with purity or immune infiltration as the same was observed in cell lines. Nevertheless, there might be issues in linking mutations with signalling differences due to single‐cell‐level variances that cannot be systematically profiled in bulk (Lun & Bodenmiller, 2020). In future, single‐cell multiomics profiling may allow us to consider intratumour genetic heterogeneities. We speculate that these observations are more likely explained by adaptation mechanisms that are prevalent in signalling pathways (Lito *et al*, 2013). Systematic studies comparing acute inhibition with cells adapted to loss‐of‐function mutations have not yet been done. However, systematic phosphoproteomic studies of kinase gene deletions strains showed that the regulated phosphosites in the mutant strains had very little overlap with kinase interactors (Bodenmiller *et al*, 2010). This result in yeast supports the observations made here with tumour samples. This emphasises the difficulty in interpreting the impact of mutations on signalling networks and the importance of studying directly the dysregulation of signalling in cancer (Yaffe, 2019).

The lack of correlation between loss‐of‐function mutations and protein activities may be due to sources of genetic and nongenetic (e.g. epigenetic factors, stochasticity of biomolecules and environmental stimuli) heterogeneities between individual cancer cells that are unable to be uncovered in bulk. In future, single‐cell multiomics profiling may help to study signalling networks cell‐by‐cell and help to uncover the signalling consequences of intratumoral genetic and nongenetic factors (Lun & Bodenmiller, 2020).

Known kinase regulatory pairs have strong patterns of co‐regulation across the compiled dataset. These results suggest that the kinase activity estimates are meaningful and that the variation in kinase activities along cancer samples is likely driven by biological factors. We showed in a previous work that these co‐regulation signals can be used to predict kinase regulatory networks (Invergo *et al*, 2020). Similarly, we found many significant associations between the activities of kinases and TFs, which were significantly enriched in known functional interactions. This indicates that this compendium of protein activities may be useful in the future development of methods to reconstruct the signalling networks. Nevertheless, even the strongest correlations were modest in aggregate: well‐studied kinase–kinase regulatory pairs showed a median correlation of their predicted activities of 0.25 (Fig 3D). Our prior knowledge about kinase co‐regulation is currently limited at multiple levels, including yet‐to‐be‐found regulatory relationships and the extent that these regulatory relationships depend on the tissue of origin or other factors. We speculate that these and other confounding factors could explain the weak kinase–kinase correlations.

By comparing the kinases most often differentially regulated across tumours and after more acute perturbations, we have shown that most often regulated kinases are the same in both contexts. These include kinases such as CDK1, AKT1 and AURKB. The most plausible explanation for this would be that kinases that are often regulated in acute perturbations are directly linked to the regulation of growth and cell‐cycle and other critical processes needed to be regulated in cancer cells. Alternatively, we have shown that these highly regulated kinases occur in very central positions in the signalling network (Ochoa *et al*, 2016) and that it is possible that some degree of regulation of these kinases is almost unavoidable. However, this comparison allowed us to identify kinases, which show higher differential regulation in tumours than acute perturbation such as PRKACA, MAPK1 and MAPK3.

Finally, we show how the estimated protein activity can be linked to differences in patient survival. Given that the activities of kinases and TFs can often be estimated via antibodies targeting regulatory phosphosites, it may be possible to develop biomarkers based on these findings. In addition, kinases are very tractable drug targets with multiple kinase drugs already used to treat cancer patients. While further studies in cell‐based and animal models will be required to evaluate the significance of the findings presented here, this work provides kinase and TF activities linked to specific tumour types and mutational contexts that could be pursued for potential treatment.

## Materials and Methods

### Data collection

#### Proteomics and phosphoproteomics

The mass spectrometry (MS)‐based protein and phosphosite quantifications (absolute [phospho]peptide intensities and ratios relative to controls) for the cancer samples of brain (Petralia *et al*, 2020), breast (Mertins *et al*, 2016), colorectal (Zhang *et al*, 2014), kidney (Clark *et al*, 2019), liver (Gao *et al*, 2019), lung (Gillette *et al*, 2020), ovarian (Zhang *et al*, 2016), stomach (Mun *et al*, 2019) and uterus (Dou *et al*, 2020) were downloaded from the CPTAC data portal (proteomics.cancer.gov/data-portal). For the colon cancer samples (Vasaikar *et al*, 2019), we downloaded the data from the linkedomics database (linkedomics.org/login.php). The same data for the cancer cell lines of breast and colorectal tumours were downloaded from the respective publications (Lawrence *et al*, 2015; Lapek *et al*, 2017; Roumeliotis *et al*, 2017). The proteins and phosphosites were identified using gene symbols, in a process described by the common data analysis pipeline (CDAP) from CPTAC. Additionally, we downloaded normalised RPPA protein and phosphorylation quantification data (183 features across 7,694 samples from 31 TCGA tumours) from the TCPA (Li *et al*, 2013) database.

#### Transcriptomics

The RNA‐seq data were obtained in the format of read counts and Fragments Per Kilobase of transcript per Million mapped reads (FPKM). The data for the tumour tissues of breast (Mertins *et al*, 2016), colorectal (Zhang *et al*, 2014), kidney (Clark *et al*, 2019), lung (Gillette *et al*, 2020), ovarian (Zhang *et al*, 2016) and uterus (Dou *et al*, 2020) were downloaded from the GDC portal (portal.gdc.cancer.gov/). The data for the brain cancer were compiled from the paediatric cBioPortal (pedcbioportal.kidsfirstdrc.org/); for the liver (Gao *et al*, 2019) from NODE (www.biosino.org/node/; accession ID: OEP000321); for the stomach (Mun *et al*, 2019) from GEO (ncbi.nlm.nih.gov/geo/; accession ID: GSE122401); and for the colon cancer (Vasaikar *et al*, 2019) from the authors. The cancer cell lines (Lawrence *et al*, 2015; Lapek *et al*, 2017; Roumeliotis *et al*, 2017) data were downloaded from the CCLE data portal (portals.broadinstitute.org/ccle/data).

#### Genomics—somatic mutations

The whole‐genome sequencing (WGS)‐derived somatic mutations for the brain cancer samples (Petralia *et al*, 2020) were downloaded from the paediatric cBioPortal (pedcbioportal.kidsfirstdrc.org/) in Mutation Annotation Format (MAF) files. For the breast (Mertins *et al*, 2016), colorectal (Zhang *et al*, 2014) and ovarian (Zhang *et al*, 2016) cancers, the whole‐exome sequencing (WES)‐derived MAF files were downloaded from the cBioPortal (cbioportal.org). The MAF file for the colon cancer samples (Vasaikar *et al*, 2019) was downloaded from the linkedomics database (linkedomics.org/login.php). Regarding the kidney (Clark *et al*, 2019), lung (Gillette *et al*, 2020) and uterus (Dou *et al*, 2020) cancers, we downloaded the MuTect2‐called and VEP‐annotated VCF files from the GDC data portal (portal.gdc.cancer.gov/). For the liver (Gao *et al*, 2019) and stomach cancers (Mun *et al*, 2019), we obtained the somatic mutations from the publication and authors, respectively. The mutation data for the colorectal and breast cancer cell lines (Lawrence *et al*, 2015; Lapek *et al*, 2017; Roumeliotis *et al*, 2017) were obtained from the DepMap portal (depmap.org/portal/).

#### Genomics—somatic copy number alterations

The somatic copy number variation (CNV) data were downloaded as discretised GISTIC2 scores (Beroukhim *et al*, 2007; Mermel *et al*, 2011) and segment‐level log2 ratios between the tumour and normal samples. The GISTIC2 scores can be −2 (strong copy number loss, likely a homozygous deletion), −1 (shallow deletion, likely a heterozygous deletion), 0 (diploid), 1 (low‐level gain of copy number, generally broad amplifications) and 2 (high‐level increase in copy number, often focal amplifications). The GISTIC2 scores for the tumour samples of breast (Mertins *et al*, 2016), colorectal (Zhang *et al*, 2014) and ovarian (Zhang *et al*, 2016) and for the cancer cell lines (Lawrence *et al*, 2015; Lapek *et al*, 2017; Roumeliotis *et al*, 2017) were downloaded from the cBioPortal (cbioportal.org). The same data for the brain cancer samples (Petralia *et al*, 2020) were downloaded from the paediatric cBioPortal (pedcbioportal.kidsfirstdrc.org/); for the colon samples (Vasaikar *et al*, 2019) from linkedomics (linkedomics.org/login.php); and for the liver samples (Gao *et al*, 2019) from NODE (biosino.org/node/index; accession ID: OEP000321). The segment‐level log2 ratios for the kidney (Clark *et al*, 2019) and uterus (Dou *et al*, 2020) cancer samples were provided by the authors of the respective publications.

#### Clinical data

The metadata and clinical information from the patients of breast (Mertins *et al*, 2016), colorectal (Zhang *et al*, 2014) and ovarian (Zhang *et al*, 2016) cancers was obtained from the CPTAC (proteomics.cancer.gov/data-portal) and the cBioPortal (cbioportal.org) databases. The survival data for these patients were collected from (Liu *et al*, 2018), whereas the cancer subtypes were obtained from the respective publications and also using the *PanCancerAtlas_subtypes* function from the *TCGAbiolinks* R package (Colaprico *et al*, 2016). For the brain (Petralia *et al*, 2020), kidney (Clark *et al*, 2019), liver (Gao *et al*, 2019), lung (Gillette *et al*, 2020), stomach (Mun *et al*, 2019) and uterus (Dou *et al*, 2020) cancers, we downloaded the clinical information from the CPTAC portal and from the respective publications. For the colon cancer samples, we downloaded the data from the linkedomics database (inkedomics.org/login.php). The clinical data from the cancer cell lines donors (Lawrence *et al*, 2015; Lapek *et al*, 2017; Roumeliotis *et al*, 2017) were obtained from the CCLE data portal (portals.broadinstitute.org/ccle/data). Altogether, we collected the following information about the cancer patients: age, gender, ethnicity, race, height, weight, cancer histological type and subtype, tumour stage, overall survival and survival time in days.

### Data preprocessing and normalisation

#### Proteomics

The label‐free protein quantifications (precursor areas) for the colorectal tumours (Zhang *et al*, 2014) and the tandem mass tag (TMT) protein intensities for the breast and colorectal cancer cell lines (Lawrence *et al*, 2015; Lapek *et al*, 2017; Roumeliotis *et al*, 2017) were pre‐processed and transformed to log2 fold‐changes as described previously (Sousa *et al*, 2019). For the brain (Petralia *et al*, 2020), lung (Gillette *et al*, 2020) and stomach (Mun *et al*, 2019) cancers, the sample replicates were combined by averaging the log2 fold‐change values of each protein. After that, we removed six outlier samples from colorectal cancer with an absolute median log2 fold‐change distribution higher than 1 (2‐fold). Altogether, we assembled a matrix with 14,742 proteins and 1,266 samples (1,170 cancer samples and 96 cell lines) belonging to nine different tissues. This matrix contained 9,941,918 protein measures (8,721,454 missing values) and 5,052 proteins quantified in at least 80% of the samples.

#### Phosphoproteomics

The phosphorylation measures were acquired at the phosphosite level. Each phosphosite is identified by a given protein, position and residue. The phosphosites from the different datasets were harmonised against a common reference by only keeping the phosphorylation sites that mapped correctly to the Ensembl human proteins (GRCh37—release 98). As the phosphorylation sites were annotated at the gene symbol level (see data collection above), we mapped the phosphosites to the protein sequences using the canonical transcripts from UniProt (github.com/mskcc/vcf2maf/blob/main/data/isoform_overrides_uniprot). Duplicated phosphosites, arising from multiple phosphopeptide intensities mapping to the same phosphosite, were reduced to a single phosphosite if the log2 fold‐change values were the same across all samples from the respective experimental study. All duplicated phosphosites were discarded otherwise. For the colorectal cancer cell lines (Roumeliotis *et al*, 2017), the relative TMT intensities (obtained by dividing the TMT intensities per the mean TMT intensity for each protein) were divided by 100 and transformed to log2. For the brain (Petralia *et al*, 2020), breast (Mertins *et al*, 2016), lung (Gillette *et al*, 2020) and stomach (Mun *et al*, 2019) cancers, the sample replicates were combined by averaging the log2 fold‐change values of each phosphosite. We removed 52 outlier samples with an absolute median log2 fold‐change distribution higher than 1 (2‐fold). Then, the log2 fold‐change distributions across samples were quantile normalised in order to ensure comparable distributions, using the *normalizeQuantiles* function from the *limma* R package (Ritchie *et al*, 2015). To detect phosphorylation changes that are independent of the protein abundance, we regressed out the protein levels from the respective phosphosites using a multiple linear regression model. The phosphosite log2 fold‐changes were set as the dependent variables while the protein log2 fold‐changes, age and gender were set as the independent variables. The residuals from the linear model were the phosphorylation changes not driven by the protein abundance or other confounding effects (age and gender). The final phosphoproteomic matrix contained 86,044 phosphosites across 980 samples (930 cancer samples and 50 cell lines) from nine different tissues. Due to the sparseness of the phosphorylation data (7,280,101 measures and 77,043,019 missing values), only 256 phosphosites were quantified in at least 80% of the samples (2,438 in 50%). For the downstream analyses, we only considered the phosphosites (69,599) that were quantified from phosphopeptides phosphorylated at single positions.

#### Transcriptomics

The RNA‐seq data (FPKMs and read counts) downloaded from the GDC and GEO websites (see data collection above) were converted to tabular formats using in‐house R scripts. For the liver (Gao *et al*, 2019) and stomach (Mun *et al*, 2019) cancer samples, we calculated FPKM expression values from the RSEM (Li & Dewey, 2011) expected counts using the *rpkm* function from the *edgeR* R package (Robinson *et al*, 2010). We obtained the gene lengths by calculating the size (in base pairs) of the merged exons of each gene, using the *gtftools* python script (preprint: Li, 2018; genemine.org/gtftools.php) and the GENCODE v19 human gene annotation (gencodegenes.org). After selecting the protein‐coding genes, as described in the GENCODE v19 annotation, we removed the genes without expression (FPKM > 0) in at least 50% of the samples of the respective dataset. The FPKMs of each gene were subsequently log2 transformed (adding a pseudocount of 1 to avoid taking the log of 0) and converted to log2 fold‐changes by subtracting the log2 median FPKM across samples. The log2 fold‐changes were calculated for each dataset separately. The final gene expression matrix contained 17,056 genes across 1,187 samples and 9 tissues (1,110 cancer samples and 77 cell lines). 14,966 genes were expressed in at least 80% of the samples.

#### Genomics—somatic mutations

We processed the VEP‐annotated VCF files from the kidney (Clark *et al*, 2019), lung (Gillette *et al*, 2020) and uterus (Dou *et al*, 2020) cancer samples using the *bcftools split‐vep* plugin (samtools.github.io/bcftools/howtos/plugin.split-vep.html) with the following parameters: *‐f '%CHROM\t%POS\t%REF\t%ALT\t%QUAL\t%FILTER\t%CSQ\n' ‐d ‐A tab*. Only the mutations passing all quality filters (FILTER == “PASS”) were selected for downstream analyses. The mutations from these samples were then collected in a single text file using in‐house bash scripts. In all datasets, we selected the mutations that were annotated using the canonical UniProt transcripts (github.com/mskcc/vcf2maf/blob/main/data/isoform_overrides_uniprot) and classified as frameshift and in‐frame insertions/deletions (Indels), missense, nonsense, stop codon loss (readthrough mutations) and splice site. All mutations (except splice site) were standardised against the Ensembl human proteins (GRCh37—release 98) by filtering out those mutations whose reference (wild type) residues did not match the protein sequences in the mutation positions. The reference/mutated residues and protein position of the mutations were extracted from the HGVSp codes. In total, we collected 284,882 mutations in 17,305 protein‐coding genes, across 1,168 samples (1,079 tumours and 89 cell lines) from nine different tissues.

#### Genomics—somatic copy number alterations

GISTIC2 (version 2.0.23) was used to process the segment‐level log2 ratios for the kidney (Clark *et al*, 2019) and uterus (Dou *et al*, 2020) cancer samples and define the gain/loss events of each gene (see data collection above), using the default parameter settings (*‐genegistic* and *‐savegene* parameters were both set to 1). After obtaining the discretised GISTIC2 CNV scores for each dataset, only protein‐coding genes were selected as described in the GENCODE v19 human gene annotation. The final CNV matrix contained 16,520 genes across 1,025 samples and seven tissues (947 tumours and 78 cell lines).

#### Normalisation of gene and protein expression data

The confounding factor related to the experimental batch (e.g. CPTAC‐breast and CPTAC‐brain) was removed using a linear regression model. This model was implemented with the mRNA expression or protein abundance of a given gene as a dependent variable and the experimental batch as independent variable. The residuals from the linear model were the protein or mRNA variation not driven by the technical differences between cancer datasets.

### NCI60 and CRC65 cell lines—data collection and pre‐processing

The proteomics and phosphoproteomics data for the NCI60 and CRC65 cancer cell lines (trypsin‐digested version) were downloaded from (Frejno *et al*, 2020). The phosphorylation residues were obtained by mapping the position of the modifications to the UniProtKB/Swiss‐Prot canonical and alternative (isoforms) protein sequences (release 2020_02; uniprot.org/downloads). Only the phosphosites mapping to serine, threonine and tyrosine residues were selected. The log10 transformed phosphorylation and protein absolute abundances (iBAQ) were set to the original values using powers of 10 (10^*abundance*). Phospho(peptide) abundances mapping to the same phosphosite or protein were averaged per sample. The absolute abundances were converted to relative values (fold‐changes) by calculating the log2 ratio of the abundances over the median abundance across cell lines. This process was performed for both cancer cell line sets. To detect net phosphorylation changes, we regressed out the protein levels from the respective phosphosites using the residuals of a linear regression model (y ∼ x) where the phosphosites were set as dependent variables (y) and the proteins as independent variables (x). In total, we assembled 11,940 proteins and 45,557 phosphosites across 125 cell lines (60 from NCI60 and 65 from CRC65).

The gene expression data were downloaded in the format of FPKMs (discover.nci.nih.gov/cellminer/) and Transcripts Per Million (TPMs; depmap.org/portal/), for the NCI60 and CRC65 cell lines, respectively. Both gene expression measures were log2 transformed and converted to fold‐changes by subtracting the log2 median FPKM/TPM across cell lines. In total, we calculated the log2 fold‐changes in 18,291 genes across 95 cell lines (60 and 35 from the NCI60 and CRC65 sets, respectively). The whole‐genome mutation data were downloaded from the CellMiner (discover.nci.nih.gov/cellminer/) and the DepMap databases (depmap.org/portal/) for the NCI60 and CRC65 cancer cell lines, respectively. Across the NCI60 cell lines, we selected those mutations where more than 50% of the respective reads contained the alternative allele. In both datasets, we selected the mutations annotated as silent, missense, nonsense, stop codon loss, frameshift and in‐frame Indels. The protein position of the mutations and respective reference/mutated residues was obtained from the HGVSp codes. Altogether, we collected 585,904 mutations in 17,259 genes along 96 cell lines (60 from NCI60 and 36 from CRC65).

### Inference of kinase and TF activities

Kinase and TF activities were estimated using known kinase and TF regulatory targets. The kinase–substrate relationships were obtained from (i) ProtMapper (preprint: Bachman *et al*, 2019), a literature‐based resource of kinase substrates annotated at the phosphosite level. The resource contains phosphorylation sites aggregated from five databases (BEL Large Corpus, NCI‐PID, PhosphoSitePlus, Reactome and SIGNOR) and three text‐mining tools (REACH, RLIMS‐P and Sparser) and (ii) a collection of phosphosites derived from *in vivo* (Hijazi *et al*, 2020) and *in vitro* (Sugiyama *et al*, 2019) experiments. Only the phosphorylation sites correctly mapped to the Ensembl human proteins (GRCh37—release 98) were considered for the subsequent analyses. In total, we collected the phosphorylation targets of 573 kinases. The transcriptional targets of the TFs were compiled from the DoRothEA R package (v1.2.0), using only interactions annotated with confidence A, B and C. The kinase activities were inferred using a one‐sample *z*‐test, which was shown to perform well (Hernandez‐Armenta *et al*, 2017). The activity of a given kinase in a given sample was estimated as follows:

(1) $$z=\frac{x-\mu }{\sigma /\sqrt{N}}$$

where *z* corresponds to the *z*‐score, *x* the average log2 fold‐change in the kinase substrates, μ the average log2 fold‐change in all phosphosites measured in the sample (background), √*N* the square root of the number of kinase substrates (*N*) and σ the standard deviation of the background. Then, the *z*‐score was used to calculate a two‐tailed *P*‐value using the *pnorm* R function [2 × pnorm(−abs(*z*))], which was further log10 transformed and signed based on the position of the *z*‐score in the standard normal distribution. If the *z*‐score was in the right part of the distribution, that is positive, the kinase substrates showed an increase in phosphorylation in comparison with the sample background. Thus, the activity of that kinase was also expected to be increased (positive) in that sample, and vice versa. This process was repeated for all kinases across all samples. For the downstream analyses, we selected the kinase–substrate interactions from the databases and text‐mining resources and the kinase activities quantified with three or more substrates, resulting in 218 kinases with activity estimates in an average of 437 cancer samples (of a total of 980 samples).

We also estimated kinase activities based on the phosphorylation changes in phosphosites mapping to the kinases. We selected the phosphosites with known regulatory status in PhosphositePlus or with unknown status but with a functional score higher than 0.4 (1,534 of 4,247 kinase‐mapping phosphosites). The functional score was calculated using the *FunscoR* R package (evocellnet.github.io/funscoR/). The scores range from 0 to 1 and reflect the functional consequence of the phosphosites (Ochoa *et al*, 2020). The kinase activity inference method was the one‐sample *z*‐test as described previously. TF activities were estimated using the VIPER algorithm (Alvarez *et al*, 2016), using log2FC as gene‐level statistics (see Data preprocessing and normalisation—transcriptomic). VIPER was run with a minimum limit of regulon size of 5, and using all provided gene‐level statistics as a background (eset.filter = FALSE). VIPER returned a normalised enrichment score for 292 TFs across 1,187 cancer samples.

### Benchmark of the kinase targets

We validated the kinase activities calculated from the different sources of kinase targets (database, text‐mining, *in vivo* and *in vitro*) using a MS‐based phosphoproteomic dataset reporting the relative phosphorylation changes in 52,814 phosphosites in 103 human perturbation‐dependent conditions (Ochoa *et al*, 2016; Hernandez‐Armenta *et al*, 2017). These data include a gold standard dataset composed of 184 kinase–condition pairs where kinase regulation is expected to occur. The *z*‐test‐based absolute kinase activity scores estimated from the different kinase substrate sources were used as classifiers of kinase regulation. Given the imbalance between the positive (gold standard) and negative (kinase–condition pairs with unknown regulation) classes, we generated 100 random sets of negative cases with the size of the positive set. The predictive skill of each classifier was evaluated by the mean area under the receiver operating characteristic curves (AUROCs). As a control, we replicated the 100 random sets of negative and positive pairs (53 pairs each) along the different lists of kinase–substrates. The ROC curves and corresponding AUCs were calculated using the *prediction* and *performance* functions from the *ROCR* R package.

### Impact of highly recurrent cancer mutations in the MAPK/ERK signalling transduction pathway

We assessed the impact of the recurrent cancer mutations BRAF^V600E^, KRAS^G12C^ and KRAS^G12D^ in the protein activities of BRAF, MAPK3, MAPK1, MAP2K1 and MAP2K2 by fitting the following multiple linear regression model:

(2) $$\text{KinAct}=\beta_{0}+\beta_{1}\;{\text{BRAF}}^{V600E}+\beta_{2}\;{\text{KRAS}}^{G1\text{2C}}+\beta_{3}\;{\text{KRAS}}^{G1\text{2D}}+ɛ$$

where KinAct represents the activity of the kinases from the MAPK/ERK signalling pathway, **β**_0_ the intercept, **β**_1_, **β**_2_ and **β**_3_ the regression coefficients for the recurrent mutations and ɛ the noise term. This model was fitted for each kinase individually. Additionally, we evaluated the impact of the BRAF^V600E^ mutation in the activities of all kinases inferred in this study by fitting a similar linear regression model with only one nonintercept coefficient for BRAF^V600E^.

The *P*‐values from the regression coefficients were calculated using the *t*‐statistic over a Student's *t*‐distribution and adjusted for false discovery rate (FDR) using the Benjamini–Hochberg method. The linear models and respective statistics were calculated using the *lm* and *p.adjust* R functions.

### Differential expression of phosphatases in cancer samples with BRAF^V600E^ mutation

We obtained 281 genes with phosphatase activity from the Molecular Signatures Database (www.gsea-msigdb.org) using the gene ontology molecular function term GOMF_PHOSPHATASE_ACTIVITY. Out of 19 cancer samples with the BRAF^V600E^ mutation and profiled with BRAF kinase activities, we selected the 14 samples with activities above the noise level (absolute activity value > 0.15) and with gene expression data. Then, we clustered the samples as high (six samples) and low (eight samples) BRAF activity according to the respective kinase activity sign. The differential gene expression analysis was then performed for 237 phosphatases with gene expression values across all cancer samples. The log2 fold‐changes between high and low BRAF activity samples were calculated from the FPKM expression values as log2(mean(low BRAF samples) / mean(high BRAF samples)). The statistical significance of the gene expression differences was assessed using a Wilcoxon rank‐sum test. The respective *P*‐values were adjusted for false discovery rate (FDR) using the Benjamini–Hochberg method. The Wilcoxon rank‐sum test and respective statistics were calculated using the *wilcox.test* and *p.adjust* R functions.

### Genetic associations with the kinase and TF activities

The effects of mutations on the kinase and TF activities were assessed by associating the activity of a given protein with the mutational status of the same protein or other proteins it might interact throughout the cellular regulatory networks. First, we built a binary mutation matrix M where the index M_ij_ corresponds to 1 if the sample i has a mutation in gene j and 0 otherwise. To do that, we selected the mutations classified as frameshift and in‐frame Indels, missense, nonsense and stop codon loss. Given the proteins X and Y, the association between the activity of Y (Y_act_) and the mutational status of X (X_mut_) was assessed across samples by fitting a linear model that took into account possible confounding effects:

(3) $$Y_{\mathrm{act}}=\beta_{0}+\beta_{1}\;\text{Study}+\beta_{2}\;X_{\mathrm{mut}}+ɛ$$

where Y_act_ represents the activity of protein Y, **β**_0_ the intercept, **β**_1_ the regression coefficient for the covariate experimental study, **β**_2_ the regression coefficient for the mutational status of X and ɛ the noise term. This model was applied to assess the effect of X_mut_ on the activity of the same protein (X_act_ ∼ X_mut_) and on the activity of other proteins (Y_act_ ∼ X_mut_). The *P*‐values from the coefficients of X_mut_ (**β**_2_) were calculated using the *t*‐statistic over a Student's *t*‐distribution and adjusted for false discovery rate (FDR) using the Benjamini–Hochberg method. The linear models and respective statistics were calculated using the *lm* and *p.adjust* R functions.

The associations were performed with the genes mutated in more than 20 samples and with the protein activities estimated in at least 10 samples. An association between a pair Y_act_ ∼ X_mut_ or X_act_ ∼ X_mut_ was performed if X_mut_ was mutated in at least five of all the samples in the pair. Regarding the Y_act_ ∼ X_mut_ associations, we tested 520,938 pairs between 208 kinases and 3,590 genes and 1,048,216 pairs between 292 TFs and 3,590 genes. In relation to the X_act_ ∼ X_mut_ associations, we tested 40 pairs and 64 pairs with the kinases and TFs, respectively.

### Projection of the protein activities in low‐dimensional spaces

We reduced the dimensionality of the kinase and TF activity matrices using the PCA and UMAP methods. Given the sparseness of the kinase activity matrix, we imputed the missing values using the *missForest* function from the *missForest* R package. Prior to that, we selected the kinases (columns) with activity measures in at least 60% of the samples and the samples (rows) with measures in at least 80% of the kinases. The imputed kinase activity matrix contained 90 kinases across 727 samples. The PCA analysis was performed using the *prcomp* R function (scale. = T, centre = T) and the UMAP analysis using the *umap* function from the *umap* R package (with default parameters).

When correlating the kinase activities with the UMAP projections (Pearson correlation coefficient), we excluded redundant kinases based on the degree of shared substrates. We first performed a hierarchical clustering analysis (*hclust* R function, agglomeration method = “complete”) using the Jaccard Index (JI) of shared substrates between kinases as distance measure (1‐JI). Then, the kinase dendrogram was cut at a specific level (height = 0.85) to identify clusters of non‐redundant kinases. We only kept one kinase per cluster (with the largest amount of substrates), reducing the number of kinases from 304 to 208.

### Correlation of kinase pairs

We obtained kinase–kinase regulation pairs from the OmniPath database (omnipathdb.org/interactions). We selected the interactions reported as directed, activating (stimulating relationships) and consensual along the resources (databases). Then, we correlated the activity of the kinase–kinase pairs along the samples using the Spearman's rank correlation coefficient. The kinase pairs were stratified by the number of databases in which the interaction was found as a way of ascertaining the relevance of the interactions.

Strong correlations might be due to the amount of shared substrates between kinase pairs, and not because of co‐regulation events. To control for this technical limitation, we repeated the correlation analysis using a set of nonredundant kinases. This set was obtained by performing a hierarchical clustering analysis (*hclust* R function, agglomeration method = “complete”) using the degree of shared substrates between kinases as distance measure (1—Jaccard Index of shared substrates). The dendrogram tree was cut at a height cut‐off of 0.8. Just one kinase was kept per cluster (with the highest number of substrates). Using this approach, we reduced the number of kinases from 304 to 231.

### Associations between the activities of kinases and transcription factors

For a given protein pair K and T, where K is a kinase and T is a transcription factor, we tested whether the changes in the activity of kinase K are linearly associated with changes in the activity of the transcription factor T. To do that, we fitted a linear model to predict the activity of transcription factor T (T_act_) using the activity of kinase K (K_act_), while adjusting for possible confounding effects:

(4) $$T_{\mathrm{act}}=\beta_{0}+\beta_{1}\;\text{Study}+\beta_{2}\;K_{\mathrm{act}}+ɛ$$

where T_act_ represents the activity of the transcription factor T, **β**_0_ the intercept, **β**_1_ the regression coefficient for the covariate experimental study, **β**_2_ the regression coefficient for the activity of kinase K and ɛ the noise term. The *P*‐values from the coefficients of K_act_ (**β**_2_) were calculated using the *t*‐statistic over a Student's *t*‐distribution and adjusted for false discovery rate (FDR) using the Benjamini–Hochberg method. The linear models and respective statistics were calculated using the *lm* and *p.adjust* R functions. Using this model, we tested 26,280 kinase–TF associations between 90 kinases and 292 TFs.

### Enrichment of the protein association pairs in the STRING network

The genetic and the kinase–TF associations were tested for enrichment in the STRING protein–protein interactions network using Fisher's exact tests (*fisher.test* R function, alternative = “greater”). The human network (version 11.0) was downloaded from the STRING database (string-db.org) as a list of protein–protein interactions with the corresponding combined scores. The scores range from 150 to 999 and represent the confidence of the respective interactions. We filtered the network using a minimum score of 850 to select the most confident protein–protein interactions. Fisher's exact tests were performed by overlapping the protein association pairs with the STRING network across increasing ‐log10 adjusted *P*‐values. The backgrounds corresponded to all the protein association pairs linearly modelled.

### Kinase activity changes between tumours and perturbations

To study the differences of kinase signalling between tumours and perturbation‐dependent conditions, we estimated the activity of kinases across an extended panel of perturbations with phosphoproteomic measurements (Ochoa *et al*, 2016). This dataset is composed of 76,379 phosphosites across 439 perturbations. Next, we calculated the percentage of tumour samples and perturbations each kinase was regulated in, using an absolute kinase activity cut‐off of 1.75 as previously used. We kept the kinases regulated in at least 1 tumour or perturbation. In order to find the kinases preferentially regulated in the tumours and in the perturbations—tumour or perturbation‐specific kinases—we fitted a linear model between the percentage of kinase regulation in the tumours and in the perturbations, as independent and dependent variables, respectively. The most deviating kinases from the regression line were considered to be differentially regulated. These kinases were found by converting the residuals of the linear model to *z*‐scores: while the kinases with a residual *z*‐score > 2 were classified as tumour‐specific, the kinases with a residual *z*‐score < −2 were classified as perturbation‐specific. This process was performed across all cancer samples and by tissue type. The linear models and respective residuals were calculated using the *lm* and *residuals* R functions. The residuals were standardised to *z*‐scores using the *scale* R function.

### Survival analysis

In order to construct Kaplan–Meier (KM) survival curves, cancer samples were stratified based on their TF and kinase activity scores (AS). For each kinase and TF, we classified the samples as: inactive if AS < −1.75; active if AS > 1.75; neutral if −1.75 < AS < 1.75. The 1.75 activity cut‐off was chosen based on a previous publication (Ochoa *et al*, 2016). We estimated the KM survival curves by protein and tissue. We tested whether the differences on the activities of a given TF on a given tissue were associated with the probability of survival across time if more than 10 deaths occurred and more than 10 samples were classified as active and inactive. Given the lower number of activation/inactivation events for the kinases, we tested the kinase‐tissue pairs with more than 5 samples classified as active or inactive and with more than 5 deaths. These filters resulted in 1,025 tests for the TFs (274 TFs and five tissues) and 195 tests for the kinases (81 kinases and 7 tissues). The survival distributions of the cancer sample groups were compared using log‐rank tests with the *survdiff* function from the *survival* R package. The *P*‐values were adjusted for FDR using the Benjamini–Hochberg (BH) procedure (*p.adjust* R function). The KM curves were plotted using the *ggsurvplot* function from the *survminer* R package.

To account for confounding covariates, we performed a multivariate statistical analysis using Cox proportional hazards regression models. The hazard function was fitted using the protein activity scores as a continuous predictor, adjusted for age, gender and the genotype (1 if mutated and 0 otherwise) of 28 recurrently mutated genes in our atlas (at least 100 mutations). Such models were applied to the protein–tissue pairs described previously. We extracted the hazard ratios of the protein activity coefficients and corresponding 95% confidence intervals and *P*‐values from the Cox models. The BH‐corrected *P*‐values were calculated using the *p.adjust* R function. The Cox regression models were fitted using the *coxph* function from the *survival* R package.

### Stratification of tumour samples

To cluster the samples based on TF and kinase activities, a cross‐correlation matrix of samples was created based on the Spearman correlation coefficient of a combination of 292 TFs and 90 kinase activity scores in each sample.

Then, Euclidean distance was computed between each sample based on their correlation profiles. A hierarchical clustering tree was created using the complete method over the euclidean distance matrix. The hierarchical tree was then cut at the minimum tree height to obtain eight groups. The number of clusters was decided based on visual inspection of the within‐cluster sum of square plot, silhouette profile and of a heatmap of the cross‐correlation matrix.

### Cluster‐level activity

Each kinase and TF was then summarised into a single cluster‐level activity score. The cluster‐level activity scores were calculated as the median activity score in each cluster divided by the standard deviation of the corresponding activities in each cluster.

### Clinical feature over‐representation

Clinical annotations for body mass index (BMI) and age were binarised (BMI cut‐off = 37 into “big” and “smol” levels, age cut‐off = 50 into “young” and “venerable” levels). Clinical annotation levels that were populated for less than five patients in the cohort were excluded. Each clinical annotation level was then tested for over‐representation in each cluster using a hypergeometric test. *P*‐values were adjusted for FDR using the Benjamini–Hochberg method.

### Cluster‐specific mechanistic hypotheses generation

For each cluster, deregulated kinases and TFs were defined using an absolute value of 2 for the median/standard deviation score. The Carnival R package was used to generate systematic mechanistic networks of interactions between deregulated kinases and TFs for each cluster. Carnival was used with default parameters, except for the time limit parameter that was set to 10,800 s, and the mipGAP parameter was set to 0.2. The optimisation problem was solved using the CPLEX solver. The prior knowledge network was generated from the omnipathR R package, using the “import_all_interactions” function.

## Author contributions

**Abel Sousa:** Conceptualization; formal analysis; visualization; writing – original draft; writing – review and editing. **Aurelien Dugourd:** Formal analysis; visualization; writing – original draft; writing – review and editing. **Danish Memon:** Formal analysis; writing – review and editing. **Borgthor Petursson:** Formal analysis; writing – review and editing. **Evangelia Petsalaki:** Supervision; writing – review and editing. **Julio Saez‐Rodriguez:** Supervision; writing – review and editing. **Pedro Beltrao:** Conceptualization; supervision; writing – original draft; writing – review and editing.

## Disclosures and competing interests statement

JS‐R receives funding from GSK and Sanofi and consultant fees from Travere Therapeutics. JS‐R is an EAB member, and PB is an EAB and EPAB member. This has no bearing on the editorial consideration of this article for publication.

## Supporting information



Appendix

Dataset EV1

Dataset EV2

Dataset EV3

Dataset EV4

Dataset EV5

## Data Availability

The computational analyses were performed in R 4.2.2, and all the code is available under a GNU General Public License V3 in a GitHub project at the following url: https://github.com/abelfsousa/pancancer_prot_act. Data analysis and structuring was performed using the packages included in tidyverse 1.3.2, including dplyr 1.0.10 and tidyr 1.2.1. Plotting was done using ggplot2 3.4.0 and ComplexHeatmap 2.14.0. This study includes no data deposited in external repositories.
