# Supplementary material for: Pan‐Cancer landscape of protein activities identifies drivers of signalling dysregulation and patient survival
Source: Mol Syst Biol. 2023 Jan 23;19(3):e10631. doi: 10.15252/msb.202110631 (PMC9996241; doi:10.15252/msb.202110631)
Supplement: Supplementary file 1 — Appendix [file MSB-19-e10631-s002.pdf]

Appendix: Pan-Cancer landscape of protein activities identifies drivers of signalling dysregulation and patient survival

Abel Sousa[1,2,3,4], Aurelien Dugourd[5,6], Danish Memon[1], Borgthor Petursson[1], Evangelia Petsalaki[1], Julio Saez-Rodriguez[5], Pedro Beltrao[1,7#]

1 - European Molecular Biology Laboratory, European Bioinformatics Institute, Wellcome Genome Campus, Hinxton, CB10 1SD, Cambridge, UK
2 - Instituto de Investigação e Inovação em Saúde da Universidade do Porto (i3s), Rua Alfredo Allen 208, 4200-135, Porto, Portugal
3 - Institute of Molecular Pathology and Immunology of the University of Porto (IPATIMUP), Rua Júlio Amaral de Carvalho 45, 4200-135, Porto, Portugal
4 - Graduate Program in Areas of Basic and Applied Biology (GABBA), Abel Salazar Biomedical Sciences Institute, University of Porto, Rua de Jorge Viterbo Ferreira 228, 4050-313, Porto, Portugal
5 - Faculty of Medicine, and Heidelberg University Hospital, Institute for Computational Biomedicine, Heidelberg University, Heidelberg, Germany
6 - Faculty of Medicine, Institute of Experimental Medicine and Systems Biology, RWTH Aachen University, Aachen, Germany
7 - Institute of Molecular Systems Biology, ETH Zürich, 8093 Zürich, Switzerland
# correspondence to pbeltrao@ethz.ch

Table of contents

Supplementary Results **3**

Appendix Figure S1 - Sample size and Pearson's correlation between phosphorylation levels and corresponding protein abundances. **6**

Appendix Figure S2. Lists of kinase-substrate associations compiled in this study. **7**

Appendix Figure S3. Validation of kinase-substrate sources and kinase activity estimates in the cancer samples. **8**

Appendix Figure S4. Effects of genomic alterations on protein abundances. **9**

Appendix Figure S5. Effects of genomic alterations on protein activities. **10**

Appendix Figure S6. Impact of recurrent mutations on the MAPK/ERK signalling transduction pathway and phosphatases differentially expressed between BRAFV600E-mutated samples with high and low activity of BRAF. **11**

Appendix Figure S7. Examples of associations between the mutational status of TFs and their activities. **12**

Appendix Figure S8. Correlation of genetic associations metrics derived from Pan-cancer and tissue levels analyses. **13**

Appendix Figure S9. Projection of protein activities in low-dimensional spaces and kinase-TF associations. **14**

Appendix Figure S10. Kinase activity regulation in tumours and perturbed human conditions. **15**

Appendix Figure S11. Examples of kinase and TF activities associated with the overall survival of cancer patients. **16**

Appendix Figure S12. Cross-correlation matrix of tumour sample kinase-TF activity signature. **17**

Appendix Figure S13. Heatmap of clinical features over-representation. **18**

Appendix Figure S14. Most consistently deregulated kinase and TF activities in each cluster. **19**

Appendix Figure S15. Mechanistic hypotheses to connect the highlighted kinases and TFs of each cancer cluster. **20**

Supplementary Results

Multi-omic based stratification of tumour samples

We clustered tumour samples according to kinase and transcription factor activities. To do so, we built a cross correlation matrix of tumour samples (by correlating their combined signature of batch corrected kinase and TF normalised enrichment scores (NES)) (**Appendix Figure S12**). Based on visual inspection of the cross-correlation matrix, as well as information from applying silhouette and "within sum of square" methods, we decided to stratify tumour samples into 8 distinct groups (**Methods**).

We performed an over-representation analysis of clinical features in each cluster (**Appendix Figure S13**) to find functional similarities between samples that have different annotations. For example, we found enrichments for ovarian cancer (cluster 1), stage I lung cancer (cluster 2), serous/mucinous ovary and colon cancers (cluster 3), colon cancer (cluster 4) and breast cancer (cluster 5) samples. We found enrichments also for immunity infiltration states: Cluster 6 is marginally enriched in CD8+ inflamed samples and cluster 7 in inflamed (CD8-) mesenchymal and infiltrating lobular carcinoma.

TF and kinase activity characterization of tumour sample clusters

To characterise which kinases and TFs are the most consistently deregulated in each cluster, the mean of each protein activity was divided by its corresponding standard deviation, within each cluster respectively (**Appendix Figure S14**). There are several activity enrichments that are consistent with prior observations. For example, cluster 1 and 3 (over-represented with high grade serous ovarian cystadenocarcinoma (SOC)) show increased activity of ARID1A, which is frequently mutated with loss of function (but not loss of protein expression) in ovary cancers (Yachida *et al*, 2020). IRF1 down-regulation in cluster 1 is coherent with finding that its over-expression in SOC is associated with better prognosis and survival (Cohen *et al*, 2014). Additionally, NFKB was found to have a tumour suppressor role in low grade SOC, while we observe a coherent down-regulation of NFKB1, RELA and SPI1 in cluster 1. Cluster 3 (also over-represented in high grade SOC) displays a different protein dysregulation profile. Cluster 3 also shows an up-regulation of KDM5B activity, previously found to be associated with high grade SOC and poor prognosis (Wang *et al*, 2015). TF4AP is a transcription factor associated with SOC proliferation activated down-stream of oestrogen signalling, and is also up-regulated in cluster 3 (O'Donnell *et al*, 2005).

In cluster 2 (lung adenocarcinoma (LUAD) over-represented) BHLHE40 is consistently up-regulated, which is coherent with reports showing its up-regulation in lung and esophageal carcinoma (Kiss *et al*, 2020). E2F6 activity, whose transcript levels are often elevated in LUAD, was also found to be up-regulated. Like cluster 2, cluster 5 is also over-represented with LUAD but also in breast cancer (BRCA) and shows a different protein dysregulation profile than cluster 2. Cluster 5 shows a high activity of ZEB2, a promoter of epithelial to mesenchymal transition (EMT), metastasis and resistance in LUAD and BRCA (Duan *et al*, 2016; Cui *et al*, 2019; Li *et al*, 2017). Coherently, MF2C activity is also inhibited in cluster 5. MF2C is known to be specifically inhibited by miR-223 (secreted by stromal cells) to increase invasion and migration of LUAD (Alečković & Kang, 2015).

Cluster 4 (over-represented with colorectal cancer (COREAD)) shows a high activity of BCL6, a novel potential therapeutic target of COREAD (Sun *et al*, 2020). Cluster 6 (over-represented in kidney cancer (ccRCC)) is characterised by a down-regulation of SMARCC2 activity. SMARCC2 is a core subunit of the tumour suppressor complex SWI/SNF and is emerging as a mechanism driving tumour development in murine models and ccRCC patients (Nargund *et al*, 2017; Agaimy *et al*, 2018). Cluster 7 (over-represented by CD8- inflamed tissues) shows a high activation of NFKB1. Interestingly, RUNX1 is also up-regulated, although it usually suppresses inflammation through NFKB1 inhibition (Bellissimo *et al*, 2020). JUN is also up-regulated in cluster 7, which is coherent with its role in the regulation of inflammation in epithelial tissues (Schonthaler *et al*, 2011). Cluster 8 (over-represented with uterus cancer patients) shows an up-regulation of BCL3, MAZ and MTA2. BCL3 and MTA2 have been found to be up-regulated in uterus cancer (Lin *et al*, 2020) while MAZ dysregulation seems to be under-studied in cancer.

Systematic exploration of mechanistic hypotheses to connect dysregulated kinases and TF in tumour clusters

After highlighting the most consistently deregulated kinases and TFs in each cluster, we sought to investigate the mechanistic links that could connect them. In particular, we searched which kinases could best explain the downstream deregulated TFs. For that we contextualised a prior knowledge network (PKN) obtained from Omnipath using the top deregulated kinases and TFs of each cluster with CARNIVAL (**Methods**) (Dugourd *et al*, 2021; Liu *et al*, 2019). The 8 causal reasoning networks generated had an average of 75 edges (54 s.d.). In each network, we can explore how given kinases are activating/inhibiting downstream signalling proteins. Kinases and TFs are connected by coherent causal interactions through intermediate signalling proteins (proteins for which there were not enough measurements to estimate their activities). Thus, the contextualised causal links also help us to hypothesise the potential activity of intermediate nodes (**Appendix Figure S15**).

In cluster 1, IRF1, RELA and SPI1 appear to be down-regulated as a consequence of the coordinate up-regulation of PRKCQ and down-regulation of GSK3B, through the down-regulation of SRC and JUN.

In cluster 2, the up-regulation of BHLHE40 and MAZ can be explained by an upstream up-regulation of the PAK2 kinase, the subsequent up-regulation of TP53, and the coordinated down-regulation of CDK1. PAK2 is commonly found over-expressed and activated in cancer (Ye & Field, 2012).

In cluster 3 we found an interesting signalling cascade in the form of SNAI2 inhibition downstream of the HIPK2 kinase. This can be particularly interesting because SNAI2 is another marker of aggressiveness and chemotherapy resistance in ovarian cancer, especially to cisplatin treatment (Fan *et al*, 2020), while HIPK2 over-expression is associated with sensitivity to paclitaxel (Li *et al*, 2010).

In cluster 4, the increased activity of NCOR2, BCL6 and MNT can be explained by the upstream activation of MAPK14. Notably, MAPK14, NCOR2 and BLC6 are all well known to promote survival and resistance to stress in tumour cells, while suppressing their growth (Battaglia *et al*, 2010; Grossi *et al*, 2014; Cardenas *et al*, 2017). This cascade is also coherent with the predicted down-regulation of CHUCK downstream of MAP3K8.

In cluster 5, the down-regualtion of RBPJ and SPI1 combined with the up-regulation ZEB2 can be explained by the activation of HDAC1 downstream of CSNK2A2 kinase. The activation of HDAC1 seems to be relevant for cancer progression (Cao *et al*, 2017), which is highlighted here by its ability to inhibit senescence pathways on one hand (through SPI1 inhibition) while promoting metastasis on the other hand (through ZEB2 activation) (Delestré *et al*, 2017).

In cluster 6, the down-regulation of MYOD1, SMARCC2, TFAP4 and PBX2 can be explained by the combined effect of MAPK1 down-regulation and PRKCZ up-regulation. The MAPK1 down-regulation could be mediated by the downstream inhibition of CREBBP. This is particularly relevant considering that CREBBP inhibition can either negatively or positively regulate proliferation and invasion of different types of kidney cancer cell lines (Wang *et al*, 2017). The network also shows RELA as an activated intermediate of PRKCZ, which is a potential therapeutic target in ccRCC (Peri *et al*, 2013).

In cluster 7, we can see that NFKB1, JUN, ETS1, SPI1 and RUNX1 are connected in a cascade. These up-regulated proteins are coherent with the over-representation of inflamed samples in this cluster. The combined action of PRKG1 and CDK5 seems to be facilitating the activation of NFKB1 and the rest of the cascade. In particular, NFKB1 is known to be a very important molecular effector of IFNG (Pfeffer, 2011). Coherently, IFNG is one of the most important secreted chemokines of CD8

independent immune response in cancer (Pluhar *et al*, 2015). These results suggest a cluster of tumour samples where the anti-tumoral response of the host is particularly active.

In cluster 8, we can see that the activation of BCL3 can be explained as a direct consequence of CDK5 activity. The deregulation of MAZ and HOXB13 can be explained as a consequence of the activity of CDK1 through CSNK2A1 and YY1. YY1 has been studied as a potential drug target in HPV-induced uterus cancer (He *et al*, 2011).

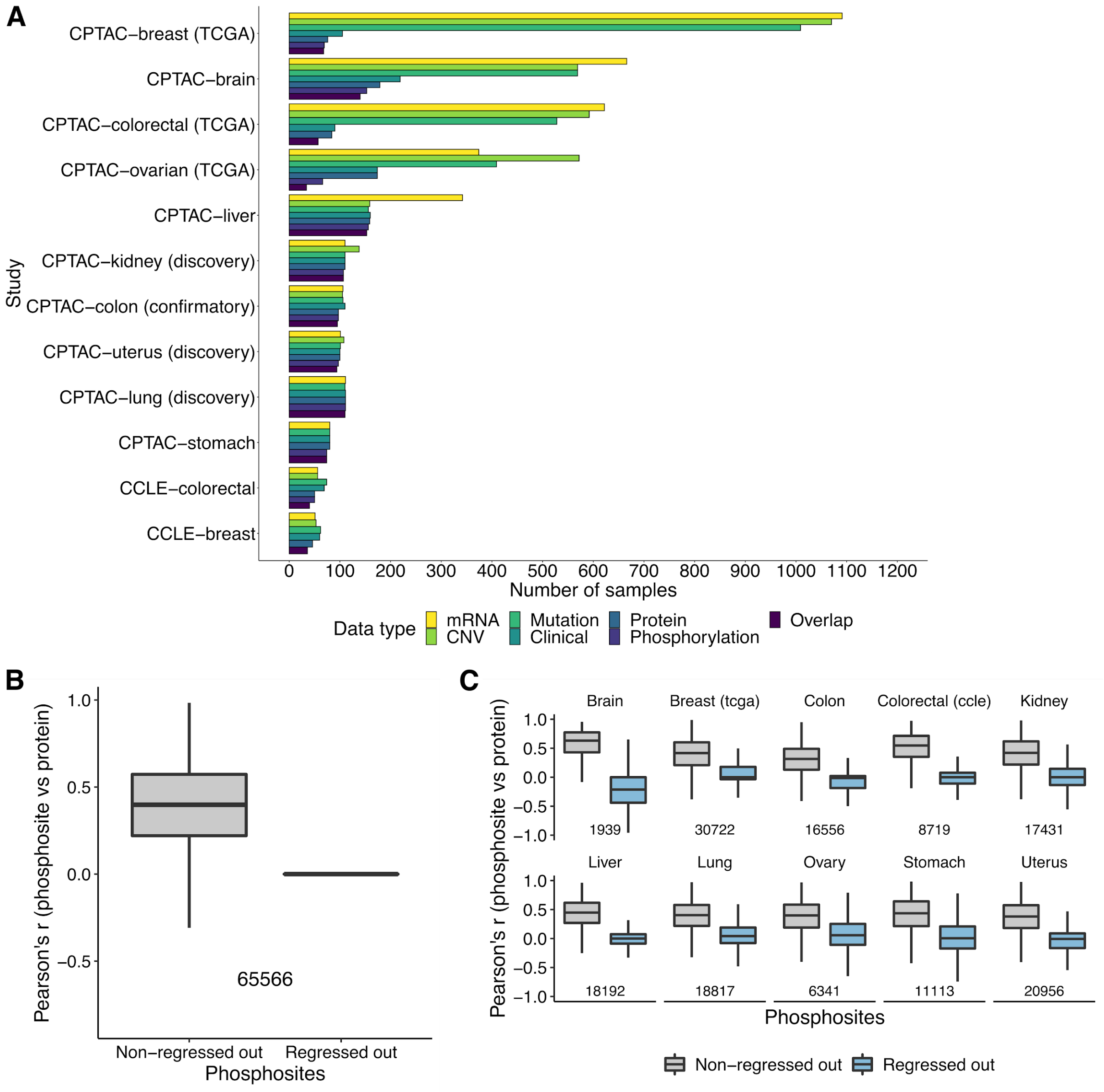

Appendix Figure S1 - Sample size and Pearson's correlation between phosphorylation levels and corresponding protein abundances.

(A) Number of samples by cancer dataset and data type.

(B) Distribution of the correlations between protein abundances and phosphorylation changes for protein-phosphosite pairs (number of pairs beneath the boxplots) across all cancer samples. Given the sparseness of the (phospho)proteomics data, we selected the protein-phosphosite pairs with protein/phosphorylation measures in at least 1% (n > 10) of the total cancer samples. Left: non-regressed-out phosphorylation data. Right: protein regressed-out phosphorylation data (Methods). **(C)** Representation of the same data as (B) by cancer dataset. Correlations were calculated for those protein-phosphosite pairs with protein/phosphorylation measures in at least 10% (n > 5) of the samples of each dataset. A small amount of correlation between phosphosites and proteins remains as the regression was done across all of the dataset.

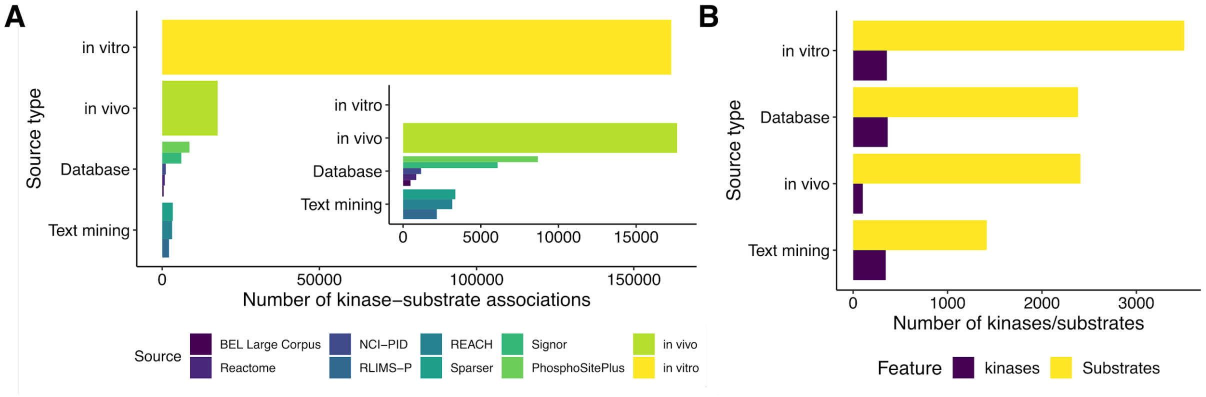

Appendix Figure S2. Lists of kinase-substrate associations compiled in this study.

(A) Number of kinase-substrate associations by source type.

(B) Number of kinases and substrates by source type.

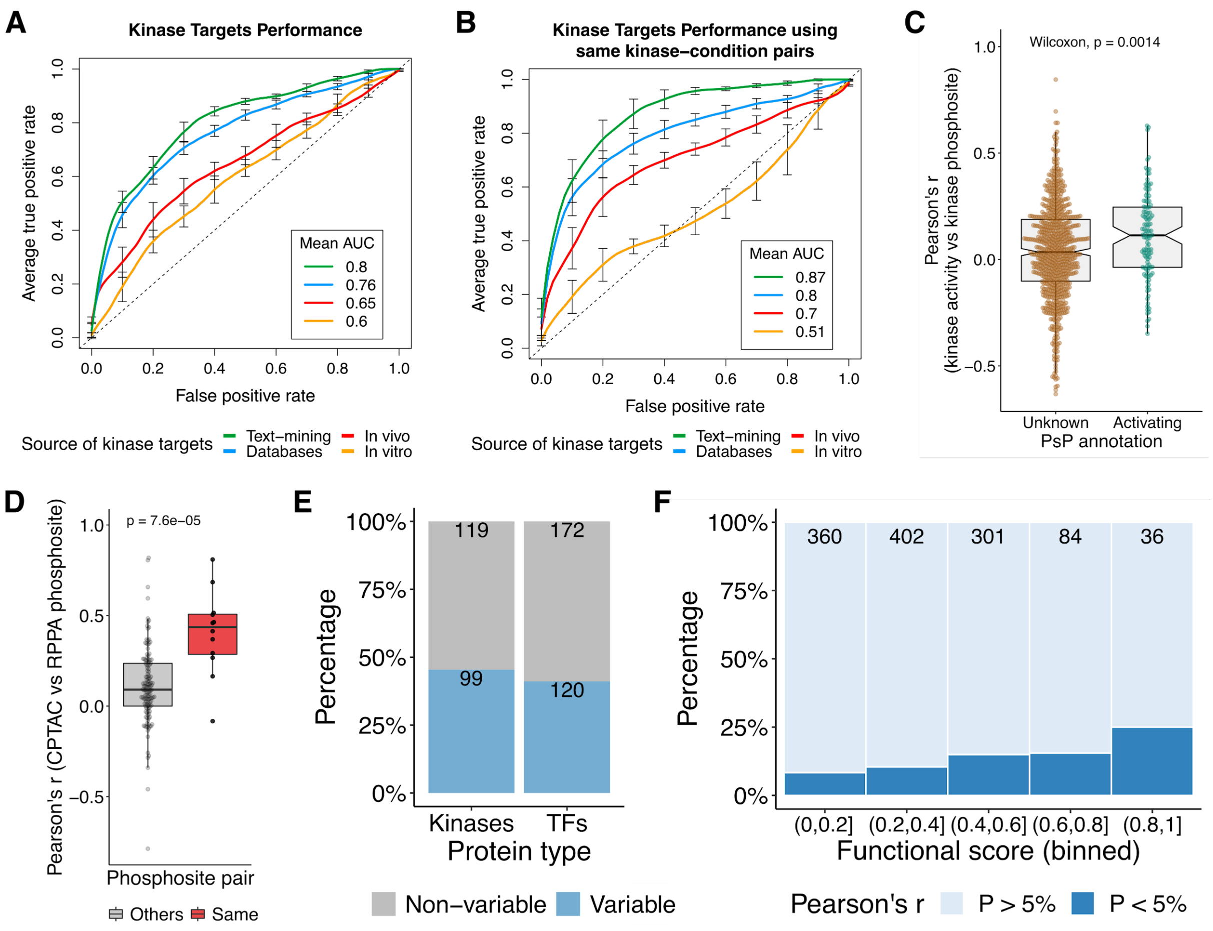

Appendix Figure S3. Validation of kinase-substrate sources and kinase activity estimates in the cancer samples.

(A) Receiver operating characteristic (ROC) curves demonstrating the predictive performance of the Z-test-based kinase activities across different sources of kinase-substrate interactions. As positives, we used a set of 184 kinase-condition pairs where regulation is expected to occur, while as negatives we generated 100 random sets of the same size as the positive set. Curves display the average of 100 ROC curves and vertical bars the standard deviation of the true positive rate at multiple points of false positive rate. The average area under the ROC curve (AUC) is shown for each kinase-substrate list. The averaged ROC curves and corresponding AUCs demonstrate the discriminative power of each kinase-substrate list.

(B) In contrast to the analysis shown in (A), here we replicated the 100 sets of negative (53) and positive (53) regulatory pairs along the different lists of kinase substrates.

(C) Related to the main Figure 1C. Kinase activities were re-estimated in cancer samples after removing the kinase auto-regulatory phosphosites from the kinase targets. The boxplots show the distribution of the Pearson’s correlation between kinase activities and phosphosite quantifications that mapped to the same kinase, with (n = 118) and without (n = 743) annotation (activating) in PhosphoSitePlus.

(D) Pearson's correlation between the CPTAC MS-based and the TCGA RPPA-based phosphosite quantifications, for the same phosphosite pair (n = 12) and others (n = 132). A P-value from a Wilcoxon rank sum test is shown.

(E) Fraction of kinases and TFs classified as highly variable across the tumour samples. Kinases and TFs with absolute activity measures higher than 1.75 and 3.89 (96.7th percentiles), respectively, in at least 5% of the samples were classified as highly variable.

(F) Percentage of phosphosites in TFs significantly and not significantly correlated with the corresponding TF activities, stratified by their functional score. Analysis based on 1,183 phosphosites mapping to 178 TFs (n > 10).

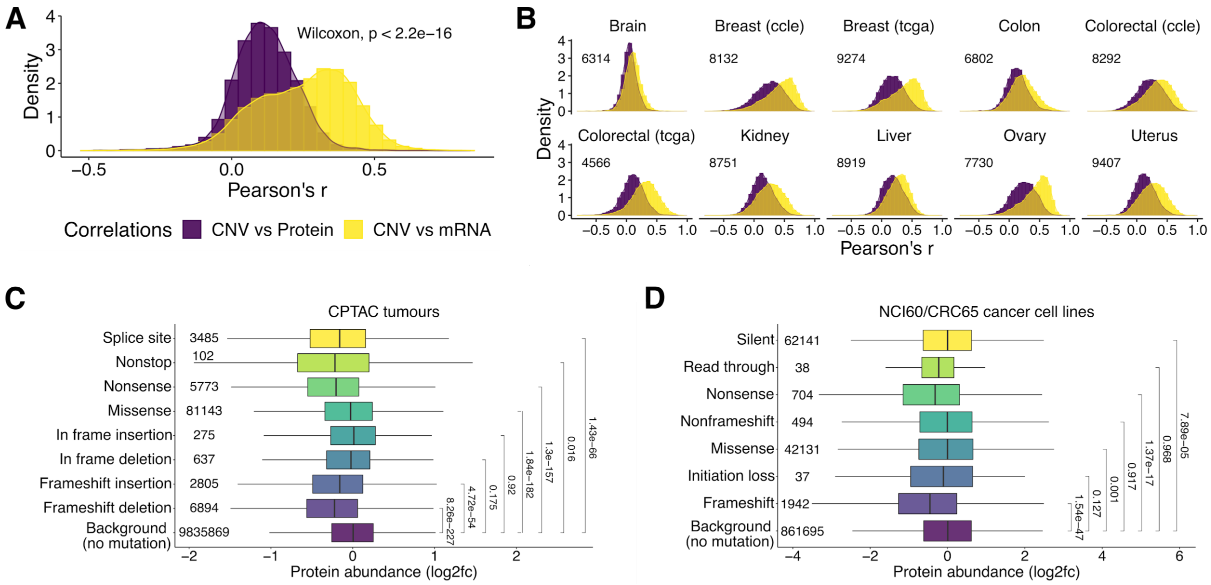

Appendix Figure S4. Effects of genomic alterations on protein abundances.

(A) Comparison of the distribution of the correlations (Pearson's r) between the CNV levels (GISTIC2) and the mRNA and protein abundances (log2 fold-changes). Correlations were calculated for those genes with CNV, mRNA and protein quantifications in at least 10 samples (11,624 genes). The experimental batch was regressed-out from the mRNA and protein quantification data before computing the correlations (Methods).

(B) Same as (A) by tissue and experimental study. P-values < 2.2e-16 in all cases (Wilcoxon rank sum test). The number of genes is indicated in the plot.

(C) Protein abundance distribution between mutation types from the CPTAC tumours. One sample may have multiple mutations in the same protein. Therefore, we selected the sample-protein pairs that were exclusive of each mutation type to prevent the cases where different mutations in the same protein and sample have the same protein abundance. The outliers (defined as the data points beyond Q1-1.5*IQR and Q3+1.5*IQR, where Q1 and Q3 are the first and third quartiles and IQR is the interquartile range) were removed from the distributions for representation

purposes. The number of protein quantifications (including outliers) is shown at the left of each boxplot. The P-values from a two-sample T-test comparing each distribution with the background (no mutation) are shown at the right. All data points (including outliers) were used to calculate the P-values.

(D) Same as (C) for the cancer cell lines from the NCI60 and CRC65 panels.

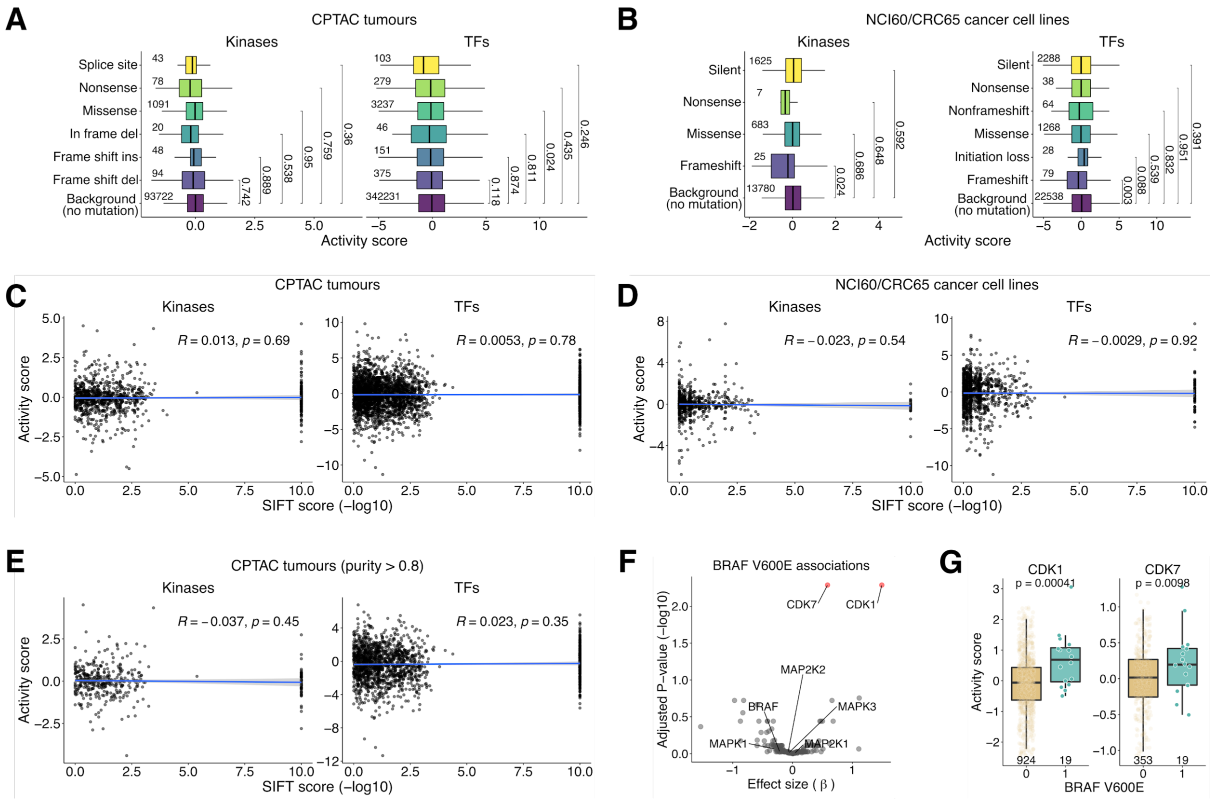

Appendix Figure S5. Effects of genomic alterations on protein activities.

(A) Distribution of kinase and TF activities between mutation types from the CPTAC tumours. Only the sample-protein pairs that were specific of each mutation type were selected to prevent the cases where different mutations in the same protein and sample have the same protein activity. The outliers (defined as the data points beyond Q1-1.5*IQR and Q3+1.5*IQR, where Q1 and Q3 are the first and third quartiles and IQR is the interquartile range) were removed from the distributions for representation purposes. The number of protein activity quantifications (including outliers) is shown at the left of each boxplot. The P-values from a two-sample T-test comparing each distribution with the background (no mutation) are shown at the right. All data points (including outliers) were used to calculate the P-values.

(B) Same as (A) for the cancer cell lines from the NCI60 and CRC65 panels.

(C) Scatterplots between the -log10 SIFT score (x-axis) of missense mutations and the activity of kinases and TFs (y-axis) from the CPTAC tumours. The linear regression line and the Pearson correlation coefficient, with the respective P-value, are shown. Cases where the same sample had multiple missense mutations in the

same gene were removed to prevent the assignment of the same protein activity to different SIFT scores.

(D) Same as (C) for the cancer cell lines from the NCI60 and CRC65 panels.

(E) Same as (C) for the CPTAC tumours with higher purity (greater than 0.8). The purity score provides information about the degree of immune infiltration and was calculated from the gene expression data using the ESTIMATE algorithm.

(F) Volcano plot showing the associations between the $BRAF^{V600E}$ mutation and the activity of kinases. The x-axis contains the mutation coefficient (effect size) and the y-axis the adjusted P-values. Highlighted are kinases from the MAPK/ERK signalling pathway and CDK1/7 (significantly associated with the $BRAF^{V600E}$ mutation).

(G) Differential activity of the CDK1 and CDK7 kinases between samples with and without $BRAF^{V600E}$ mutation. The x-axis separates the cancer samples by mutation status (1 if mutated and 0 otherwise) and the y-axis contains the kinase activities. The outliers were removed from the distributions for representation purposes. The number of quantifications (including outliers) are shown beneath each boxplot. A P-value from a Wilcoxon rank sum test comparing both distributions is shown. All data points (including outliers) were used to calculate the P-values.

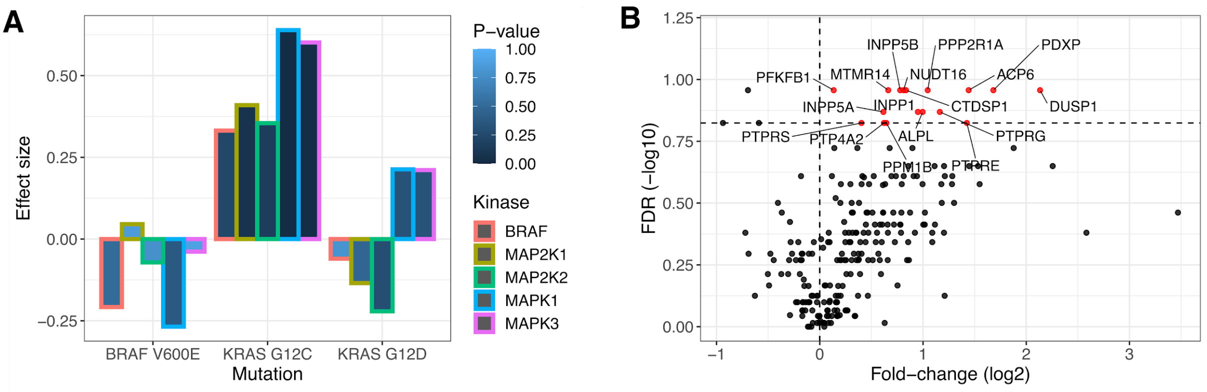

Appendix Figure S6. Impact of recurrent mutations on the MAPK/ERK signalling transduction pathway and phosphatases differentially expressed between $BRAF^{V600E}$-mutated samples with high and low activity of BRAF.

(A) The x-axis contains highly recurrent mutations in cancer - $BRAF^{V600E}$, $KRAS^{G12C}$ and $KRAS^{G12D}$. The y-axis shows the mutation coefficients (effect sizes) from multiple linear regression models fitted to predict the activities of kinases from the MAPK/ERK signalling transduction pathway using the mutations as predictors. The mutation coefficient P-values and predicted kinases are colour coded.

(B) The x-axis is the log2 fold-changes comparing the gene expression of phosphatases between $BRAF^{V600E}$-mutated samples with high and low activity of BRAF. Phosphatases with increased expression in samples with low BRAF activity have positive log2 fold-changes. The y-axis is the -log10 adjusted P-values from a Wilcoxon rank sum test. Marked in red are the phosphatases with a significant

difference (FDR < 15%) between the two sample groups and that tend to have higher expression levels in low BRAF activity samples.

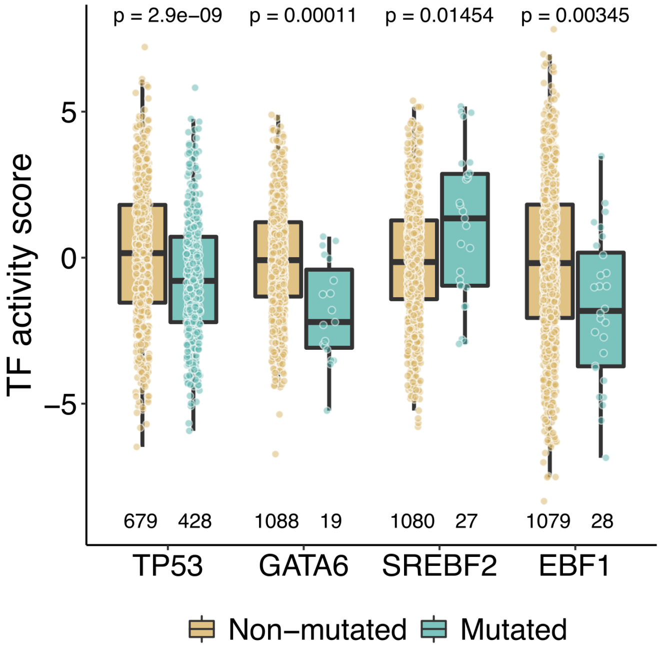

Appendix Figure S7. Examples of associations between the mutational status of TFs and their activities.

Related to the main Figure 2B. The x-axis represents the TFs and the y-axis the activities. The colours stratify the samples by their mutational status in the respective TFs. The number of quantifications are shown beneath each boxplot. The P-values from Wilcoxon rank sum tests comparing both distributions are shown.

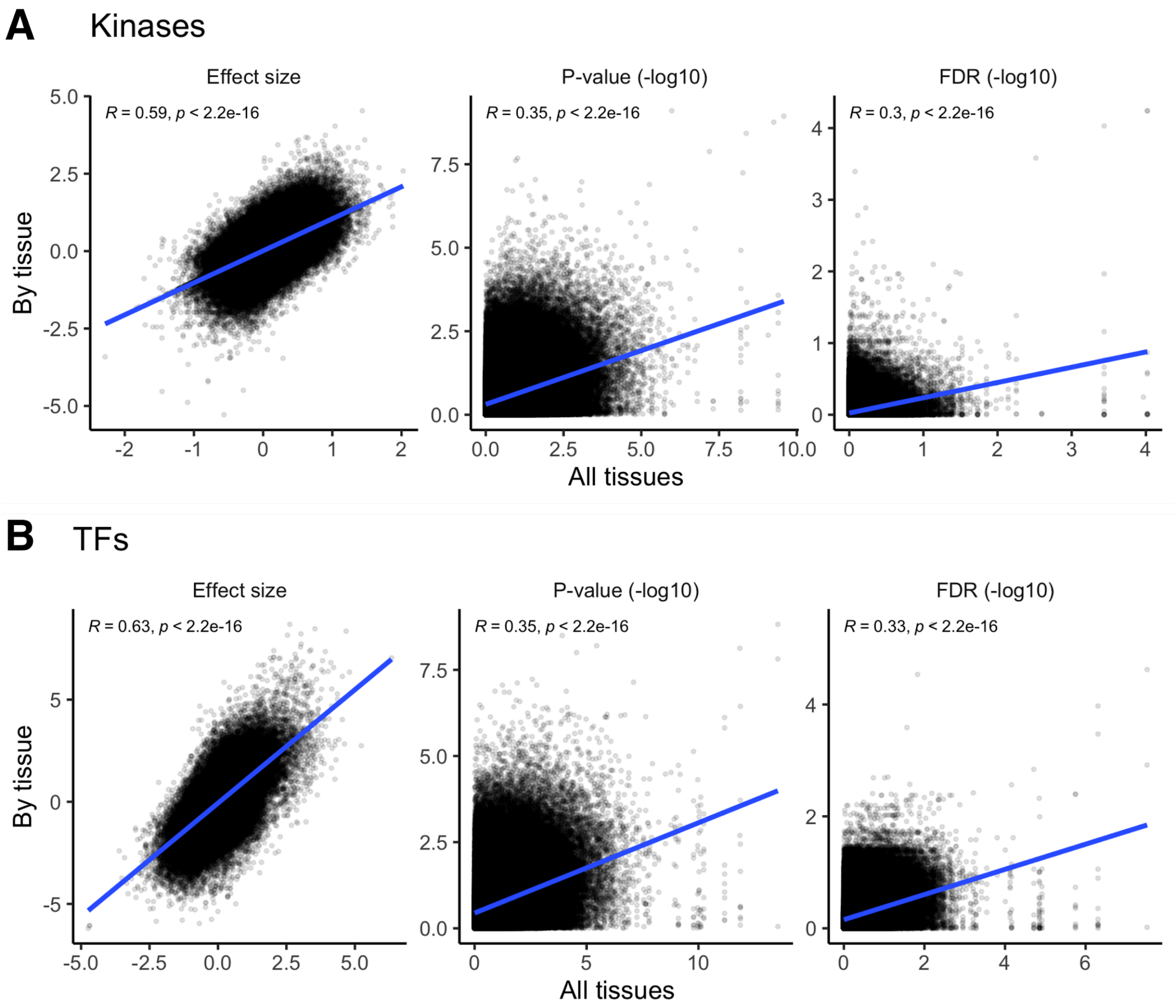

Appendix Figure S8. Correlation of genetic associations metrics derived from Pan-cancer and tissue levels analyses.

(A) Correlations of effect sizes (beta values) and -log10 P-values (non-adjusted and adjusted) of the associations between genetic variants and kinase activities at the Pan-cancer (x-axis) and tissue (y-axis) levels.

(B) Same as (A) for transcription factors.

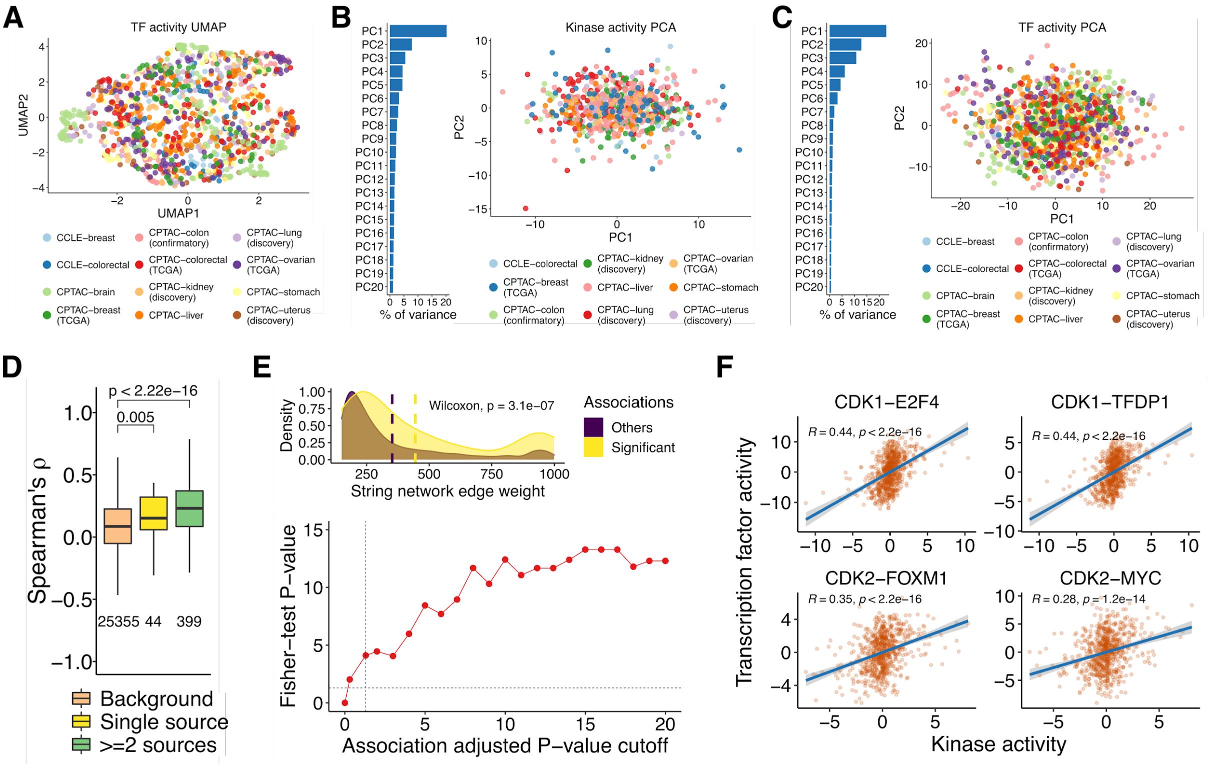

Appendix Figure S9. Projection of protein activities in low-dimensional spaces and kinase-TF associations.

(A) UMAP projection of the TF activity matrix (TFs as variables). The samples are colored by experimental study.

(B) PCA of the kinase activities. The barplots indicate the percentage of total variance explained by the first 20 principal components (PCs) (out of 90 PCs). The scatter plots illustrate the samples projected along the PC1 and PC2. The samples are colored by experimental study.

(C) Same as (B) for the TFs. The barplot contains 20 of 292 PCs.

(D) Related to the main Figure 3D. Correlations between the activities of non-redundant kinases with co-regulatory relationships. The co-regulatory interactions were obtained from OmniPath (activating and consensual interactions along the sources) and catalogued as present in a single source or in at least two different sources. The background corresponds to kinase pairs for which co-regulation is not known. The distributions were compared to the background using Wilcoxon rank sum tests.

(E) Top panel. String network edge weight distributions between the significant and non-significant kinase-TF associations (224 and 7527 pairs). The significant associations were selected with a FDR < 5% and an absolute effect size > 0.5. **Bottom panel.** Enrichment of the kinase-TF associations in the string network (edge weights > 850). The y-axis shows the Fisher-test P-values (-log10) and the x-axis the adjusted P-value cutoffs (-log10) that were used to select the associations.

(F) Scatter plots of the kinase-TF associations highlighted in the main Figure 3E.

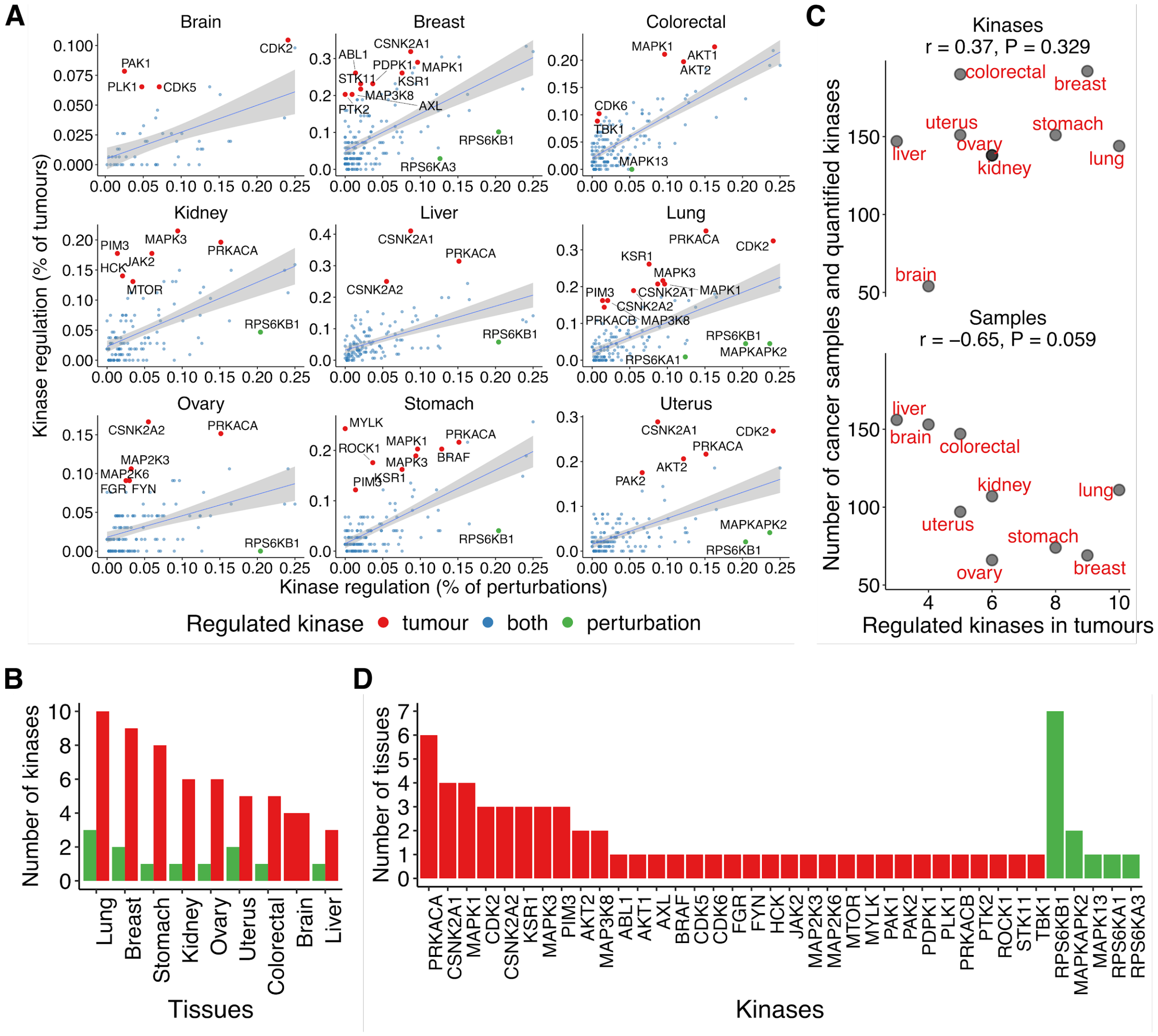

Appendix Figure S10. Kinase activity regulation in tumours and perturbed human conditions.

(A) Related to the main Figure 3F. Linear regression models between the percentage of kinase regulation in the perturbed conditions (x-axis) and in the tumour samples (y-axis) by tissue type.

(B) Number of kinases classified as regulated in the tumours (red) and in the conditions (green) in each tissue.

(C) Correlation between the number of regulated kinases in tumours (x-axis) and the number of quantified kinases and samples (y-axis) across tissues. The Pearson's r and respective P-value are shown.

(D) Number of tissues where the kinases were identified as regulated in the tumours (red) or in the conditions (green). The kinases are mutually exclusive between them (no kinase found as regulated in the tumours and in the conditions).

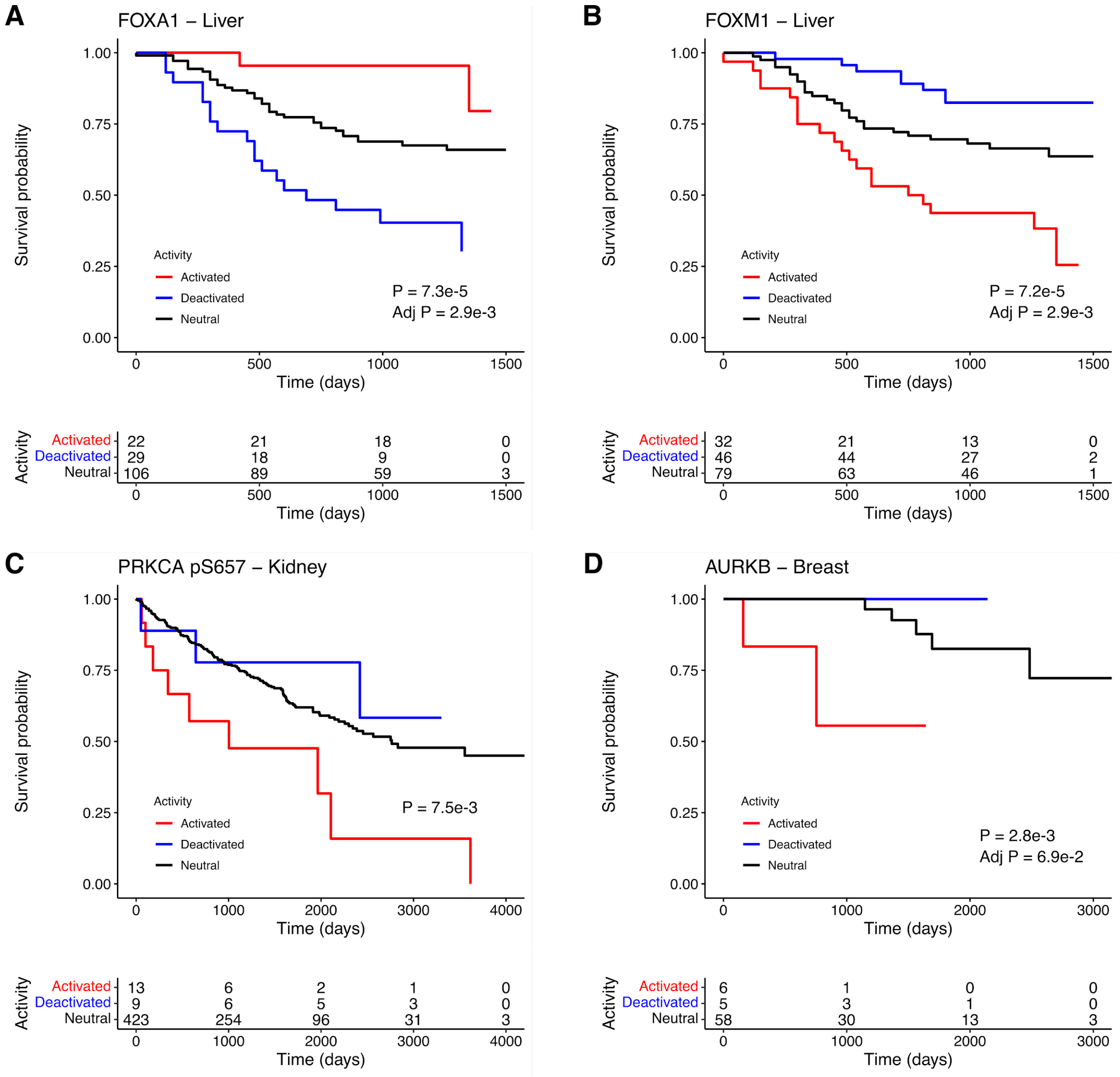

Appendix Figure S11. Examples of kinase and TF activities associated with the overall survival of cancer patients.

Kaplan-Meier survival plots related to the main Figures 4A-4B and 4F. The tables beneath each plot contain the number of individuals at risk across time. The log-rank P-values are shown in the plots.

(A) FOXA1 (inactive = 29, neutral = 106, active = 22) in liver cancer.

(B) FOXM1 (inactive = 46, neutral = 79, active = 32) in liver cancer.

(C) PRKCA pS657 (inactive = 9, neutral = 423, active = 13) in kidney cancer. Only the P-value is shown for PRKCA pS657 because no multiple hypothesis testing was performed using RPPA data.

(D) AURKB (inactive = 5, neutral = 58, active = 6) in breast cancer.

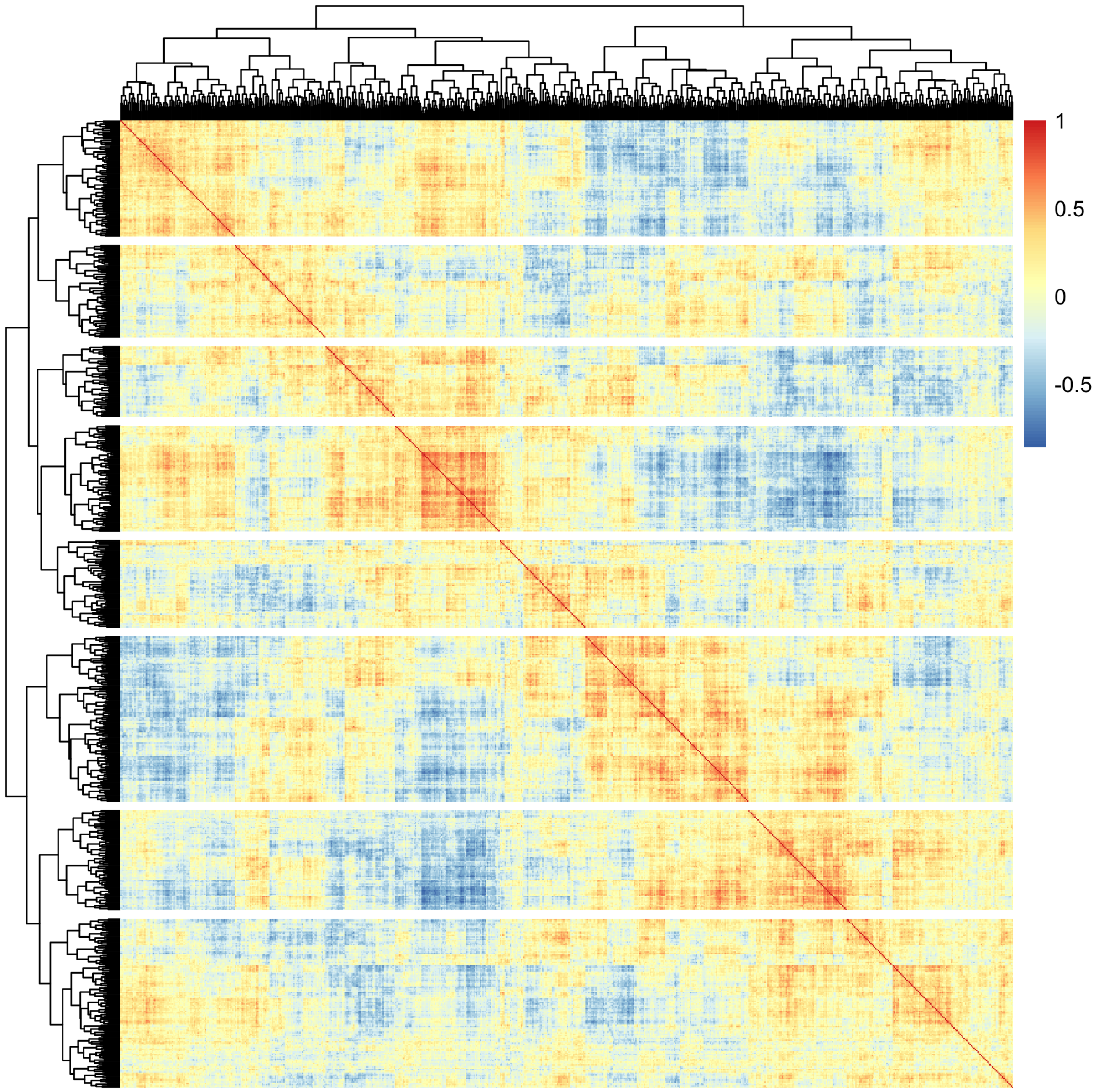

Appendix Figure S12. Cross-correlation matrix of tumour sample kinase-TF activity signature.

Each cell of the heatmap represents the spearman correlation coefficient between two samples. Correlations are estimated between pairs of vectors combining both TF and kinase activities. Dendrograms represent complete linkage hierarchical clustering from euclidean distances estimated based on the cross-correlation matrix. Each row/column represents a tumour sample.

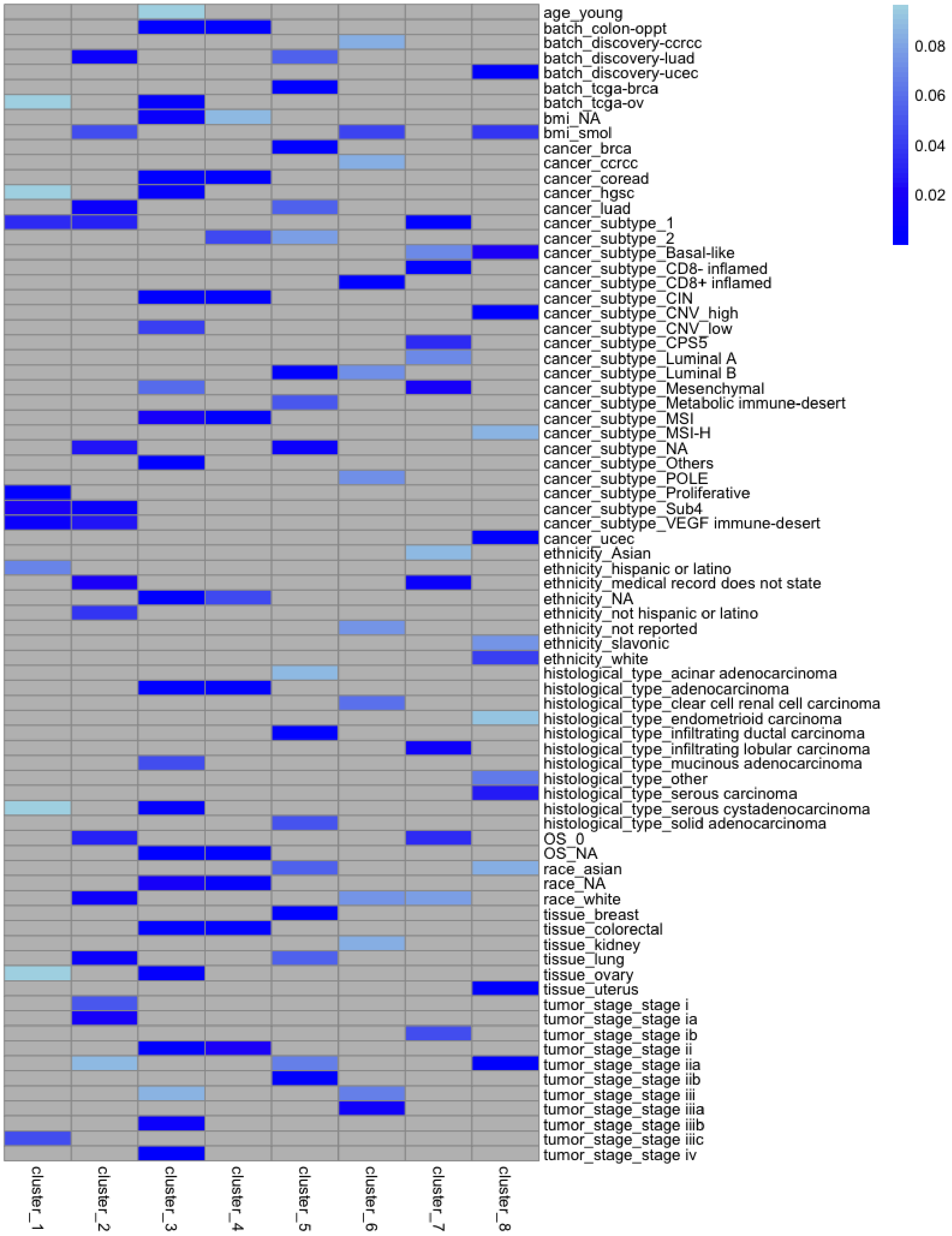

Appendix Figure S13. Heatmap of clinical features over-representation.

Each column represents a given cluster, each row represents a given clinical feature, and each cell represents the P-value of the over-representation of the given clinical feature in the patients of the given cluster. Only P-values < 0.1 are represented with shades of blue, others are greyed out.

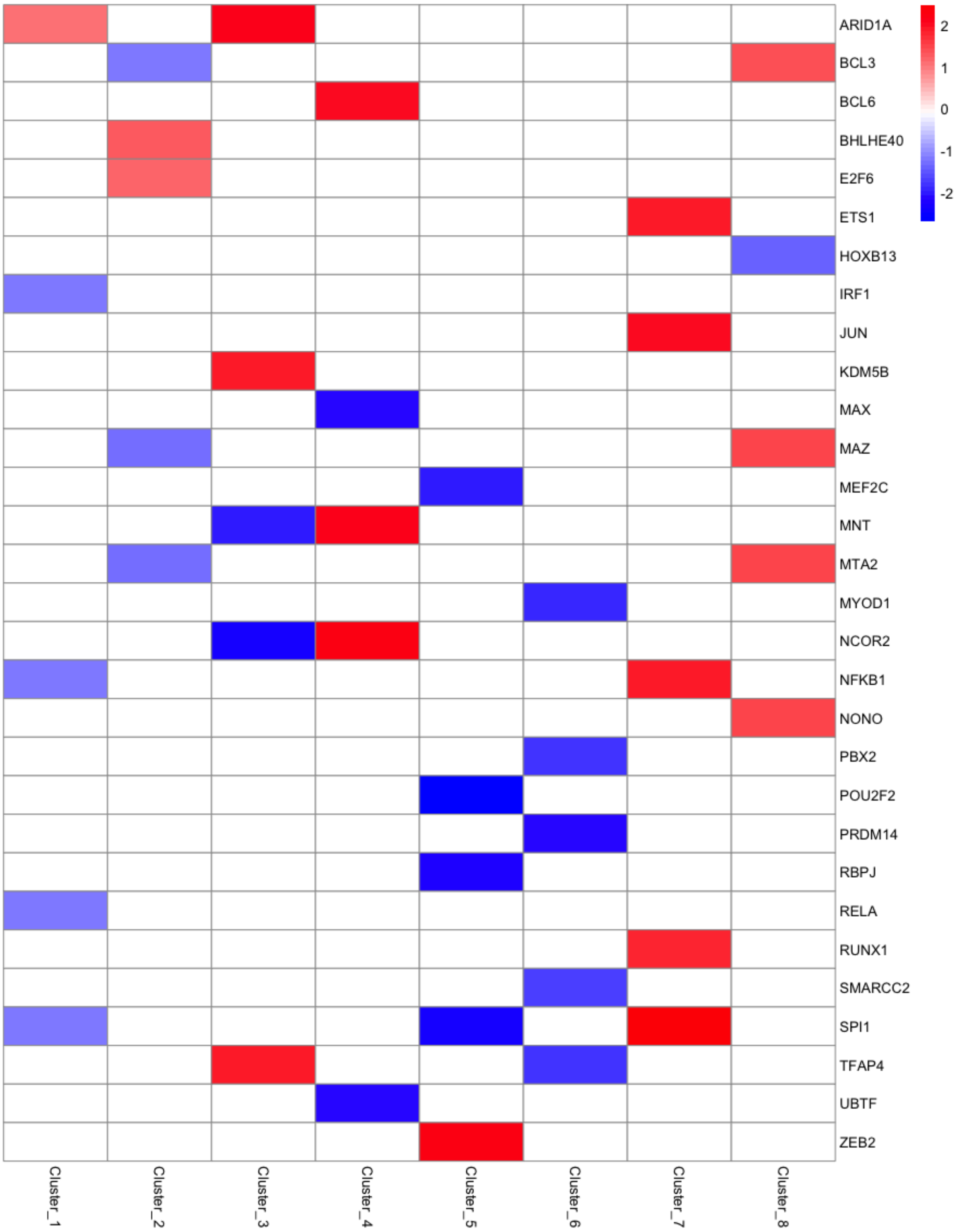

Appendix Figure S14. Most consistently deregulated kinase and TF activities in each cluster.

Each column represents a given cluster, each row represents a given TF/kinase, and each cell represents the scaled average of the TF/kinase scores across the patients

of the given cluster. Only absolute scaled average > 1.7 are displayed as shades of red and blue, others are whited out.

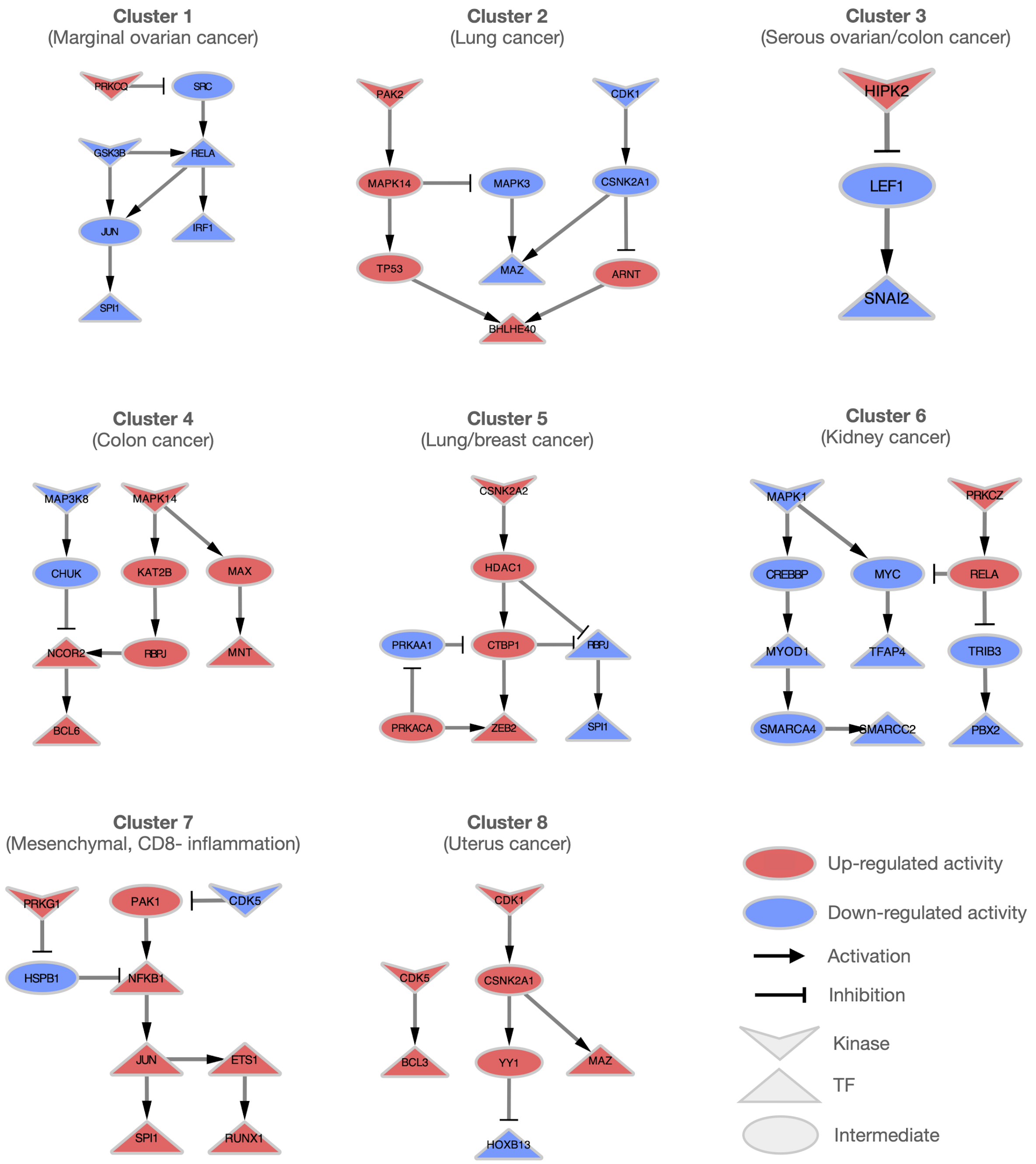

Appendix Figure S15. Mechanistic hypotheses to connect the highlighted kinases and TFs of each cancer cluster.

Appendix References

Agaimy A, Amin MB, Gill AJ, Popp B, Reis A, Berney DM, Magi-Galluzzi C, Sibony M, Smith SC, Suster S, *et al* (2018) SWI/SNF protein expression status in fumarate hydratase-deficient renal cell carcinoma: immunohistochemical analysis of 32 tumors from 28 patients. *Hum Pathol* 77: 139–146

Alečković M & Kang Y (2015) Regulation of cancer metastasis by cell-free miRNAs. *Biochim Biophys Acta* 1855: 24–42

Battaglia S, Maguire O & Campbell MJ (2010) Transcription factor co-repressors in cancer biology: roles and targeting. *Int J Cancer* 126: 2511–2519

Bellissimo DC, Chen C-H, Zhu Q, Bagga S, Lee C-T, He B, Wertheim GB, Jordan M, Tan K, Worthen GS, *et al* (2020) Runx1 negatively regulates inflammatory cytokine production by neutrophils in response to Toll-like receptor signaling. *Blood Adv* 4: 1145–1158

Cao L-L, Song X, Pei L, Liu L, Wang H & Jia M (2017) Histone deacetylase HDAC1 expression correlates with the progression and prognosis of lung cancer: A meta-analysis. *Medicine* 96: e7663

Cardenas MG, Oswald E, Yu W, Xue F, MacKerell AD Jr & Melnick AM (2017) The Expanding Role of the BCL6 Oncoprotein as a Cancer Therapeutic Target. *Clin Cancer Res* 23: 885–893

Cohen S, Mosig R, Moshier E, Pereira E, Rahaman J, Prasad-Hayes M, Halpert R, Billaud J-N, Dottino P & Martignetti JA (2014) Interferon regulatory factor 1 is an independent predictor of platinum resistance and survival in high-grade serous ovarian carcinoma. *Gynecol Oncol* 134: 591–598

Cui J, Pan G, He Q, Yin L, Guo R & Bi H (2019) MicroRNA-545 targets ZEB2 to inhibit the development of non-small cell lung cancer by inactivating Wnt/β-catenin pathway. *Oncol Lett* 18: 2931–2938

Delestré L, Cui H, Esposito M, Quiveron C, Mylonas E, Penard-Lacronique V, Bischof O & Guillouf C (2017) Senescence is a Spi1-induced anti-proliferative mechanism in primary hematopoietic cells. *Haematologica* 102: 1850–1860

Duan X, Fu Z, Gao L, Zhou J, Deng X, Luo X, Fang W & Luo R (2016) Direct interaction between miR-203 and ZEB2 suppresses epithelial-mesenchymal transition signaling and reduces lung adenocarcinoma chemoresistance. *Acta Biochim Biophys Sin* 48: 1042–1049

Dugourd A, Kuppe C, Sciacovelli M, Gjerga E, Gabor A, Emdal KB, Vieira V, Bekker-Jensen DB, Kranz J, Bindels EMJ, *et al* (2021) Causal integration of multi-omics data with prior knowledge to generate mechanistic hypotheses. *Mol Syst Biol* 17: e9730

Fan L, Lei H, Zhang S, Peng Y, Fu C, Shu G & Yin G (2020) Non-canonical signaling

pathway of SNAI2 induces EMT in ovarian cancer cells by suppressing miR-222-3p transcription and upregulating PDCD10. *Theranostics* 10: 5895–5913

Grossi V, Peserico A, Tezil T & Simone C (2014) p38α MAPK pathway: a key factor in colorectal cancer therapy and chemoresistance. *World J Gastroenterol* 20: 9744–9758

He G, Wang Q, Zhou Y, Wu X, Wang L, Duru N, Kong X, Zhang P, Wan B, Sui L, *et al* (2011) YY1 is a novel potential therapeutic target for the treatment of HPV infection-induced cervical cancer by arsenic trioxide. *Int J Gynecol Cancer* 21: 1097–1104

Kiss Z, Mudryj M & Ghosh PM (2020) Non-circadian aspects of BHLHE40 cellular function in cancer. *Genes Cancer* 11: 1–19

Li M-Z, Wang J-J, Yang S-B, Li W-F, Xiao L-B, He Y-L & Song X-M (2017) ZEB2 promotes tumor metastasis and correlates with poor prognosis of human colorectal cancer. *Am J Transl Res* 9: 2838–2851

Lin C-L, Ying T-H, Yang S-F, Wang S-W, Cheng S-P, Lee J-J & Hsieh Y-H (2020) Transcriptional Suppression of miR-7 by MTA2 Induces Sp1-Mediated KLK10 Expression and Metastasis of Cervical Cancer. *Mol Ther Nucleic Acids* 20: 699–710

Liu A, Trairatphisan P, Gjerga E, Didangelos A, Barratt J & Saez-Rodriguez J (2019) From expression footprints to causal pathways: contextualizing large signaling networks with CARNIVAL. *NPJ Syst Biol Appl* 5: 40

Li Z, Hu S, Wang J, Cai J, Xiao L, Yu L & Wang Z (2010) MiR-27a modulates MDR1/P-glycoprotein expression by targeting HIPK2 in human ovarian cancer cells. *Gynecol Oncol* 119: 125–130

Nargund AM, Osmanbeyoglu HU, Cheng EH & Hsieh JJ (2017) SWI/SNF tumor suppressor gene PBRM1/BAF180 in human clear cell kidney cancer. *Mol Cell Oncol* 4: e1342747

O'Donnell AJM, Macleod KG, Burns DJ, Smyth JF & Langdon SP (2005) Estrogen receptor-alpha mediates gene expression changes and growth response in ovarian cancer cells exposed to estrogen. *Endocr Relat Cancer* 12: 851–866

Peri S, Devarajan K, Yang D-H, Knudson AG & Balachandran S (2013) Meta-analysis identifies NF-κB as a therapeutic target in renal cancer. *PLoS One* 8: e76746

Pfeffer LM (2011) The role of nuclear factor κB in the interferon response. *J Interferon Cytokine Res* 31: 553–559

Pluhar GE, Pennell CA & Olin MR (2015) $CD8^+$ T Cell-Independent Immune-Mediated Mechanisms of Anti-Tumor Activity. *Crit Rev Immunol* 35: 153–172

Schonthaler HB, Guinea-Viniegra J & Wagner EF (2011) Targeting inflammation by modulating the Jun/AP-1 pathway. *Ann Rheum Dis* 70 Suppl 1: i109–12

Sun N, Zhang L, Zhang C & Yuan Y (2020) miR-144-3p inhibits cell proliferation of colorectal cancer cells by targeting BCL6 via inhibition of Wnt/β-catenin signaling. *Cell Mol Biol Lett* 25: 19

Wang L, Mao Y, Du G, He C & Han S (2015) Overexpression of JARID1B is associated with poor prognosis and chemotherapy resistance in epithelial ovarian cancer. *Tumour Biol* 36: 2465–2472

Wang X, Cui H, Lou Z, Huang S, Ren Y, Wang P & Weng G (2017) Cyclic AMP responsive element-binding protein induces metastatic renal cell carcinoma by mediating the expression of matrix metallopeptidase-2/9 and proteins associated with epithelial-mesenchymal transition. *Mol Med Rep* 15: 4191–4198

Yachida N, Yoshihara K, Suda K, Nakaoka H, Ueda H, Sugino K, Yamaguchi M, Mori Y, Yamawaki K, Tamura R, *et al* (2020) ARID1A protein expression is retained in ovarian endometriosis with ARID1A loss-of-function mutations: implication for the two-hit hypothesis. *Sci Rep* 10: 14260

Ye DZ & Field J (2012) PAK signaling in cancer. *Cell Logist* 2: 105–116
