# Supplementary material for: Pan‐Cancer landscape of protein activities identifies drivers of signalling dysregulation and patient survival
Source: Mol Syst Biol. 2023 Jan 23;19(3):e10631. doi: 10.15252/msb.202110631 (PMC9996241; doi:10.15252/msb.202110631)
Supplement: Supplementary file 7 [file MSB-19-e10631-s001.pdf]

Pan-Cancer landscape of protein activities identifies drivers of signalling dysregulation and patient survival

Abel Sousa, Aurelien Dugourd, Danish Memon, Borgthor Petursson, Evangelia Petsalaki, Julio Saez-Rodriguez, and Pedro Beltrao
DOI: 10.15252/msb.202110631

Corresponding author(s): Pedro Beltrao (pbeltrao@ethz.ch)

Review Timeline:

Transfer from Review Commons:	6th Aug 21
Editorial Decision:	9th Aug 21
Revision Received:	4th Oct 22
Editorial Decision:	7th Nov 22
Revision Received:	6th Jan 23
Accepted:	9th Jan 23

Editor: Maria Polychronidou

Transaction Report: This manuscript was transferred to Molecular Systems Biology following peer review at Review Commons.

Review #1

1. How much time do you estimate the authors will need to complete the suggested revisions:

Estimated time to Complete Revisions (Required)

(Decision Recommendation)

Between 3 and 6 months

2. Evidence, reproducibility and clarity:

Evidence, reproducibility and clarity (Required)

This manuscript represents a comprehensive computational pan-cancer study of kinase and transcription factor (TF) activities in the context of genetic alteration and protein and mRNA abundance levels, and protein phosphorylation events. By integrating data from multiple sources, primarily of the CPTAC consortium, they predict molecular determinants of activity changes of TF and kinases and their downstream targets. The study represents a substantial amount of work and and novel findings with potential molecular mechanisms, broad proteogenomic insights and relevance to patient prognosis. However, it would be useful to describe at some depth how the study relates to goes beyond CPTAC publications. The association of phosphorylation for kinases and mRNA levels for TFs is interesting but I am not sure if this is novel enough to make it a major point in the abstract. I also have a concern of the fully pan-cancer nature of this study, and some other comments and recommendations below.

1. The study includes data for >1000 primary cancer samples and >70 cell lines which in most analyses are treated as a bulk sample set. The inter-tumoral heterogeneity of different cancer types may represent a major concern in this approach, as the correlations of kinases and TFs as well as their downstream targets may largely represent the molecular differences of various cancer types. Have the authors attempted to split their data based on cancer types to verify and refine the predictions? Most or all analyses in the paper are potentially affected; but in particular, analyses where a small subset of proteins/samples is selected for further study (eg. 99 kinases and 120 TFs strongly regulated in at least 5% of samples - are the samples of a single cancer type or a mixture?). A partially related sub-comment: Some of the datasets for individual cancer types are rather small (<50 samples) and I wonder if these are

sufficiently powered to identify cancer type specific regulatory and signalling relationships.
2. Associations with genetic variation of single nucleotide variants (cancer driver mutations) with kinase and TF activities is a particularly interesting part of the manuscript however it should be further developed. While the authors focus on mutations with loss-of-function effects (indels, truncators, stop loss) and claim to see fewer effects on substitutions (missense SNVs), they could consider the latter class at greater depth since these are the majority of cancer mutations and represent known driver mutations (potenially from a previous catalogue like https://pubmed.ncbi.nlm.nih.gov/29625053/). Recurrent hotspot mutations affecting the same amino acid residue are likely more interesting than non-recurrent mutations since they may represent activation mechanisms of oncogenes (https://pubmed.ncbi.nlm.nih.gov/23539594/). Also, some amino acid changes may rewire kinase binding motifs (https://pubmed.ncbi.nlm.nih.gov/25938373/). Associating protein phosphorylation levels with genetic alterations of phosphorylation sites and motifs may provide more mechanistic detail to these predictions.
3. It would strengthen the study if the authors used independent validation data to confirm some of their predictions of kinase/TF abundances and downstream network activities, survival associations, or other aspects. For example, TCGA provides RPPA data which represents a small set of protein abundance and/or phosphorylation levels, however at a much larger set of cancer samples (CPTAC and TCGA may use a subset of the same samples which should be excluded from this validation set).
4. Overall survival analysis in association with kinase activity is interesting and lends confidence to the study with respect to clinical relevance. A couple of additional suggestions and improvements: a) a Cox-proportional hazards analysis should be preferred over log-rank tests since the latter are overly sensitive towards small sample numbers b) covariates should be included in the survival analysis: patient age, sex, tumor stage and/or grade, and ANOVA analysis should show whether the kinase effect explains survival differences beyond the covariates, and c) can the authors use the kinase activity associations with patient survival to make an exploratory analysis of potentia druggable targets for existing drugs or reporposing for the different cancer types? Also, this is the place to prioritize external validation.

Minor comments:

- the manuscript needs some language review and editing. For example, singular and plural forms are mixed up a few times in the abstract and elsewhere ('mutational profiles .. has been characterized'). Some typos like 'recapilated'.
- the authors use the String network for protein-protein interaction analysis but String largely represents functional interactions predicted using gene expression data and in

silico analysis. How many of the detected interactions are experimentally confirmed protein-protein interactions?

3. Significance:

Significance (Required)

(integrated above)

Review #2

1. How much time do you estimate the authors will need to complete the suggested revisions:

Estimated time to Complete Revisions (Required)

(Decision Recommendation)

Less than 1 month

2. Evidence, reproducibility and clarity:

Evidence, reproducibility and clarity (Required)

The study by Sousa, et al. describes analysis of a large multi-omics dataset from nine different cancer types to elucidate relationships between genetic mutations and kinase and transcription factor activity. The authors go through a number of different comparisons that reveal interesting and sometimes surprising things about the biology of the tumors and signaling pathways. I have the following suggestions for improvement.

Comments

1. The results in the paper are convincing overall and highlight some important potins of biological interest - maybe the most important being that loss of function mutations do not manifest as changes in kinase activity. Though a caveat of this finding is that the analysis is on bulk tumor samples, and that different conclusions might be drawn if looking at single cells (or smaller samples) - the authors state this and there are not yet appropriate datasets/techniques to analyze global phosphorylation patterns in very

small samples.
2. The analysis described in the paper is quite dense and a number of sections need to be revised to improve clarity:
a. On page 4, second paragraph: the correlation described is between 'each protein and phosphosites that mapped to that protein' which is fairly clear, but the next sentence that describes first 'a correlation [across all pairs]' and then 'an average protein-phosphosite correlation' is confusing. It's not clear how these are different but one can guess that the first is talking about intra-protein correlation or intra-phosphosite correlation?
b. On page 5, third paragraph: This paragraph is just unclear what comparisons are being made and what the results are. How is 'strongly regulated' defined? CNV and mRNA are mentioned, but then correlation is only discussed for the TFs, not for the kinases, which makes it confusing - I assume that the correlation with kinases is low, but that should be reported.
c. Page 7, second paragraph: The first few sentences describe quite a lot of analysis in a very condensed form. I suggest it be expanded to help clarity.
d. Page 10, third paragraph: It was difficult to understand what was being described in this paragraph.
e. Page 11, first paragraph: the introduction of "cancer and non-cancer conditions" is very abrupt as the dataset being used was not adequately described (just "large panel of other conditions"). It is not clear what "a correlation between the degree of regulation of kinases in cancer and non-cancer conditions" means. How is this comparison done? The mention of inter-tissue variation is also not well-described.
f. Page 11, second paragraph. It is not clear how the samples were clustered - though it's described in Method, some more detail should be included in the Results section to give the reader an idea.
3. Throughout the paper and in the abstract the number of samples are listed several times with different numbers. I realize that this is likely because of the availability of data limiting some analyses - but the differences in numbers are distracting and confusing. It should be made clear how/why the numbers of samples are reduced for different analyses.
4. The comparison of RPPA with MS results is somewhat interesting, but doesn't seem to further the findings of the paper in any significant way. I would suggest that it be removed OR moved to a supplemental figure.
5. I found the summary of numbers of samples of different types in Figure 1A to be not terribly helpful. I think a table listing these numbers might be a more efficient way to convey the message here.
6. Figure 3C in the text is described as showing "that highly regulated kinases in the same pathway tend to be activated or inhibited across the same samples". The sentence following this one clarifies that this is for one pathway in particular - and that would be good to make clear (rather than the much more general way it's stated

now).
7. Page 11, second paragraph. The clustering is a potentially very useful piece of information - but there are no figures or other evidence presented that about this analysis, only a description of the different cluster numbers and their enrichment (what does that mean?)
8. The finding that mutations in kinases don't seem to manifest in reductions in their activity is a surprising and even provocative result. I think this portion of the analysis deserves further investigation - LoF mutations cause a reduction in protein abundance, protein abundance is poorly correlated with activity, LoF is also poorly correlated with activity, but other kinases complement. I guess a direct comparison of the effects of LoF versus protein abundance on activity would be useful. That is, how much of the effect of the LoF mutation on activity can be explained by the reduction in protein abundance? What could the other portion be explained by? The compensatory response explanation is very interesting, but is somewhat unsatisfying without a proposed mechanism (though it's not clear to me what that mechanism might be) and more information about that response.
9. In the current study the authors show that phosphorylation of activating sites on kinases are generally correlated with activity. And previously (Ochoa, et al. 2016) the authors had reported that some specific activating sites on kinases correlated well with their activity. However, Arshad 2019 pointed out cases for which this was not observed in subsets of the data used here. It would therefore be interesting to have more analysis of individual cases of activating (and inhibitory sites) on kinases and how well their phosphorylation reflected activity in the data. This seems to be an important point of regulation that may not always behave in a straightforward way (similar to the LoF finding here) - even if it does correlate on average over lots of kinases.
10. There are a few misspellings and grammatical mistakes that should be corrected:

a. Abstract: "...mutational profiles of thousands of tumors have been extensively characterized..."

b. Page 7. "Similar to our previous reports..."

c. Page 7. "...the genetic alterations are often recapitulated at the protein..."

d. Page 16. "In addition, kinases are very tractable drug targets..."

3. Significance:

Significance (Required)

Much of the understanding of cancer signaling and the impact of driver mutations is based on observations from genomics, and the current work shows that these assumptions might be incorrect in important ways - specifically that loss of function mutations result in direct downregulation of the kinase activity. Since kinases are common targets of drugs and drug development, and compensatory mechanisms (as suggested here) are important in understanding drug response and drug resistance, the significance of this study and it's view across many different cancer types, is fairly significant for our understanding.

I am a computational biologist who has helped generate some of the data used in this study and has published in the area of multi-omic integration to understand cancer signaling and kinase activity.

Revision Plan

Manuscript number: #RC-2021-00892
Corresponding author(s): Pedro, Beltrao

1. General Statements

Dear editor,

Please find below a point-by-point plan to respond to the concerns raised by the 2 reviewers. We note that the two reviewers appear to be very enthusiastic about the significance of the work: "*The study represents a substantial amount of work and novel findings with potential molecular mechanisms, broad proteogenomic insights and relevance to patient prognosis.*"; "*the significance of this study and it's view across many different cancer types, is fairly significant for our understanding*". Overall, the reviewers have suggested a few additional analyses and several points for clarification. Most analyses are straightforward controls, useful extensions or simple text clarifications and we don't expect any issues in addressing the concerns raised. Our responses are in blue.

Regards
Pedro

2. Description of the planned revisions

Reviewer comments:

Reviewer #1 (Evidence, reproducibility and clarity (Required)):

This manuscript represents a comprehensive computational pan-cancer study of kinase and transcription factor (TF) activities in the context of genetic alteration and protein and mRNA abundance levels, and protein phosphorylation events. By integrating data from multiple sources, primarily of the CPTAC consortium, they predict molecular determinants of activity changes of TF and kinases and their downstream targets. The study represents a substantial amount of work and and novel findings with potential molecular mechanisms, broad proteogenomic insights and relevance to patient prognosis. However, it would be useful to describe at some depth how the study relates to goes beyond CPTAC publications. The association of phosphorylation for kinases and mRNA levels for TFs is interesting but I am not sure if this is novel enough to make it a major point in the abstract. I also have a concern of the fully pan-cancer nature of this study, and some other comments and recommendations below.

Revision Plan

We thank the reviewer for the very positive remarks in relation to our work. We will revise the manuscript according to these concerns as described in detail in the following specific points.

1. The study includes data for >1000 primary cancer samples and >70 cell lines which in most analyses are treated as a bulk sample set. The inter-tumoral heterogeneity of different cancer types may represent a major concern in this approach, as the correlations of kinases and TFs as well as their downstream targets may largely represent the molecular differences of various cancer types. Have the authors attempted to split their data based on cancer types to verify and refine the predictions? Most or all analyses in the paper are potentially affected; but in particular, analyses where a small subset of proteins/samples is selected for further study (eg. 99 kinases and 120 TFs strongly regulated in at least 5% of samples - are the samples of a single cancer type or a mixture?). A partially related sub-comment: Some of the datasets for individual cancer types are rather small (<50 samples) and I wonder if these are sufficiently powered to identify cancer type specific regulatory and signalling relationships.
We agree in principle with the reviewer that pan-cancer analyses can result in spurious associations driven by tissue differences. We think we have taken precautions to avoid this by including the tumour type as covariates in the association models. However, we agree that it would be useful to repeat many of the analyses within each tumour type and compare with the pan cancer results. We will perform these analysis and highlight cases where there are robust associations/results that are seen across multiple tumour types. We have in fact already done this for several of the results presented so we are confident that this will not be a concern.

2. Associations with genetic variation of single nucleotide variants (cancer driver mutations) with kinase and TF activities is a particularly interesting part of the manuscript however it should be further developed. While the authors focus on mutations with loss-of-function effects (indels, truncators, stop loss) and claim to see fewer effects on substitutions (missense SNVs), they could consider the latter class at greater depth since these are the majority of cancer mutations and represent known driver mutations (potenially from a previous catalogue like https://pubmed.ncbi.nlm.nih.gov/29625053/). Recurrent hotspot mutations affecting the same amino acid residue are likely more interesting than non-recurrent mutations since they may represent activation mechanisms of oncogenes (https://pubmed.ncbi.nlm.nih.gov/23539594/). Also, some amino acid changes may rewire kinase binding motifs (https://pubmed.ncbi.nlm.nih.gov/25938373/). Associating protein phosphorylation levels with genetic alterations of phosphorylation sites and motifs may provide more mechanistic detail to these predictions.
We thank the reviewer for noting that this result on the association between mutation and activities

is an interesting part of the work. Given the non-intuitive nature of the result we have in fact invested quite some time in trying to understand it. We agree that these are useful extensions and will extend the analysis of this section along the lines suggested by the reviewer.

3. It would strengthen the study if the authors used independent validation data to confirm some of their predictions of kinase/TF abundances and downstream network activities, survival associations, or other aspects. For example, TCGA provides RPPA data which represents a small set of protein abundance and/or phosphorylation levels, however at a much larger set of cancer samples (CPTAC and TCGA may use a subset of the same samples which should be excluded from this validation set).
We thank the reviewer for the useful suggestion and we will attempt to replicate some of the associations with the RPPA data whenever possible.

4. Overall survival analysis in association with kinase activity is interesting and lends confidence to the study with respect to clinical relevance. A couple of additional suggestions and improvements: a) a Cox-proportional hazards analysis should be preferred over log-rank tests since the latter are overly sensitive towards small sample numbers b) covariates should be included in the survival analysis: patient age, sex, tumor stage and/or grade, and ANOVA analysis should show whether the kinase effect explains survival differences beyond the covariates, and c) can the authors use the kinase activity associations with patient survival to make an exploratory analysis of potentia druggable targets for existing drugs or reporposing for the different cancer types? Also, this is the place to prioritize external validation.
We agree with the reviewer but we have in fact already performed the analysis as the reviewer suggests. In page 13 of the manuscript "*To take into account the effects of possible confounding covariates, we performed a multivariate Cox regression analysis using the protein activity scores as a predictor, while controlling for conventional clinical covariates and the genotype of recurrently mutated genes (Methods)*". We will try to revise this section of the results to emphasize that we already use the approach suggested by the reviewer. We will also attempt to replicate some associations with the RPPA data if possible and as suggested here also make an exploratory analysis of potential drug targets.

Minor comments:

- the manuscript needs some language review and editing. For example, singular and plural forms are mixed up a few times in the abstract and elsewhere ('mutational profiles .. has been

characterized'). Some typos like 'recapilated'.
We will revise more carefully
- the authors use the String network for protein-protein interaction analysis but String largely represents functional interactions predicted using gene expression data and in silico analysis. How many of the detected interactions are experimentally confirmed protein-protein interactions?
We will compare the results using physical protein interaction data although the types of signaling interactions we are looking for are notoriously difficult to detect using most physical protein interaction approaches.

Reviewer #1 (Significance (Required)):

(integrated above)

Reviewer #2 (Evidence, reproducibility and clarity (Required)):

The study by Sousa, et al. describes analysis of a large multi-omics dataset from nine different cancer types to elucidate relationships between genetic mutations and kinase and transcription factor activity. The authors go through a number of different comparisons that reveal interesting and sometimes surprising things about the biology of the tumors and signaling pathways. I have the following suggestions for improvement.

Comments

1. The results in the paper are convincing overall and highlight some important potins of biological interest - maybe the most important being that loss of function mutations do not manifest as changes in kinase activity. Though a caveat of this finding is that the analysis is on bulk tumor samples, and that different conclusions might be drawn if looking at single cells (or smaller samples) - the authors state this and there are not yet appropriate datasets/techniques to analyze global phosphorylation patterns in very small samples.
We have replicated many of the findings in cell line data where at least there won't be an issue with different populations of cells. We can't however yet analyse data at single cell level so it is possible that the population nature of the analysis masks effects seen only in some sets of cells. As the reviewer notes, we have tried to caution the reader about this caveat but can further expand on it.

2. The analysis described in the paper is quite dense and a number of sections need to be revised to improve clarity:

a. On page 4, second paragraph: the correlation described is between 'each protein and phosphosites that mapped to that protein' which is fairly clear, but the next sentence that describes first 'a correlation [across all pairs]' and then 'an average protein-phosphosite correlation' is confusing. It's not clear how these are different but one can guess that the first is talking about intra-protein correlation or intra-phosphosite correlation?

We will improve the description of these sections

b. On page 5, third paragraph: This paragraph is just unclear what comparisons are being made and what the results are. How is 'strongly regulated' defined? CNV and mRNA are mentioned, but then correlation is only discussed for the TFs, not for the kinases, which makes it confusing - I assume that the correlation with kinases is low, but that should be reported.

We will improve the description of these sections

c. Page 7, second paragraph: The first few sentences describe quite a lot of analysis in a very condensed form. I suggest it be expanded to help clarity.

We will improve the description of these sections

d. Page 10, third paragraph: It was difficult to understand what was being described in this paragraph.

We will improve the description of these sections

e. Page 11, first paragraph: the introduction of "cancer and non-cancer conditions" is very abrupt as the dataset being used was not adequately described (just "large panel of other conditions"). It is not clear what "a correlation between the degree of regulation of kinases in cancer and non-cancer conditions" means. How is this comparison done? The mention of inter-tissue variation is also not well-described.

We will improve the description of these sections

f. Page 11, second paragraph. It is not clear how the samples were clustered - though it's described in Method, some more detail should be included in the Results section to give the reader an idea.

We will improve the description of the results section as requested.

3. Throughout the paper and in the abstract the number of samples are listed several times with different numbers. I realize that this is likely because of the availability of data limiting some analyses - but the differences in numbers are distracting and confusing. It should be made clear how/why the numbers of samples are reduced for different analyses.
The reviewer is correct that the differences are due to how much data is available for each omics and as a consequence the data that is available to different analyses. We will try to explain these differences more carefully.

4. The comparison of RPPA with MS results is somewhat interesting, but doesn't seem to further the findings of the paper in any significant way. I would suggest that it be removed OR moved to a supplemental figure.
We will move this panel to a supplementary figure. We think it is useful to show that a very different technology shows some agreement with the mass spectrometry measurements.

5. I found the summary of numbers of samples of different types in Figure 1A to be not terribly helpful. I think a table listing these numbers might be a more efficient way to convey the message here.
We will try the reviewer’s suggestion of having a table or an alternative figure that is more informative.

6. Figure 3C in the text is described as showing "that highly regulated kinases in the same pathway tend to be activated or inhibited across the same samples". The sentence following this one clarifies that this is for one pathway in particular - and that would be good to make clear (rather than the much more general way it's stated now).
The reviewer is correct that Figure 3C is in fact representing a specific pathway and we intended the sentence to describe a general observation that summarises the different pieces of evidence for this section of the results. We will revise this section to make the text clearer and to be specific about what Figure 3C represents.

7. Page 11, second paragraph. The clustering is a potentially very useful piece of information - but there are no figures or other evidence presented that about this analysis, only a description of the different cluster numbers and their enrichment (what does that mean?)
We provide the clustering figures and description as a supplementary set of results (Supplementary Results). We decided to present the results in this way because we have done an extensive review of the clusters but considered that this was a descriptive analysis that

although potentially useful could distract from the rest of the results. We decided therefore to keep the full description in a Supplementary Results section and highlight in the main text some of the most interesting observations. We are willing to move some of these figures to supplementary or main figures if the reviewer/editor feel would make sense.

8. The finding that mutations in kinases don't seem to manifest in reductions in their activity is a surprising and even provocative result. I think this portion of the analysis deserves further investigation - LoF mutations cause a reduction in protein abundance, protein abundance is poorly correlated with activity, LoF is also poorly correlated with activity, but other kinases complement. I guess a direct comparison of the effects of LoF versus protein abundance on activity would be useful. That is, how much of the effect of the LoF mutation on activity can be explained by the reduction in protein abundance? What could the other portion be explained by? The compensatory response explanation is very interesting, but is somewhat unsatisfying without a proposed mechanism (though it's not clear to me what that mechanism might be) and more information about that response.
We agree this is the most surprising aspect of our work which was also noted by the other reviewer. This has been in fact the aspect of the work that we devoted most attention to given that it is, as the reviewer notes, a provocative result. Together with the comments of the first reviewer we will follow up on these suggestions and expand this analysis. We agree that searching for potential modes of adaptation would be exciting and we will attempt to do this by splitting samples with specific mutations into those that show or do not show the expected activities and search for recurrent molecular changes (e.g. phosphatase expression) that may explain the adaptation.

9. In the current study the authors show that phosphorylation of activating sites on kinases are generally correlated with activity. And previously (Ochoa, et al. 2016) the authors had reported that some specific activating sites on kinases correlated well with their activity. However, Arshad 2019 pointed out cases for which this was not observed in subsets of the data used here. It would therefore be interesting to have more analysis of individual cases of activating (and inhibitory sites) on kinases and how well their phosphorylation reflected activity in the data. This seems to be an important point of regulation that may not always behave in a straightforward way (similar to the LoF finding here) - even if it does correlate on average over lots of kinases.
We thank the reviewer for the interesting idea. We have also noted that such kinase regulatory phosphosites don’t always correlate with the phosphorylation levels of their target. We will perform a more detailed analysis of this as per the reviewer’s suggestion.

10. There are a few misspellings and grammatical mistakes that should be corrected:
a. Abstract: "...mutational profiles of thousands of tumors have been extensively characterized..."
b. Page 7. "Similar to our previous reports..."
c. Page 7. "...the genetic alterations are often recapitulated at the protein..."
d. Page 16. "In addition, kinases are very tractable drug targets..."
We will correct and revise more carefully.

Reviewer #2 (Significance (Required)):

Much of the understanding of cancer signaling and the impact of driver mutations is based on observations from genomics, and the current work shows that these assumptions might be incorrect in important ways - specifically that loss of function mutations result in direct downregulation of the kinase activity. Since kinases are common targets of drugs and drug development, and compensatory mechanisms (as suggested here) are important in understanding drug response and drug resistance, the significance of this study and it's view across many different cancer types, is fairly significant for our understanding.

I am a computational biologist who has helped generate some of the data used in this study and has published in the area of multi-omic integration to understand cancer signaling and kinase activity.

We thank the reviewer for the positive remarks regarding the significance of the work.

3. Description of the revisions that have already been incorporated in the transferred manuscript

Please insert a point-by-point reply describing the revisions that were already carried out and included in the transferred manuscript. If no revisions have been carried out yet, please leave this section empty.

4. Description of analyses that authors prefer not to carry out

Please include a point-by-point response explaining why some of the requested data or additional analyses might not be necessary or cannot be provided within the scope of a revision.

Revision Plan

This can be due to time or resource limitations or in case of disagreement about the necessity of such additional data given the scope of the study. Please leave empty if not applicable.

1st Editorial Decision **9th Aug 2021**

Thank you for submitting your manuscript to Molecular Systems Biology. I have now read the manuscript and your preliminary response to the comments of the Review Commons reviewers. I would like to invite you to submit your revised manuscript to Molecular Systems Biology.

Overall, we agree with the reviewers that examining cancer signaling by integrating multi-omics data, and going beyond only using genomics data, is an interesting approach. The concerns raised by the two reviewers seem addressable and I think that your revision plan sounds promising. In particular, it would be important to address point #2 raised by reviewer #1 (i.e., expanding on the associations of cancer driver mutations with kinase and TF activities) and point #8 of reviewer #2 (i.e., examining potential modes of adaptation to mutations). Several other issues related to providing further validation and control analyses would also need to be carefully addressed. Regarding point #7 of reviewer #2 we think that there is no need to include more figures in the main text. The figures related to this analysis and any additional ones generated during revision can remain in the Appendix. However, as we typically do not allow "Supplementary Results", the information currently provided as "Supplementary Results" would need to be moved to the Results and/or the Appendix Figure Legends.

Please find below a point-by-point plan response to the reviewers. Our responses as well as the most relevant changes to the main manuscript are highlighted in blue.

Reviewer comments:
Reviewer #1 (Evidence, reproducibility and clarity (Required)):

This manuscript represents a comprehensive computational pan-cancer study of kinase and transcription factor (TF) activities in the context of genetic alteration and protein and mRNA abundance levels, and protein phosphorylation events. By integrating data from multiple sources, primarily of the CPTAC consortium, they predict molecular determinants of activity changes of TF and kinases and their downstream targets. The study represents a substantial amount of work and and novel findings with potential molecular mechanisms, broad proteogenomic insights and relevance to patient prognosis. However, it would be useful to describe at some depth how the study relates to goes beyond CPTAC publications. The association of phosphorylation for kinases and mRNA levels for TFs is interesting but I am not sure if this is novel enough to make it a major point in the abstract. I also have a concern of the fully pan-cancer nature of this study, and some other comments and recommendations below.
We thank the reviewer for the very positive remarks in relation to our work. We respond below to individual concerns that have been brought up. We would still like to make the point that, to our knowledge, there has not been a systematic analysis of the major modes of regulation of TFs and kinases in general. We agree that the fact that kinases are often regulated by phosphorylation is certainly nothing new. However it is not uncommon to see over-expression studies of kinases as a way to assess potential consequences of increased pathway activity. This specific analysis can suggest which TFs and kinases have changes in their known downstream targets strongly correlated with the expression level of TFs and kinases, or of changes in specific phosphorylation sites.. Nevertheless, we have revised the abstract, removing this particular sentence.

1. The study includes data for >1000 primary cancer samples and >70 cell lines which in most analyses are treated as a bulk sample set. The inter-tumoral heterogeneity of different cancer types may represent a major concern in this approach, as the correlations of kinases and TFs as well as their downstream targets may largely represent the molecular differences of various cancer types. Have the authors attempted to split their data based on cancer types to verify and refine the predictions? Most or all analyses in the paper are potentially affected; but in particular, analyses where a small subset of proteins/samples is selected for further study (eg. 99 kinases and 120 TFs strongly regulated in at least 5% of samples - are the samples of a single cancer type or a mixture?). A partially related sub-comment: Some of the datasets for individual cancer types are rather small (<50 samples) and I wonder if these are sufficiently powered to identify cancer type specific regulatory and signalling relationships.
We agree with the concern raised by the reviewer that pan-cancer analyses can result in spurious associations driven by tissue differences. We think we have already taken precautions to avoid this by including the tumor type as covariates in the association models. In this way, any association that would have been driven by the tumor type should be suppressed. To illustrate this we have followed the suggestion of the reviewer and repeated the analysis where

we perform associations between genetic variants and kinase/TF activity at tumor type level and compare the effect size and significance with the same result found at the Pan-cancer level. As shown in the figure below, the effect size and significance tend to be highly correlated when performing the analysis across all samples or samples within each tumor type. We think this illustrates well how, in our association models, we already use the tissue type as a cofounder to account for associations that are primarily driven by the tissue of origin.

As the reviewer also notes, this could mean that we could also search for associations that are more variable across tissue types. This could highlight cases of tissue specific differences. We decided against pursuing this direction because of the relatively small sample size for each tissue type and because such tissue variable associations will most likely also enrich potential errors. Associations that are consistent across tissues are more likely to be correct.

We have added this analysis as an additional supplementary figure and explain this result in the main text.

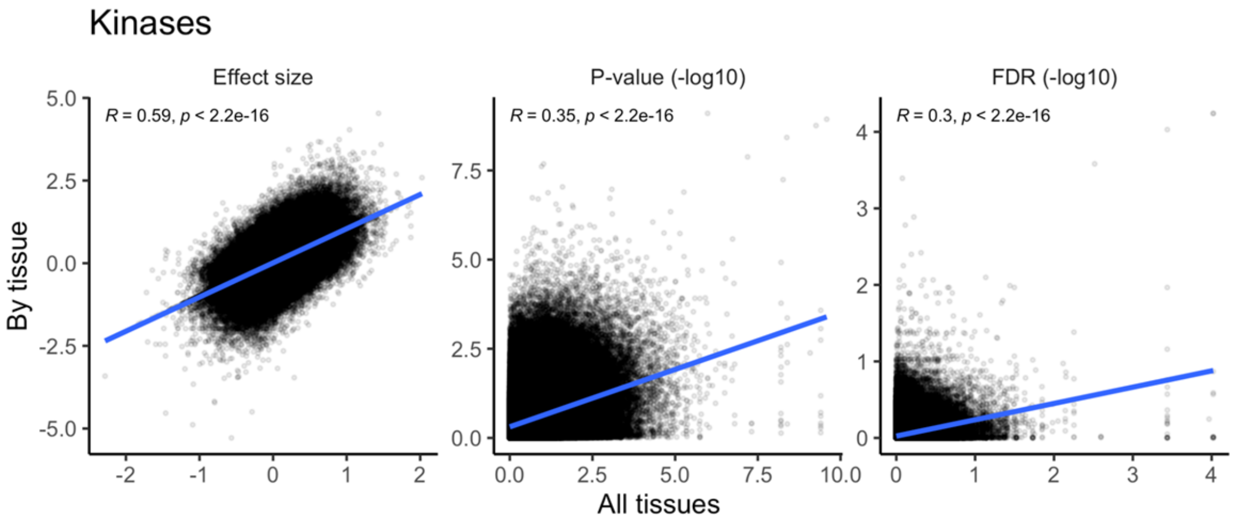

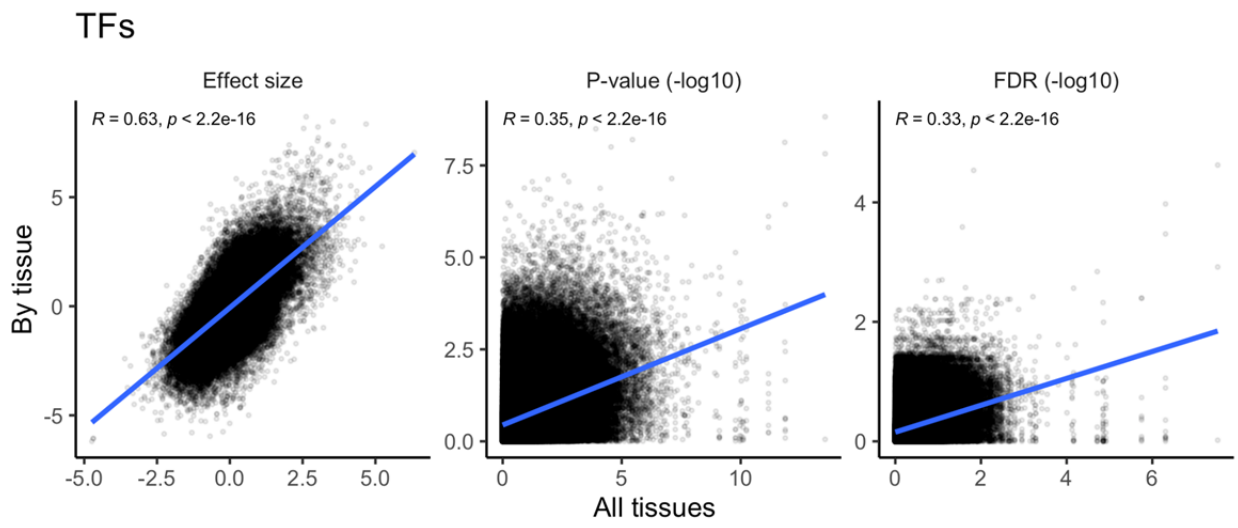

2. Associations with genetic variation of single nucleotide variants (cancer driver mutations) with kinase and TF activities is a particularly interesting part of the manuscript however it should be further developed. While the authors focus on mutations with loss-of-function effects (indels, truncators, stop loss) and claim to see fewer effects on substitutions (missense SNVs), they could consider the latter class at greater depth since these are the majority of cancer mutations and represent known driver mutations (potenially from a previous catalogue like https://pubmed.ncbi.nlm.nih.gov/29625053/). Recurrent hotspot mutations affecting the same amino acid residue are likely more interesting than non-recurrent mutations since they may represent activation mechanisms of oncogenes (https://pubmed.ncbi.nlm.nih.gov/23539594/). Also, some amino acid changes may rewire kinase binding motifs (https://pubmed.ncbi.nlm.nih.gov/25938373/). Associating protein phosphorylation levels with genetic alterations of phosphorylation sites and motifs may provide more mechanistic detail to these predictions.

We thank the reviewer for noting that this result on the association between mutation and activities is an interesting part of the work. Given the non-intuitive nature of the result we have in fact invested quite some time in trying to understand it. Although it may not have been very clear in the manuscript, we have also in fact performed an analysis of recurrent mutations, which include the known driver mutations. In aggregate, we don't observe significant associations between recurrent mutations in kinases and changes in the phosphorylation state of known down-stream targets.

Reviewer 2 was also interested in seeing some attempt to better understand the lack of association between mutations of known effect and their down-stream consequences. For this reason we expanded our analysis with a specific focus on recurrent mutations of BRAF and KRAS that are well known activating mutations of the same pathway. As we show in the figures below, for BRAF V600E and KRAS G12D we don't observe any trend for changes in regulation of targets of the kinases in the BRAF pathway. However, for KRAS G12D we do see the expected general trend for increased phosphorylation of targets of kinases in this pathway. However, the trend would not be significant when the association is searched for in unbiased

genome-wide fashion. That is, it would not be significant after considering the multiple testing performed.

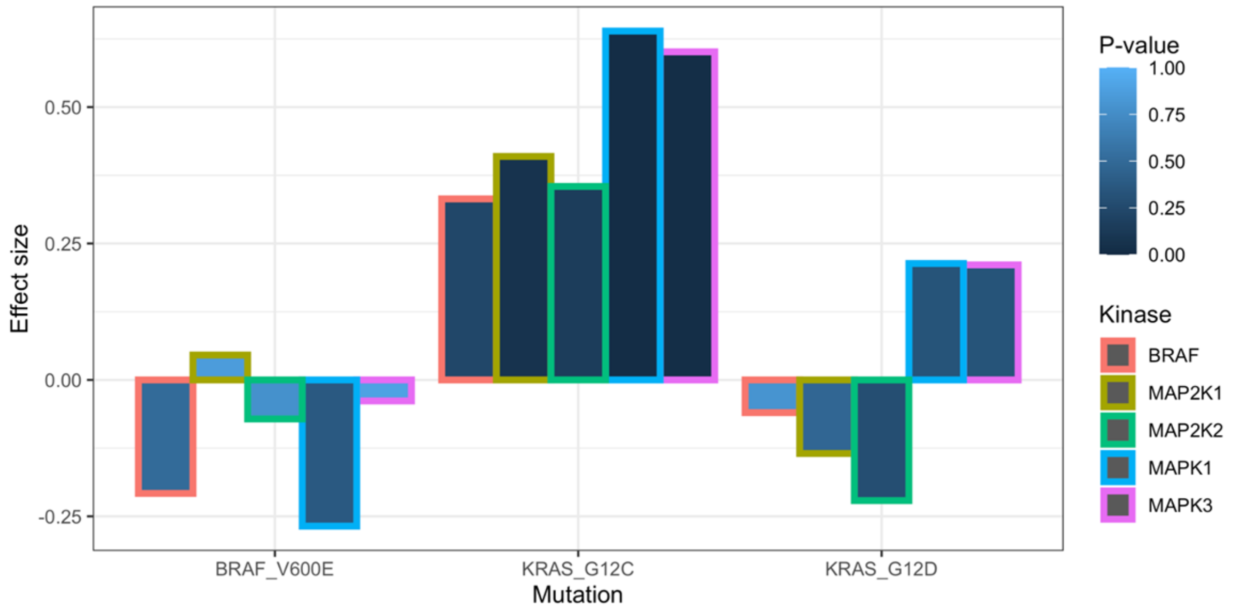

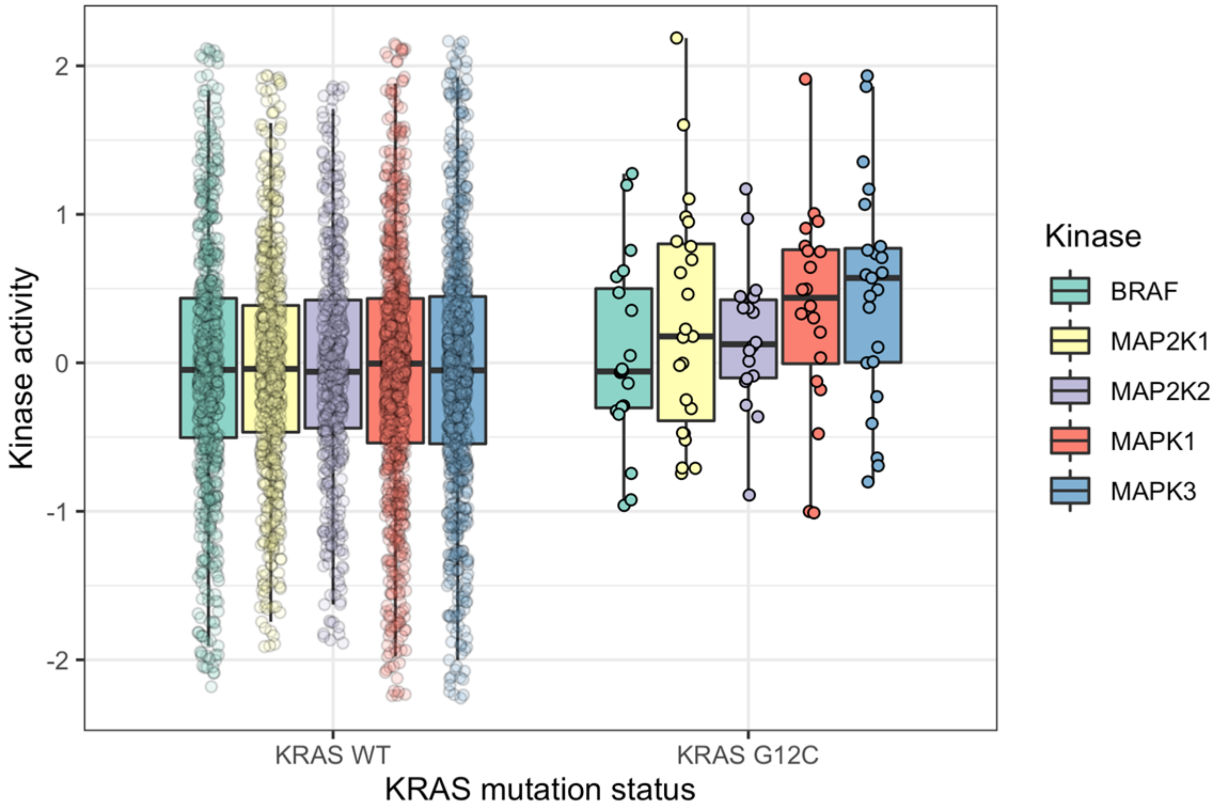

We then tried to find a potential reason why the BRAF V600E activating mutation is not reflected in the expected activation state of the kinases in the pathway. For this purpose, we have

selected the 19 samples with this mutation and compared the samples where BRAF activity was increased (6 samples) with samples where the BRAF activity was predicted to be decreased (8 samples). We reasoned that phosphatase levels could be a potential mechanism explaining a down-regulation of the pathway activity in the presence of an activating mutation. We therefore measured the fold change of phosphatase mRNA levels in these two groups of samples. Of 237 phosphatases tested, 74% tend to have higher expression levels in the samples with low BRAF activity, 17 of these have a significant difference across the two groups of samples with a false discovery rate set at 15% (figure below [red dots]). From this analysis we can extract the phosphatases that show the strongest correlation between their expression and the activation state of BRAF which includes DUSP1, a phosphatase known to regulate the MAPK pathway. We have added this analysis to the manuscript as an illustration of how these datasets could be used to search for potential adaptation mechanisms for further experimental testing.

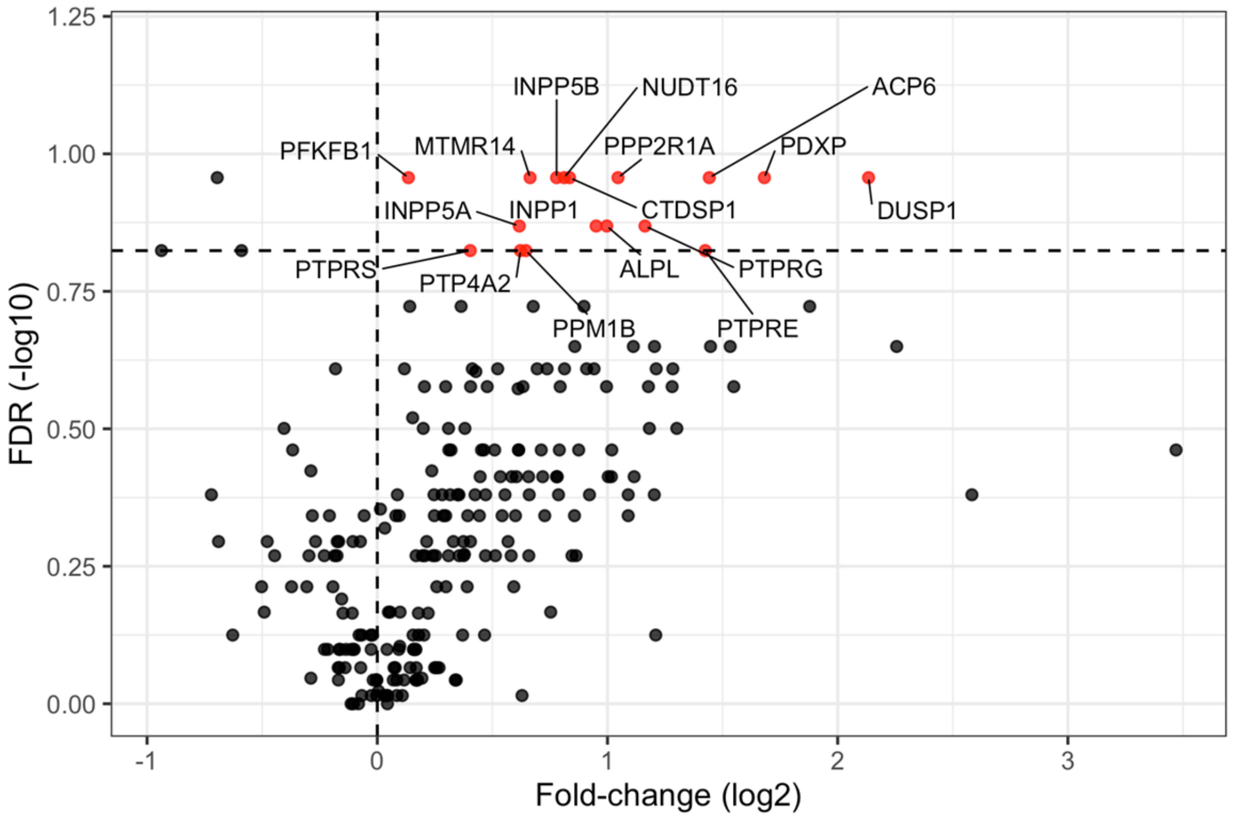

3. It would strengthen the study if the authors used independent validation data to confirm some of their predictions of kinase/TF abundances and downstream network activities, survival associations, or other aspects. For example, TCGA provides RPPA data which represents a small set of protein abundance and/or phosphorylation levels, however at a much larger set of cancer samples (CPTAC and TCGA may use a subset of the same samples which should be excluded from this validation set).

We thank the reviewer for the useful suggestion. We performed associations between genes carrying loss of function mutations and the activity levels of kinases as predicted by phosphorylation sites measured using RPPA. These associations were done for the same tumor

types but a much larger set of samples available in TCGA. We selected a total of 29 gene-kinase significant associations from this RPPA analysis and correlated the beta values of the associations between the RPPA-TCGA datasets with our analysis done based on CPTAC. Across these associations we find a correlation of 0.57 (p=0.0012). While there is much variation, it is not unexpected given the differences in methodology and samples. There are only 2% of samples (95 out of 4133) that overlap between the two analyses and we are comparing direct measurements of single phosphosite with antibody with kinase activity inference from phosphoproteomics datasets.

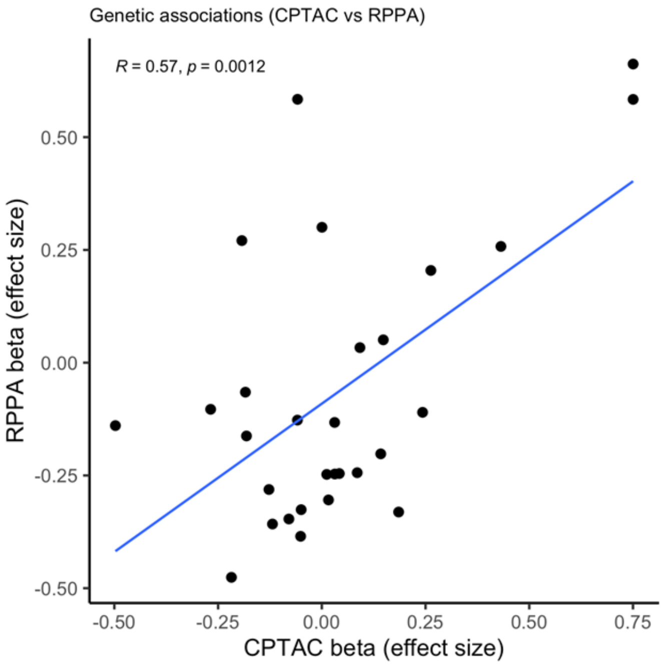

4. Overall survival analysis in association with kinase activity is interesting and lends confidence to the study with respect to clinical relevance. A couple of additional suggestions and improvements: a) a Cox-proportional hazards analysis should be preferred over log-rank tests since the latter are overly sensitive towards small sample numbers b) covariates should be included in the survival analysis: patient age, sex, tumor stage and/or grade, and ANOVA analysis should show whether the kinase effect explains survival differences beyond the covariates, and c) can the authors use the kinase activity associations with patient survival to make an exploratory analysis of potentia druggable targets for existing drugs or reporposing for the different cancer types? Also, this is the place to prioritize external validation.

We agree with the reviewer but we have in fact already performed the analysis as the reviewer suggests. In page 13 of the manuscript "*To take into account the effects of possible confounding covariates, we performed a multivariate Cox regression analysis using the protein activity scores as a predictor, while controlling for conventional clinical covariates and the genotype of recurrently mutated genes (Methods)*". In addition, in order to give further strength to this analysis we have tried to replicate some associations using the RPPA data and we were able to

validate the Kaplan-Meier survival curve for PRKCA in kidney cancer using RPPA phosphorylation data from TCGA (P-value = 7.5e-3, figure below). We would like to note that these samples from TCGA are not present in the CPTAC samples used in our study. We added this result to the manuscript. This suggests that PRKCA (PKCα) inhibitor could be a worthy drug candidate for additional experimental testing in kidney cancer. We didn't pursue this line more extensively due to resource constraints.

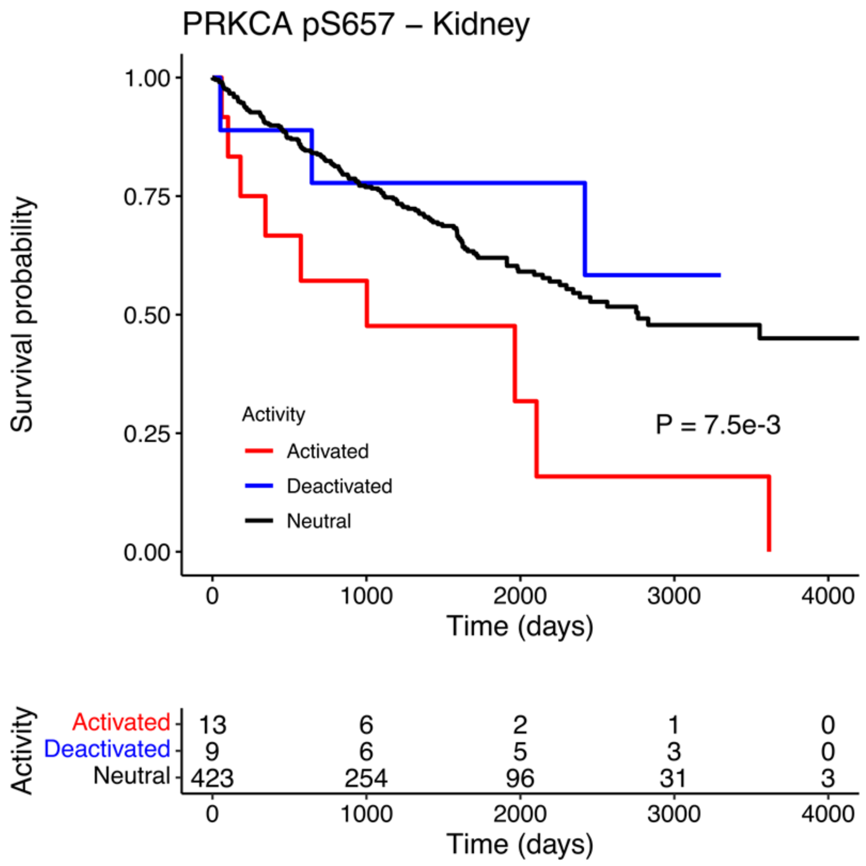

Minor comments:

- the manuscript needs some language review and editing. For example, singular and plural forms are mixed up a few times in the abstract and elsewhere ('mutational profiles .. has been characterized'). Some typos like 'recapilated'.

We have tried to revise the text to correct these issues.

- the authors use the String network for protein-protein interaction analysis but String largely represents functional interactions predicted using gene expression data and in silico analysis. How many of the detected interactions are experimentally confirmed protein-protein interactions?

We re-computed the enrichment analyses performed on our protein association pairs using a STRING subnetwork that is only composed of edges supported by physical interactions (9606.protein.physical.links.v11.5; https://string-db.org/cgi/download?sessionId=bLtDShGSnCib&species_text=Homo+sapiens). Across increasing cutoffs of statistical significance for the protein association pairs (x-axis), we observe that the statistical significance of the enrichment test (Fisher-test) also increases (y-axis) (figures below - enrichment for kinase-mutation, TF-mutation and kinase-TF associations). This result is consistent with the previous results using the full STRING network. We didn't include this analysis in the paper as it essentially serves as a control but we can add it if the reviewer feels it would be critical.

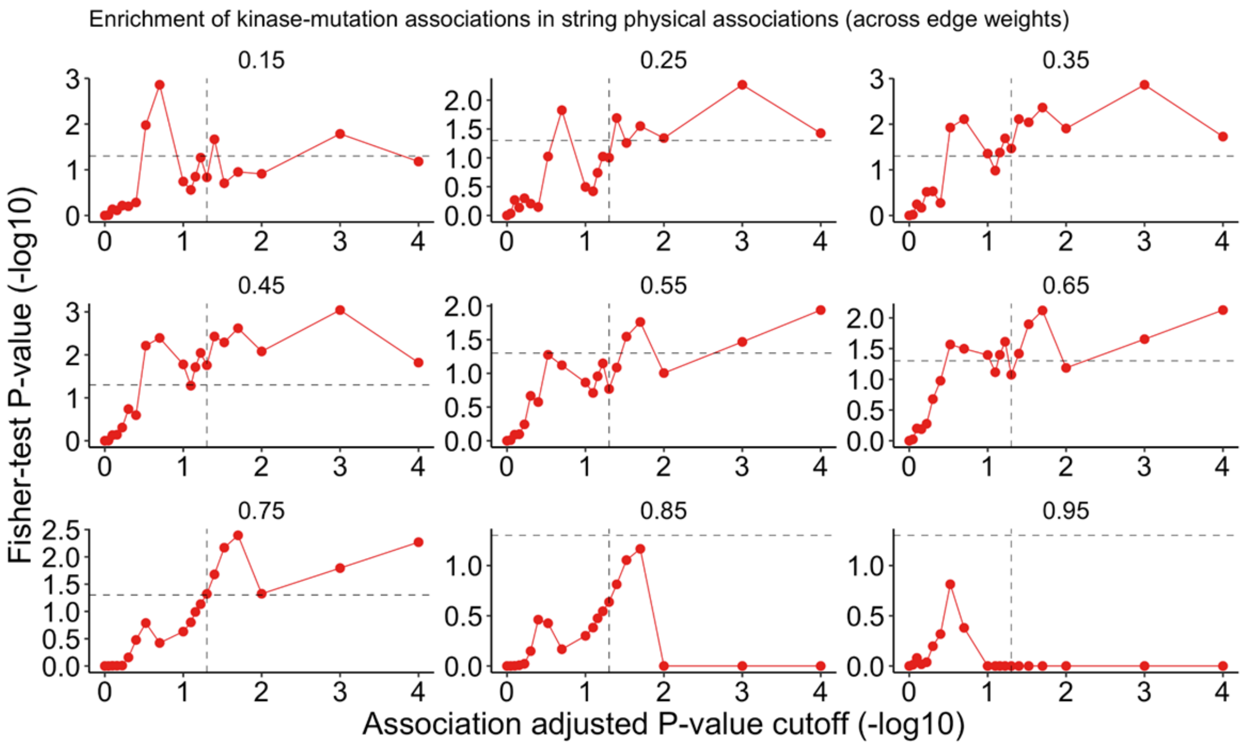

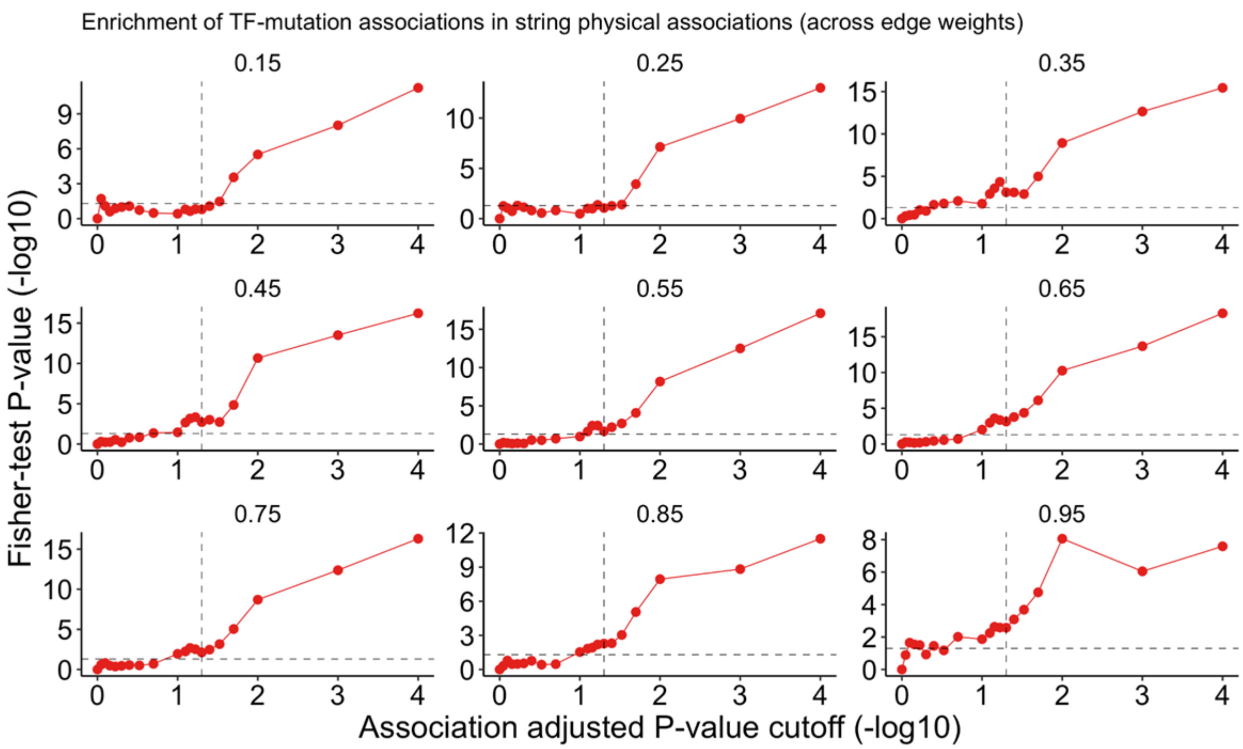
Enrichment of TF-mutation associations in string physical associations (across edge weights)
0.15
0.25
0.35
0.45
0.55
0.65
0.75
0.85
0.95
Fisher-test P-value (-log10)
Association adjusted P-value cutoff (-log10)

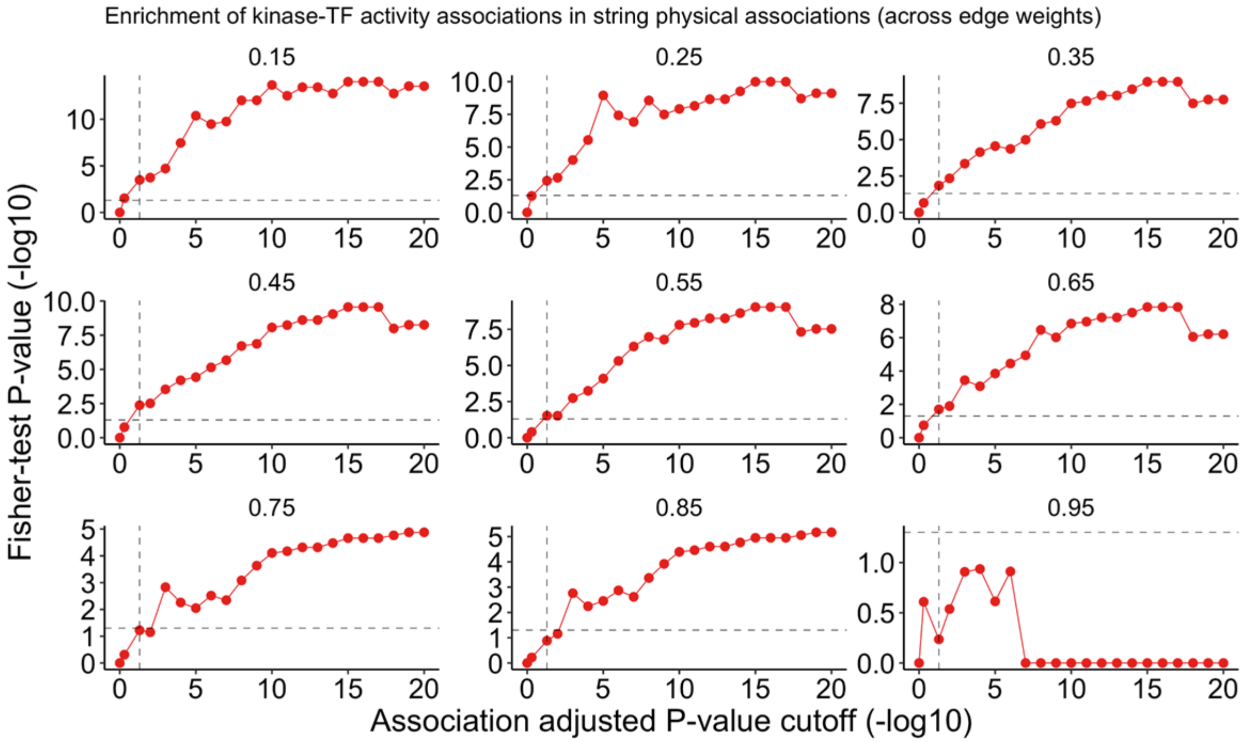
Enrichment of kinase-TF activity associations in string physical associations (across edge weights)
0.15
0.25
0.35
0.45
0.55
0.65
0.75
0.85
0.95
Fisher-test P-value (-log10)
Association adjusted P-value cutoff (-log10)

Reviewer #1 (Significance (Required)):

(integrated above)

Reviewer #2 (Evidence, reproducibility and clarity (Required)):

The study by Sousa, et al. describes analysis of a large multi-omics dataset from nine different cancer types to elucidate relationships between genetic mutations and kinase and transcription factor activity. The authors go through a number of different comparisons that reveal interesting and sometimes surprising things about the biology of the tumors and signaling pathways. I have the following suggestions for improvement.

Comments

1. The results in the paper are convincing overall and highlight some important potins of biological interest - maybe the most important being that loss of function mutations do not manifest as changes in kinase activity. Though a caveat of this finding is that the analysis is on bulk tumor samples, and that different conclusions might be drawn if looking at single cells (or smaller samples) - the authors state this and there are not yet appropriate datasets/techniques to analyze global phosphorylation patterns in very small samples.
We have replicated many of the findings in cell line data where at least there won't be an issue with different populations of cells. We can't however yet analyze data at single cell level so it is possible that the population nature of the analysis masks effects seen only in some sets of cells. As the reviewer notes, we have tried to caution the reader about this caveat but can further expand on it if the reviewer feels it is not sufficiently covered in the discussion.

2. The analysis described in the paper is quite dense and a number of sections need to be revised to improve clarity:
a. On page 4, second paragraph: the correlation described is between 'each protein and phosphosites that mapped to that protein' which is fairly clear, but the next sentence that describes first 'a correlation [across all pairs]' and then 'an average protein-phosphosite correlation' is confusing. It's not clear how these are different but one can guess that the first is talking about intra-protein correlation or intra-phosphosite correlation?
One measure is the correlation observed across all data and the other metric was the average of the correlations observed in each tumor type independently. We simplified this to only mention the pan-cancer value.

b. On page 5, third paragraph: This paragraph is just unclear what comparisons are being made and what the results are. How is 'strongly regulated' defined? CNV and mRNA are mentioned, but then correlation is only discussed for the TFs, not for the kinases, which makes it confusing - I assume that the correlation with kinases is low, but that should be reported.
Strong regulation was defined as a percentile cut-off that we now include in the manuscript. We have tried to make these results clearer by indicating also cases where correlations were poor.

c. Page 7, second paragraph: The first few sentences describe quite a lot of analysis in a very condensed form. I suggest it be expanded to help clarity.
We have added a sentence to better introduce the analysis.

d. Page 10, third paragraph: It was difficult to understand what was being described in this paragraph.
We have tried to improve the description of this section but we welcome more directed suggestions.

e. Page 11, first paragraph: the introduction of "cancer and non-cancer conditions" is very abrupt as the dataset being used was not adequately described (just "large panel of other conditions"). It is not clear what "a correlation between the degree of regulation of kinases in cancer and non-cancer conditions" means. How is this comparison done? The mention of inter-tissue variation is also not well-described.
We have tried to expand and improve the description of how the analysis was done.

f. Page 11, second paragraph. It is not clear how the samples were clustered - though it's described in Method, some more detail should be included in the Results section to give the reader an idea.
We have tried to improve the description of this section.

3. Throughout the paper and in the abstract the number of samples are listed several times with different numbers. I realize that this is likely because of the availability of data limiting some analyses - but the differences in numbers are distracting and confusing. It should be made clear how/why the numbers of samples are reduced for different analyses.
The reviewer is correct that the differences are due to how much data is available for each omics and as a consequence the data that is available to different analyses. We have tried to clearly state that in each analysis we compare samples for which the relevant omics data are available. Here is what we wrote at the start of the results section:
"In summary, the assembled data includes 1,110 tumours samples and 77 cell lines, of which 1008 samples (932 tumours and 76 cell lines) have available data for all data types used in this study. For each analysis we used all samples having data for the types of omics measurement required which results in small variations of the total number of samples used."

4. The comparison of RPPA with MS results is somewhat interesting, but doesn't seem to further the findings of the paper in any significant way. I would suggest that it be removed OR moved to a supplemental figure.
We have moved this panel to a supplementary figure. We think it is useful to show that a very different technology shows some agreement with the mass spectrometry measurements.

5. I found the summary of numbers of samples of different types in Figure 1A to be not terribly helpful. I think a table listing these numbers might be a more efficient way to convey the message here.

We have generated a simpler bar plot in figure 1A that summarizes the number of samples having data for all data types and moved the previous barplot to supplementary.

6. Figure 3C in the text is described as showing "that highly regulated kinases in the same pathway tend to be activated or inhibited across the same samples". The sentence following this one clarifies that this is for one pathway in particular - and that would be good to make clear (rather than the much more general way it's stated now).
The reviewer is correct that Figure 3C is in fact representing a specific pathway and we intended the sentence to describe a general observation that summarizes the different pieces of evidence for this section of the results. We revised this section to make the text clearer and to be specific about what Figure 3C represents.

7. Page 11, second paragraph. The clustering is a potentially very useful piece of information - but there are no figures or other evidence presented that about this analysis, only a description of the different cluster numbers and their enrichment (what does that mean?)
We provide the clustering figures and description as a supplementary set of results (Supplementary Results). We decided to present the results in this way because we have done an extensive review of the clusters but considered that this was a descriptive analysis that although potentially useful it was difficult to extend it beyond the mere description. We decided therefore to keep the full description in a Supplementary Results section and highlight in the main text some of the most interesting observations. We are willing to move some of these figures to supplementary or main figures if the reviewer feels it would make sense although we think the manuscript is already quite extensive.

8. The finding that mutations in kinases don't seem to manifest in reductions in their activity is a surprising and even provocative result. I think this portion of the analysis deserves further investigation - LoF mutations cause a reduction in protein abundance, protein abundance is poorly correlated with activity, LoF is also poorly correlated with activity, but other kinases complement. I guess a direct comparison of the effects of LoF versus protein abundance on activity would be useful. That is, how much of the effect of the LoF mutation on activity can be explained by the reduction in protein abundance? What could the other portion be explained by? The compensatory response explanation is very interesting, but is somewhat unsatisfying without a proposed mechanism (though it's not clear to me what that mechanism might be) and more information about that response.
We agree this is the most surprising aspect of our work which was also noted by the other reviewer. This has been in fact the aspect of the work that we devoted most attention to given that it is, as the reviewer notes, a provocative result. Together with the comments of the first reviewer we have tried to look into more detail into the known activating mutations BRAF V600E, KRAS G12D and KRAS G12C. We would direct the reviewer to the answer given to reviewer 1 in point number 2 that we will summarize here. Briefly, for BRAF V600E we don’t see the expected increase in activity of the “downstream” pathway members and we tried to see if there were possible mechanistic explanations. For this we have selected the 19 samples with this mutation and compared the samples where BRAF activity was increased (6 samples) with

samples where the BRAF activity was predicted to be decreased (9 samples) to look for changes in expression of phosphatases. Of 237 phosphatases tested, 74% tend to have higher expression levels in the samples with low BRAF activity, 17 of these have a significant difference across the two groups of samples with a false discovery rate set at 15% (figure shown in the response to reviewer 1). From this analysis we can extract the phosphatases that show the strongest correlation between their expression and the activation state of BRAF that could indicate an adaptation mechanism in the mutated BRAF samples. This illustrates how these analyses could be used to generate hypotheses about the cell signaling adaptation to mutations although at this stage the sample sizes might not be enough to generate such hypotheses for many mutations.

9. In the current study the authors show that phosphorylation of activating sites on kinases are generally correlated with activity. And previously (Ochoa, et al. 2016) the authors had reported that some specific activating sites on kinases correlated well with their activity. However, Arshad 2019 pointed out cases for which this was not observed in subsets of the data used here. It would therefore be interesting to have more analysis of individual cases of activating (and inhibitory sites) on kinases and how well their phosphorylation reflected activity in the data. This seems to be an important point of regulation that may not always behave in a straightforward way (similar to the LoF finding here) - even if it does correlate on average over lots of kinases.
We thank the reviewer for the interesting idea. We have also noted that such kinase regulatory phosphosites don't always correlate with the phosphorylation levels of their target. We agree that this is a relevant and interesting line of research where we could potentially better predict the code of phosphosite regulation for different kinases but we just didn't have the opportunity to perform the analysis. We may attempt to do a more systematic analysis in the future, ideally paired with some experimental validation.

10. There are a few misspellings and grammatical mistakes that should be corrected:
a. Abstract: "...mutational profiles of thousands of tumors have been extensively characterized..."
b. Page 7. "Similar to our previous reports..."
c. Page 7. "...the genetic alterations are often recapitulated at the protein..."
d. Page 16. "In addition, kinases are very tractable drug targets..."
We have tried to correct the manuscript more carefully.

Reviewer #2 (Significance (Required)):

Much of the understanding of cancer signaling and the impact of driver mutations is based on observations from genomics, and the current work shows that these assumptions might be incorrect in important ways - specifically that loss of function mutations result in direct downregulation of the kinase activity. Since kinases are common targets of drugs and drug development, and compensatory mechanisms (as suggested here) are important in understanding drug response and drug resistance, the significance of this study and it's view across many different cancer types, is fairly significant for our understanding.

I am a computational biologist who has helped generate some of the data used in this study and has published in the area of multi-omic integration to understand cancer signaling and kinase activity.

We thank the reviewer for the positive remarks regarding the significance of the work.

1st Revision - Editorial Decision 7th Nov 2022

Thank you again for submitting your revised study to Molecular Systems Biology along with the referee reports from Review Commons. We have now heard back from the two reviewers who were asked to evaluate your revised study. As you will see below, the reviewers are satisfied with the performed revisions and are supportive of publication in Molecular Systems Biology. However, they list a few remaining concerns, all of which are relatively minor. We would ask you to address them in a minor revision.

Reviewer #1:

The authors have improved their manuscript and added more data to support their findings. Overall I am supportive of acceptance for publication. However, one suggestion remains regarding my earlier comment on tumor heterogeneity and accounting for cancer types in their pan-cancer analysis (comment 1):

I agree in general that the covariate approach described by the authors takes care of some of the extra variation of including multiple cancer types in the same analysis. However, this analysis can backfire in edge cases where there are very few samples (e.g. 5 samples with mutation, each of a different cancer type; vs many samples lacking mutations, representing various cancer types). Then the model will have many poorly defined coefficients and the results may be difficult to interpret. The more reliable workround would focus on one cancer type at a time such that at least some case samples (eg. 2-3 mutated samples) are present. However, fewer hypotheses can be tested due to data sparsity.

Admittedly, this is the type of limited heterogeneous data we have to handle due to a lack of larger cohorts in the public domain. Having larger such multi-omics datasets in the future may help find specific genomic/signalling interactions.

I would recommend that the authors add a paragraph to the discussion section to describe this potential limitation/caveat of the current pan-cancer analysis. Having the perspective of finding more interactions in future datasets would strike an optimistic note.

Reviewer #2:

The authors have addressed my points. I only have the following minor comments reading the revised version:

P3. Typo, the word "kinases" is missing: "We estimated the activities of TFs and kinases from the gene expression levels and phosphorylation changes of their targets, deriving activity profiles of 292 TFs and 218 ___. We"

P14. "Interestingly, we also found that high activity of FOXA1 and FOXM1 is a good and poor prognostic factor in liver cancer, respectively ..." - this is confusingly worded. Maybe better as "Interestingly, we also found that high activity of FOXA1 was a good prognostic factor and FOXM1 was a poor prognostic factor in liver cancer."

Reviewer comments:
Reviewer #1:

The authors have improved their manuscript and added more data to support their findings. Overall I am supportive of acceptance for publication. However, one suggestion remains regarding my earlier comment on tumor heterogeneity and accounting for cancer types in their pan-cancer analysis (comment 1):

I agree in general that the covariate approach described by the authors takes care of some of the extra variation of including multiple cancer types in the same analysis. However, this analysis can backfire in edge cases where there are very few samples (e.g. 5 samples with mutation, each of a different cancer type; vs many samples lacking mutations, representing various cancer types). Then the model will have many poorly defined coefficients and the results may be difficult to interpret. The more reliable workround would focus on one cancer type at a time such that at least some case samples (eg. 2-3 mutated samples) are present. However, fewer hypotheses can be tested due to data sparsity.

Admittedly, this is the type of limited heterogeneous data we have to handle due to a lack of larger cohorts in the public domain. Having larger such multi-omics datasets in the future may help find specific genomic/signalling interactions.

I would recommend that the authors add a paragraph to the discussion section to describe this potential limitation/caveat of the current pan-cancer analysis. Having the perspective of finding more interactions in future datasets would strike an optimistic note.

We thank the reviewer for the suggestion. We have added to the discussion a paragraph discussing the implications of tumour heterogeneities for our analyses and the perspective of using single-cell multi-omics profiling to address them.

Reviewer #2:

The authors have addressed my points. I only have the following minor comments reading the revised version:

P3. Typo, the word "kinases" is missing: "We estimated the activities of TFs and kinases from the gene expression levels and phosphorylation changes of their targets, deriving activity profiles of 292 TFs and 218 ___. We"

We thank the reviewer for the correction. We have added the missing word to the text.

P14. "Interestingly, we also found that high activity of FOXA1 and FOXM1 is a good and poor prognostic factor in liver cancer, respectively ..." - this is confusingly worded. Maybe better as "Interestingly, we also found that high activity of FOXA1 was a good prognostic factor and FOXM1 was a poor prognostic factor in liver cancer."

We thank the reviewer for the suggestion. We accepted the reviewer’s suggestion and changed the sentence in the manuscript.

Thank you again for sending us your revised manuscript. We are now satisfied with the modifications made and I am pleased to inform you that your paper has been accepted for publication.

EMBO Press Author Checklist

Corresponding Author Name: Pedro Beltrao
Journal Submitted to: Molecular Systems Biology
Manuscript Number: MSB-2021-10631

USEFUL LINKS FOR COMPLETING THIS FORM
The EMBO Journal - Author Guidelines
EMBO Reports - Author Guidelines
Molecular Systems Biology - Author Guidelines
EMBO Molecular Medicine - Author Guidelines

Reporting Checklist for Life Science Articles (updated January 2022)
This checklist is adapted from Materials Design Analysis Reporting (MDAR) Checklist for Authors. MDAR establishes a minimum set of requirements in transparent reporting in the life sciences (see Statement of Task: 10.31222/osf.io/9sm4x). Please follow the journal's guidelines in preparing your manuscript.
Please note that a copy of this checklist will be published alongside your article.

Abridged guidelines for figures
1. Data
The data shown in figures should satisfy the following conditions:
- the data were obtained and processed according to the field's best practice and are presented to reflect the results of the experiments in an accurate and unbiased manner.
- ideally, figure panels should include only measurements that are directly comparable to each other and obtained with the same assay.
- plots include clearly labeled error bars for independent experiments and sample sizes. Unless justified, error bars should not be shown for technical replicates.
- if n<5, the individual data points from each experiment should be plotted. Any statistical test employed should be justified.
- Source Data should be included to report the data underlying figures according to the guidelines set out in the authorship guidelines on Data Presentation.

2. Captions
Each figure caption should contain the following information, for each panel where they are relevant:
- a specification of the experimental system investigated (eg cell line, species name).
- the assay(s) and method(s) used to carry out the reported observations and measurements.
- an explicit mention of the biological and chemical entity(ies) that are being measured.
- an explicit mention of the biological and chemical entity(ies) that are altered/varied/perturbed in a controlled manner.
- the exact sample size (n) for each experimental group/condition, given as a number, not a range;
- a description of the sample collection allowing the reader to understand whether the samples represent technical or biological replicates (including how many animals, litters, cultures, etc.).
- a statement of how many times the experiment shown was independently replicated in the laboratory.
- definitions of statistical methods and measures:

- common tests, such as t-test (please specify whether paired vs. unpaired), simple χ2 tests, Wilcoxon and Mann-Whitney tests, can be unambiguously identified by name only, but more complex techniques should be described in the methods section;

- are tests one-sided or two-sided?
- are there adjustments for multiple comparisons?
- exact statistical test results, e.g., P values = x but not P values < x;
- definition of 'center values' as median or average;
- definition of error bars as s.d. or s.e.m.

Please complete ALL of the questions below.
Select "Not Applicable" only when the requested information is not relevant for your study.

Materials

Newly Created Materials	Information included in the manuscript?	In which section is the information available? (Reagents and Tools Table, Materials and Methods, Figures, Data Availability Section)
New materials and reagents need to be available; do any restrictions apply?	Not Applicable	

Antibodies	Information included in the manuscript?	In which section is the information available? (Reagents and Tools Table, Materials and Methods, Figures, Data Availability Section)
For **antibodies** provide the following information:<br>- Commercial antibodies: RRID (if possible) or supplier name, catalogue number and or/clone number<br>- Non-commercial: RRID or citation	Not Applicable	

DNA and RNA sequences	Information included in the manuscript?	In which section is the information available? (Reagents and Tools Table, Materials and Methods, Figures, Data Availability Section)
**Short novel DNA or RNA including primers, probes**: provide the sequences.	Not Applicable	

Cell materials	Information included in the manuscript?	In which section is the information available? (Reagents and Tools Table, Materials and Methods, Figures, Data Availability Section)
**Cell lines:** Provide species information, strain. Provide accession number in repository **OR** supplier name, catalog number, clone number, and/**OR** RRID.	Not Applicable	
**Primary cultures:** Provide species, strain, sex of origin, genetic modification status.	Not Applicable	
Report if the cell lines were recently **authenticated** (e.g., by STR profiling) and tested for mycoplasma contamination.	Not Applicable	

Experimental animals	Information included in the manuscript?	In which section is the information available? (Reagents and Tools Table, Materials and Methods, Figures, Data Availability Section)
**Laboratory animals or Model organisms:** Provide species, strain, sex, age, genetic modification status. Provide accession number in repository **OR** supplier name, catalog number, clone number, **OR** RRID.	Not Applicable	
**Animal observed in or captured from the field:** Provide species, sex, and age where possible.	Not Applicable	
Please detail **housing and husbandry conditions**.	Not Applicable	

Plants and microbes	Information included in the manuscript?	In which section is the information available? (Reagents and Tools Table, Materials and Methods, Figures, Data Availability Section)
**Plants:** provide species and strain, ecotype and cultivar where relevant, unique accession number if available, and source (including location for collected wild specimens).	Not Applicable	
**Microbes:** provide species and strain, unique accession number if available, and source.	Not Applicable	

Human research participants	Information included in the manuscript?	In which section is the information available? (Reagents and Tools Table, Materials and Methods, Figures, Data Availability Section)
If collected and within the bounds of privacy constraints report on age, sex and gender or ethnicity for all study participants.	Not Applicable	

Core facilities	Information included in the manuscript?	In which section is the information available? (Reagents and Tools Table, Materials and Methods, Figures, Data Availability Section)
If your work benefited from core facilities, was their service mentioned in the acknowledgments section?	Not Applicable	

Design

Study protocol	Information included in the manuscript?	In which section is the information available? (Reagents and Tools Table, Materials and Methods, Figures, Data Availability Section)
If study protocol has been **pre-registered, provide DOI in the manuscript**. For clinical trials, provide the trial registration number **OR** cite DOI.	Not Applicable	
Report the **clinical trial registration number** (at ClinicalTrials.gov or equivalent), where applicable.	Not Applicable	

Laboratory protocol	Information included in the manuscript?	In which section is the information available? (Reagents and Tools Table, Materials and Methods, Figures, Data Availability Section)
Provide DOI OR other citation details if **external detailed step-by-step protocols** are available.	Not Applicable	

Experimental study design and statistics	Information included in the manuscript?	In which section is the information available? (Reagents and Tools Table, Materials and Methods, Figures, Data Availability Section)
Include a statement about **sample size** estimate even if no statistical methods were used.	Not Applicable	
Were any steps taken to minimize the effects of subjective bias when allocating animals/samples to treatment (e.g. **randomization procedure**)? If yes, have they been described?	Not Applicable	
Include a statement about **blinding** even if no blinding was done.	Not Applicable	
Describe **inclusion/exclusion criteria** if samples or animals were excluded from the analysis. Were the criteria pre-established? If sample or data points were omitted from analysis, report if this was due to attrition or intentional exclusion and provide justification.	Not Applicable	
For every figure, are **statistical tests** justified as appropriate? Do the data meet the assumptions of the tests (e.g., normal distribution)? Describe any methods used to assess it. Is there an estimate of variation within each group of data? Is the variance similar between the groups that are being statistically compared?	Yes	The statistical tests used are described in the Methods section.

Sample definition and in-laboratory replication	Information included in the manuscript?	In which section is the information available? (Reagents and Tools Table, Materials and Methods, Figures, Data Availability Section)
In the figure legends: state number of times the experiment was **replicated** in laboratory.	Not Applicable	
In the figure legends: define whether data describe **technical or biological replicates**.	Not Applicable	

Ethics

Ethics	Information included in the manuscript?	In which section is the information available? (Reagents and Tools Table, Materials and Methods, Figures, Data Availability Section)
Studies involving **human participants**: State details of **authority granting ethics approval** (IRB or equivalent committee(s), provide reference number for approval.	Not Applicable	
Studies involving **human participants**: Include a statement confirming that **informed consent** was obtained from all subjects and that the experiments conformed to the principles set out in the WMA Declaration of Helsinki and the Department of Health and Human Services Belmont Report.	Not Applicable	
Studies involving **human participants:** For publication of **patient photos**, include a statement confirming that consent to publish was obtained.	Not Applicable	
Studies involving experimental **animals**: State details of **authority granting ethics approva**l (IRB or equivalent committee(s), provide reference number for approval. Include a statement of compliance with ethical regulations.	Not Applicable	
Studies involving **specimen and field samples:** State if relevant **permits** obtained, provide details of authority approving study; if none were required, explain why.	Not Applicable	

Dual Use Research of Concern (DURC)	Information included in the manuscript?	In which section is the information available? (Reagents and Tools Table, Materials and Methods, Figures, Data Availability Section)
Could your study fall under dual use research restrictions? Please check biosecurity documents and list of **select agents and toxins** (CDC): https://www.selectagents.gov/sat/list.htm	Not Applicable	
If you used a select agent, is the security level of the lab appropriate and reported in the manuscript?	Not Applicable	
If a study is subject to dual use research of concern regulations, is the name of the **authority granting approval and reference number** for the regulatory approval provided in the manuscript?	Not Applicable	

Reporting

The MDAR framework recommends adoption of discipline-specific guidelines, established and endorsed through community initiatives. Journals have their own policy about requiring specific guidelines and recommendations to complement MDAR.

Adherence to community standards	Information included in the manuscript?	In which section is the information available? (Reagents and Tools Table, Materials and Methods, Figures, Data Availability Section)
State if relevant guidelines or checklists (e.g., **ICMJE, MIBBI, ARRIVE, PRISMA**) have been followed or provided.	Not Applicable	
For **tumor marker prognostic studies,** we recommend that you follow the **REMARK** reporting guidelines (see link list at top right). See author guidelines, under 'Reporting Guidelines'. Please confirm you have followed these guidelines.	Not Applicable	
For **phase II and III randomized controlled trials**, please refer to the **CONSORT** flow diagram (see link list at top right) and submit the CONSORT checklist (see link list at top right) with your submission. See author guidelines, under 'Reporting Guidelines'. Please confirm you have submitted this list.	Not Applicable	

Data Availability

Data availability	Information included in the manuscript?	In which section is the information available? (Reagents and Tools Table, Materials and Methods, Figures, Data Availability Section)
Have **primary datasets** been deposited according to the journal's guidelines (see 'Data Deposition' section) and the respective accession numbers provided in the Data Availability Section?	Not Applicable	
Were **human clinical and genomic datasets** deposited in a public access-controlled repository in accordance to ethical obligations to the patients and to the applicable consent agreement?	Not Applicable	
Are **computational models** that are central and integral to a study available without restrictions in a machine-readable form? Were the relevant accession numbers or links provided?	Not Applicable	
If publicly available data were reused, provide the respective **data citations in the reference list**.	Yes	All of the publicaly available datasets used in the study were cited in the Methods section
